# The Health Gym: synthetic health-related datasets for the development of reinforcement learning algorithms

**DOI:** 10.1038/s41597-022-01784-7

**Published:** 2022-11-11

**Authors:** Nicholas I-Hsien Kuo, Mark N. Polizzotto, Simon Finfer, Federico Garcia, Anders Sönnerborg, Maurizio Zazzi, Michael Böhm, Rolf Kaiser, Louisa Jorm, Sebastiano Barbieri

**Affiliations:** 1 Centre for Big Data Research in Health, University of New South Wales, Sydney, Australia; 2 Australian National University, Canberra, Australia; 3 The George Institute for Global Health, Sydney, Australia; 4 University of New South Wales, Sydney, Australia; 5 Imperial College London, London, United Kingdom; 6 Hospital Universitario San Cecilio, Granada, Spain; 7 Karolinska Institutet, Stockholm, Sweden; 8 Università degli Studi di Siena, Siena, Italy; 9 Uniklinik Köln, Universität zu Köln, Cologne, Germany

**Keywords:** Computational models, Data publication and archiving

## Abstract

In recent years, the machine learning research community has benefited tremendously from the availability of openly accessible benchmark datasets. Clinical data are usually not openly available due to their confidential nature. This has hampered the development of reproducible and generalisable machine learning applications in health care. Here we introduce the Health Gym - a growing collection of highly realistic synthetic medical datasets that can be freely accessed to prototype, evaluate, and compare machine learning algorithms, with a specific focus on reinforcement learning. The three synthetic datasets described in this paper present patient cohorts with acute hypotension and sepsis in the intensive care unit, and people with human immunodeficiency virus (HIV) receiving antiretroviral therapy. The datasets were created using a novel generative adversarial network (GAN). The distributions of variables, and correlations between variables and trends in variables over time in the synthetic datasets mirror those in the real datasets. Furthermore, the risk of sensitive information disclosure associated with the public distribution of the synthetic datasets is estimated to be very low.

## Abstract (summary)

**Table:** 

Measurement(s)	vital signs • administered fluid boluses • vasopressors
Technology Type(s)	synthetic electronic medical records

## Background & Summary

*Reinforcement learning*^1^ (RL) is an area of artificial intelligence (AI) which learns a behavioural *policy*–a mapping from states to actions–which maximises a cumulative reward in an evolving environment. Recent studies that combine RL with neural networks have achieved super-human performances in tasks from video games^2^ to complex board games^3^. The success of RL was greatly facilitated by the availability of *standard benchmark problems*: tasks with publicly available datasets which allowed the research community to develop, test, and compare RL algorithms (*e.g*., OpenAI Gym^4^, DeepMind Lab^5^, and D4RL^6^). Health-related data is, however, not as easily accessible due to privacy concerns around the disclosure of private information. To address this challenge, this paper introduces the **Health Gym** project–a collection of highly realistic synthetic medical datasets that can be freely accessed to facilitate the development of machine learning (ML) algorithms, with a specific focus on RL.

### Reinforcement learning for health care: promises and challenges

Clinicians treating individuals with chronic disorders (*e.g*., human immunodeficiency virus (HIV) infection) or with potentially life-threatening conditions (*e.g*., sepsis) often prescribe a series of treatments to maximise the chances of favourable outcomes. This generally requires modifying the duration, dosage, or type of treatment over time; and is challenging due to patient heterogeneity in responses to treatments, potential relapses, and side-effects. Clinicians often rely, at least in part, on clinical judgement to prescribe sequences of treatments, because the clinical evidence base is incomplete and available evidence may not represent the diversity of real-life clinical states. There is thus vast potential for RL algorithms to optimise personalised treatment regimens, as shown by early research on antiretroviral therapy in HIV^7,8^, radiotherapy planning in lung cancer^9^, and management of sepsis^10^. Nonetheless, some authors have highlighted the lack of reproducibility and potential for patient harm inherent in these methods^11^. In particular, recommendations made by RL algorithms may not be safe if the training data omit variables that influence clinical decision making, or if the effective sample size is small^12^.

One of the main difficulties in developing robust RL algorithms for healthcare is the highly confidential nature of clinical data. Researchers are often required to establish formal collaborations and execute extensive data use agreements before sharing data. One approach to overcome these barriers is to generate synthetic data that closely resembles the original dataset but does not allow re-identification of individual patients and can therefore be freely distributed. Synthetic data generation has previously been applied to computed tomography images^13^ and electronic health records^14^; and early studies found that both linear^15^ and non-linear^16^ models could generate continuous and categorical variables. More recently, deep learning techniques such as *Generative Adversarial Networks*^17^ (GANs) have also been used to generate realistic medical time series^18^.

### The health gym project

The Health Gym project is a growing collection of synthetic but realistic datasets for developing RL algorithms. Here we introduce the first three datasets related to the management of *acute hypotension*^19^, *sepsis*^10^, and *HIV*^20^. All datasets were generated using GANs and the MIMIC-III^21,22^ and EuResist^23^ databases. MIMIC-III comprises health-related data for patients who stayed in intensive care units (ICUs) of the Beth Israel Deaconess Medical Centre (Boston, USA) between 2001 and 2012. Within MIMIC-III, we identified two cohorts of patients: 3,910 patients with acute hypotension and 2,164 patients with sepsis. Similarly, the EuResist Integrated Database was used to extract longitudinal information related to 8,916 people with HIV. For acute hypotension and sepsis, we extracted the related timeseries of vital signs, laboratory test results, medications (*e.g*., administered intravenous fluids and vasopressors), and demographics. For people with HIV, we included demographics and time series of antiretroviral medications, cluster of differentiation 4 + T-lymphocytes (CD4) count, and viral load measurements.

Both MIMIC-III and EuResist contain only de-identified data (*i.e*., personal identifiers have been removed and other pre-processing steps such as date shifting were applied to minimise disclosure risk); however, there is a small remaining risk that personal information may be disclosed if an “attacker” or “adversary” (a person or process seeking to learn sensitive information about an individual) is able to link our published data back to personal identifiers. To minimise this risk, we evaluated the synthetic data using current best practices^24^ in terms of both membership disclosure (*i.e*., that no record in the synthetic data can be mapped directly to a record in the real data) and attribute disclosure (*i.e*., that even if part of the data are known to an attacker, the remaining attributes cannot be recovered exactly). In the Usage Notes section, we provide a broader impact statement to discuss the implementations and applications of our work.

## Methods

### Cohort selection

This study was approved by the University of New South Wales’ human research ethics committee (application HC210661). For patients in MIMIC-III requirement for individual consent was waived because the project did not impact clinical care and all protected health information was deidentified^21^. For people in the EuResist integrated database all data providers obtained informed consent for the execution of retrospective studies and inclusion in merged cohorts^25^.

In this work, we applied GANs to longitudinal data extracted from the MIMIC-III^21^ and EuResist^23^ databases to generate three synthetic datasets. The inclusion and exclusion criteria used to define the patient cohorts were adapted from previous studies: Gottesman *et al*.^19^ for defining the patient cohort with acute hypotension, Komorowski *et al*.^10^ to define the sepsis cohort, and Parbhoo *et al*.^20^ to define the HIV cohort. Further details are provided below with technical details documented in Section 1 of the Supplementary Materials. Our synthetic datasets thus include variables that can be used to define the observations, actions, and rewards associated with RL problems for the management of these clinical conditions.

In order to describe our data generation procedure, the rest of this section starts by describing the real datasets and then provides details on our neural network design for generating the synthetic datasets. Our synthetic datasets include all variables in their real counterparts, in the identical formats, and are described in the Data Records section.

### The real datasets

The set of variables contained in each dataset is reported below. Interested readers can find the descriptive statistics (*i.e*., quantiles and mean values) of the real datasets in previous studies: see Feng *et al*.^26^ for the details of MIMIC-III, and see Oette *et al*.^27^ for the details of EuResist.

#### Acute hypotension

The real dataset for the management of acute hypotension was originally proposed in the work of Gottesman *et al*.^19^. It was derived from MIMIC-III and contains the following clinical variables, measured over a 48-hour time period in 3,910 patients:

- mean arterial pressure (MAP), systolic and diastolic blood pressures (SBP and DBPs);
- laboratory results of Alanine and Aspartate Aminotransferase (ALT and AST), lactate, and serum creatinine;
- mechanical ventilation parameters such as partial pressure of oxygen (PaO_2_) and fraction of inspired oxygen (FiO_2_);
- Glasgow Coma Scale (GCS) score^28^;
- administered fluid boluses and vasopressors; and
- urine outputs.

Further details are reported in Table 1. Data were aggregated for every hour in the time series; there are hence 48 data points per variable for each patient. Data missingness in clinical time series is usually highly informative, indicating *e.g*., the need for specific laboratory tests. Hence the real dataset includes variables with suffix *(M)* to indicate whether a variable was measured at a specific point in time.

**Table 1:** Variables in the Acute Hypotension Dataset.

Variable Name	Data Type	Unit	Descriptive Statistics
Mean Arterial Pressure (MAP)	numeric	mmHg	Median: 65.34 (Q1: 59.30, Q3: 71.19)
Diastolic Blood Pressure (DBP)	numeric	mmHg	Median: 54.33 (Q1: 48.37, Q3: 60.26)
Systolic BP (SBP)	numeric	mmHg	Median: 113.21 (Q1: 104.23, Q3: 121.60)
Urine	numeric	mL	Median: 106.21 (Q1: 68.92, Q3: 164.23)
Alanine Aminotransferase (ALT)	numeric	IU/L	Median: 32.55 (Q1: 24.59, Q3: 46.09)
Aspartate Aminotransferase (AST)	numeric	IU/L	Median: 46.82 (Q1: 35.81, Q3: 67.75)
Partial Pressure of Oxygen (PaO_2_)	numeric	mmHg	Median: 103.02 (Q1: 91.34, Q3: 114.66)
Lactate	numeric	mmol/L	Median: 1.50 (Q1: 1.29, Q3: 1.80)
Serum Creatinine	numeric	mg/dL	Median: 1.11 (Q1: 0.83, Q3: 1.62)
Fluid Boluses	categorical	mL	4 Classes
			[0,250):97.32%; [250,500):0.28%
			[500,1000):1.46%; ≥ 1000:0.94%
Vasopressors	categorical	mcg/kg/min	4 Classes
			0:84.14%; (0,8.4):8.34%
			[8.4,20.28):3.68%; ≥ 20.28:3.83%
Fraction of Inspired Oxygen (FiO_2_)	categorical	fraction	10 Classes
			≤ 0.2:0.00%; 0.2:0.54%
			0.3:2.84%; 0.4:10.85%
			0.5:63.30%; 0.6:8.58%
			0.7:1.32%; 0.8:0.20%
			0.9:2.63%; 1.0:9.75%
Glasgow Coma Scale Score (GCS)	categorical	point	13 Classes
			3:6.61% 4:2.16% 5:0.00% 6:3.00%
			7:4.77% 8:0.00% 9:2.22% 10:4.32%
			11:2.46% 12:3.56% 13:1.00%
			14:9.80% 15:60.09%
Urine Data Measured (Urine (M))	binary	—	False: 63.07% True: 36.93%
ALT or AST Data Measured (ALT/AST (M))	binary	—	False: 98.50% True: 1.50%
FiO_2_ (M)	binary	—	False: 92.49% True: 7.51%
GCS (M)	binary	—	False: 81.49% True: 18.51%
PaO_2_ (M)	binary	—	False: 97.56% True: 2.44%
Lactic Acid (M)	binary	—	False: 96.98% True: 3.02%
Serum Creatinine (M)	binary	—	False: 95.26% True: 4.74%

This table presents the variables shared by the real and synthetic datasets for the management of acute hypotension. Those variables with suffix (M) indicate whether a data point has been measured (which is usually highly informative in medical time series). The descriptive statistics in this table are *only* for the synthetic dataset. For the numeric variables, we list the median as well as the first and third quantiles (Q1 and Q3). As for the categorical and binary variables, we report the share of each unique class in the synthetic dataset. The information in this table should be compared with the illustrations of Fig. 3.

In their work, Gottesman *et al*. used this dataset to develop an RL agent which suggested the optimal amounts of fluid boluses and vasopressors for the management of acute hypotension. Notably, they binned both fluid boluses and vasopressors into multiple categories for the RL agent to make decisions in a discrete action space. Section 1.1 of the Supplementary Materials contains the technical details for deriving this real dataset.

#### Sepsis

The real sepsis dataset constructed by Komorowski *et al*.^10^ was also derived from MIMIC-III. It is more complex than the real hypotension dataset and comprises 44 variables, including vital signs, laboratory results, mechanical ventilation information, and various patient measurements. The complete list of variables is reported in Tables 2 and 3.

**Table 2:** Numeric Variables in the Sepsis Dataset.

Variable Name	Data Type	Unit	Descriptive Statistics		
			Median	Q1	Q3
Age	numeric	year	65.40	58.29	72.95
Heart Rate (HR)	numeric	bpm	89.09	78.46	99.82
Systolic BP	numeric	mmHg	123.67	114.43	133.03
Mean BP	numeric	mmHg	81.02	75.18	86.91
Diastolic BP	numeric	mmHg	58.90	50.40	66.95
Respiratory Rate (RR)	numeric	bpm	21.46	18.69	24.28
Potassium (K^+^)	numeric	meq/L	4.12	3.78	4.45
Sodium (Na^+^)	numeric	meq/L	140.01	136.59	143.57
Chloride (Cl^−^)	numeric	meq/L	105.23	102.08	108.03
Calcium (Ca)	numeric	mg/dL	8.02	7.37	8.66
Ionised Ca^++^	numeric	mg/dL	1.11	1.04	1.18
Carbon Dioxide (CO_2_)	numeric	meq/L	25.27	23.44	27.29
Albumin	numeric	g/dL	3.01	2.68	3.32
Hemoglobin (Hb)	numeric	g/dL	10.20	9.17	11.23
Potential of Hydrogen (pH)	numeric	—	7.39	7.34	7.44
Arterial Base Excess (BE)	numeric	meq/L	0.16	−2.04	2.48
Bicarbonate (HCO_3_)	numeric	meq/L	24.38	22.63	26.13
FiO_2_	numeric	fraction	0.45	0.38	0.55
Glucose	numeric	mg/dL	134.11	108.21	167.06
Blood Urea Nitrogen (BUN)	numeric	mg/dL	25.38	19.89	31.92
Creatinine	numeric	mg/dL	1.13	0.90	1.44
Magnesium (Mg^++^)	numeric	mg/dL	2.04	1.83	2.29
Serum Glutamic Oxaloacetic Transaminase (SGOT)	numeric	u/L	50.78	31.53	88.97
Serum Glutamic Pyruvic Transaminase (SGPT)	numeric	u/L	39.99	26.20	65.66
Total Bilirubin (Total Bili)	numeric	mg/dL	1.19	0.66	2.32
White Blood Cell Count (WBC)	numeric	E9/L	10.60	7.99	13.92
Platelets Count (Platelets)	numeric	E9/L	184.44	141.97	239.41
PaO_2_	numeric	mmHg	109.07	84.22	139.63
Partial Pressure of CO_2_ (PaCO_2_)	numeric	mmHg	39.32	34.92	44.97
Lactate	numeric	mmol/L	1.82	1.41	2.40
Total Volume of Intravenous Fluids (Input Total)	numeric	mL	4867.46	1887.84	11155.76
Intravenous Fluids of Each 4-Hour Period (Input 4H)	numeric	mL	58.66	13.83	229.01
Maximum Dose of Vasopressors in 4H (Max Vaso)	numeric	mcg/kg/min	0.0002	0.0	0.0017
Total Volume of Urine Output (Output Total)	numeric	mL	2505.54	585.47	6733.69
Urine Output in 4H (Output 4H)	numeric	mL	159.33	44.74	361.69

The format of this table follows that of Table 1; and with more results in Table 3. Only the first three columns are shared by both the real and synthetic sepsis datasets. The remaining columns show the descriptive statistics that are specific for the synthetic dataset. The content in this table should be compared with the illustrations in Figs. 6 and 7.

**Table 3:** Non-Numeric Variables in the Sepsis Dataset.

Variable Name	Data Type	Unit	Descriptive Statistics
Gender	binary	—	Male: 73.41% Female: 26.59%
Readmission of Patient (Readmission)	binary	—	False: 60.20% True: 39.80%
Mechanical Ventilation (Mech)	binary	—	False: 56.89% True: 43.11%
GCS	categorical	point	13 Classes
			3:8.71% 4:0.38% 5:0.50% 6:6.30%
			7:0.74% 8:2.27% 9:1.52% 10:9.31%
			11:9.12% 12:6.31% 13:2.53%
			14:15.45% 15:36.85%
Pulse Oximetry Saturation (SpO_2_)	categorical	%	10 Classes (C)
			C1: [0.00,93.83):13.38%; C2: [93.83,95.14):8.12%;
			C3: [95.14,96.00):4.48%; C4: [96.00,96.70):10.64%;
			C5: [96.70,97.33):12.61%; C6: [97.33,98.00):11.36%;
			C7: [98.00,98.60):11.52%; C8: [98.60,99.22):11.84%;
			C9: [99.22,99.86):8.39%; C10: [99.86,100.0]:7.66%;
Temperature (Temp)	categorical	Celsius	10 Classes (C)
			C1: [15.11,35.95):7.83%; C2: [35.95,36.28):6.55%;
			C3: [36.28,36.50):12.87%; C4: [36.50,36.69):16.56%;
			C5: [36.69,36.88):4.21%; C6: [36.88,37.06):8.21%;
			C7: [37.06,37.28):7.10%; C8: [37.28,37.56):9.37%;
			C9: [37.56,37.93):10.96%; C10: [37.93,40.52]:16.33%;
Partial Thromboplastin Time (PTT)	categorical	s	10 Classes (C)
			C1: [17.80,24.53):7.69%; C2: [24.53,26.63):6.71%;
			C3: [26.63,28.20):10.02%; C4: [28.20,29.60):12.44%;
			C5: [29.60,31.45):5.46%; C6: [31.45,34.00):9.27%;
			C7: [34.00,37.10):9.99%; C8: [37.10,42.80):11.47%;
			C9: [42.80,57.90):12.38%; C10: [57.90,150.00]:14.58%;
Prothrombin Time (PT)	categorical	s	10 Classes (C)
			C1: [9.90,12.20):7.89%; C2: [12.20,12.90):8.2%;
			C3: [12.90,13.30):11.02%; C4: [13.30,13.80):9.84%;
			C5: [13.80,14.30):9.45%; C6: [14.30,14.90):6.59%;
			C7: [14.90,15.90):10.37%; C8: [15.90,17.51):10.51%;
			C9: [17.51,22.00):13.27%; C10: [22.00,146.70]:12.85%;
International Normalised Ratio (INR)	categorical	—	10 Classes (C)
			C1: [0.00,1.00):0.19%; C2: [1.00,1.10):8.88%;
			C3: [1.10,1.20):23.35%; C4: [2.21,17.60]:0.09%
			C5: [1.20,1.30):15.64%; C6: [1.30,1.31):10.22%;
			C7: [1.31,1.50):7.53%; C8: [1.50,1.70):9.71%;
			C9: [1.70,2.21):10.67%; C10: [2.21,17.60]:13.70%;

The format of this table follows that of Table 1; and it is a continuation of Table 2.

The real sepsis dataset contains time series data for 2,164 patients. However, the duration of hospital stay varies for each patient. The shortest record is 8 hours long and the longest record lasts 80 hours. Furthermore, the data are reported in 4-hour windows^29^; hence, the shortest patient record contains 2 data points, whereas the longest contains 20 data points. Section 1.2 of the Supplementary Materials describes the technical details for deriving the real sepsis dataset.

In their paper, Komorowski *et al*. employed an RL agent to prescribe different doses of intravenous fluids and vasopressors based on a patient’s clinical variables. Their RL agent was trained by assigning rewards depending on whether the patients transitioned to a more favourable health state following the actions taken.

We purposely left out some variables from the work of Komorowski *et al*. in our real sepsis dataset. Namely, we did not include the four items of PaO_2_/FiO_2_ ratio (P/F ratio), shock index, sequential organ failure assessment (SOFA) score, and systemic inflammatory response syndrome (SIRS) criteria. These items were excluded because they can easily be derived from the other variables that are included. We provide further information on deriving these auxiliary variables in Section 1.2.2 of the Supplementary Materials.

#### HIV

Our real HIV dataset is based on the study of Parbhoo *et al*.^20^. In their paper, Parbhoo *et al*. extracted a cohort of people with HIV from the EuResist^23^ database; and proposed a mixture-of-experts approach for the therapy selection for people with HIV. They first used kernel-based methods to identify clusters of similar people, and then they employed an RL agent to optimise the treatment strategy.

Although our real HIV dataset was based on their work, we made additional changes to the real HIV dataset in order to reflect a recent guideline published by the *World Health Organisation* (WHO)^30^ on the standardisation of antiretroviral therapy for HIV. We included 8,916 people from the EuResist database who started therapy after 2015 and were treated with the 50 most common medication combinations, including 21 different types of medications. Refer to Section 1.3.1 of the Supplementary Materials for a discussion on the WHO guideline.

The variables in our real HIV dataset are reported in Table 4. They include demographics, viral load (VL), CD4 counts, and regimen information. VL reflects how much HIV virus is in a person’s body; and this variable allows medical experts to surmise the state of infection, select appropriate medications, and infer the effectiveness of past treatments. CD4 counts measure how many T-cells (a type of white blood cell) are in the body; and can be used to infer the health of the immune system of a person. People with very low CD4 counts are at risk of negative health outcomes. Following the aforementioned WHO guideline, we deconstructed each person’s medication regimen into a collection of categorical variables representing the most commonly used base medication combinations, as well as auxiliary medications from different medication classes.

**Table 4:** Variables in the HIV Dataset.

Variable Name	Data Type	Unit	Descriptive Statistics
Viral Load (VL)	numeric	copies/mL	Median: 54.77 (Q1: 16.51, Q3: 209.03)
Absolute Count for CD4 (CD4)	numeric	cells/μL	Median: 465.81 (Q1: 279.26, Q3: 840.34)
Relative Count for CD4 (Rel CD4)	numeric	cells/μL	Median: 25.57 (Q1: 18.20, Q3: 35.72)
Gender	binary	—	Male: 93.42% Female: 6.58%
Ethnicity	categorical	—	4 Classes
			Asian: 0.47%; African: 2.55%
			Caucasian: 26.81%; Other: 70.17%
Base Drug Combination (Base Drug Combo)	categorical	—	6 Classes
			FTC + TDF: 73.66%; 3TC + ABC 14.08%
			FTC + TAF: 0.98%
			DRV + FTC + TDF: 5.50%
			FTC + RTVB + TDF: 2.30%
			Other: 3.47%
Complementary INI (Comp. INI)	categorical	—	4 Classes
			DTG: 11.96%; RAL: 0.49%
			EVG: 4.69%; Not Applied: 82.86%
Complementary NNRTI (Comp. NNRTI)	categorical	—	4 Classes
			NVP: 0.19%; EFV: 9.27%
			RPV: 43.76%; Not Applied: 46.78%
Extra PI	categorical	—	6 Classes
			DRV: 0.69% RTVB: 4.02%
			LPV: 1.08% RTV: 2.02%
			ATV: 4.26% Not Applied: 87.92%
Extra pk Enhancer (Extra pk-En)	binary	—	False: 96.70% True: 3.30%
VL Measured (VL (M))	binary	—	False: 79.35% True: 20.65%
CD4 (M)	binary	—	False: 83.39% True: 16.61%
Drug Recorded (Drug (M))	binary	—	False: 15.56% True: 84.44%

This table presents the variables shared by the real and synthetic datasets for antiretroviral therapy in HIV. The format of this table follows that of Table 1. Only the first three columns are shared by both the real and synthetic HIV datasets. The last column shows the descriptive statistics that are specific to the synthetic dataset. The acronyms of medication classes are *integrase inhibitors* (INIs), *non-nucleotide reverse transcriptase inhibitors* (NNRTIs), *protease inhibitors* (PIs), and *pharmacokinetic enhancers* (pk-En). Medications of *nucleoside reverse transcriptase inhibitors* (NRTIs) and *nucleotide reverse transcriptase inhibitors* (NtRTIs) are not explicitly listed in the table because they are already included in the base drug combination variable. See further discussion in Section 1.3.3 of the Supplementary Materials. The content in this table should be compared with the illustrations in Fig. 10.

Similar to the real sepsis dataset, the length of therapy in the real HIV dataset varies across people. Thus, we truncated the records and modified their lengths to the closest multiples of 10-month periods. Hence, the real HIV dataset consists of people with 10, 20, 30, etc month-long data. The shortest patient record is 10 months long whereas the longest patient record is 100 months long. Since each data entry summarises patient observations over a 1 month time period, the shortest record is of length 10, and the longest record is of length 100. Similar to the hypotension dataset (Table 1), the real HIV dataset is very sparse and thus we included binary variables with suffix (M) to indicate whether a variable was measured at a specific time. Section 1.3.3 of the Supplementary Materials provides further details on the derivation process of the real HIV dataset.

### The Health Gym GAN

**Fig. 1:**
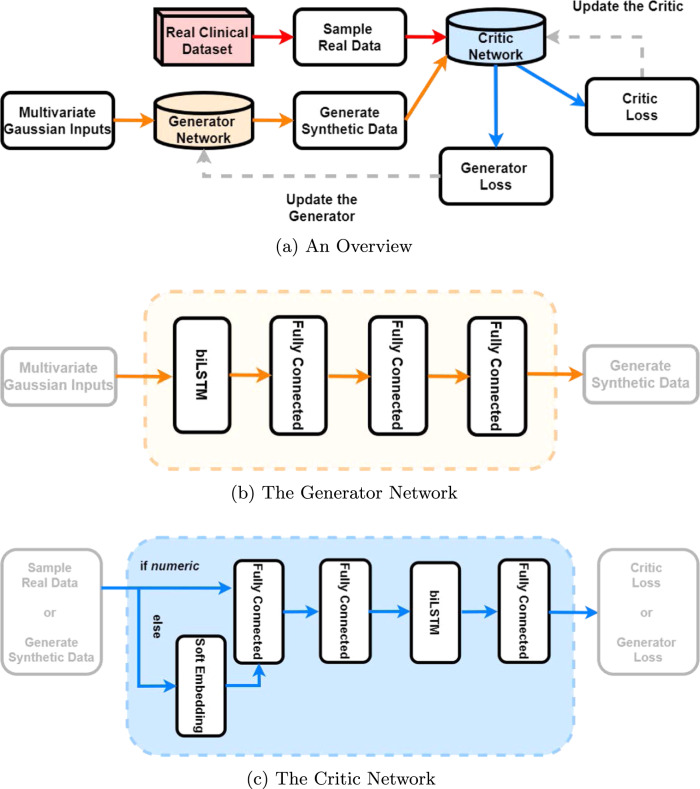
The Wasserstein GAN Pipeline for the Health Gym Project. The overview of the Wasserstein GAN pipeline is shown in (**a**). It conjointly trains a generator network which synthesises data, and a critic network which is optimised to tell the synthetic samples from the real ones. In (**b**), we show that the generator consists of one biLSTM layer followed by three fully conneted dense layers. Whereas in (**c**), the critic first embeds non-numeric data, then it passes all input to two fully connected layers, a biLSTM layer, then another fully connected layer.

The overarching pipeline of the *Generative Adversarial Network*^17^ (GAN) is shown in Fig. 1(a). The setup iteratively and concurrently fine-tunes two networks–the *generator* and the *discriminator* (or *critic*)–to create highly realistic synthetic data.

#### The GAN Setup

The process of training a GAN model can be thought as a two-player-game with two complementary training dynamics. At first, the generator produces synthetic data samples. Then, these synthetic data are compared with samples of the real clinical data by the discriminator. The job of the discriminator is to distinguish between real and synthetic data. A mathematical description of the training procedure for the GAN is reported below. Training is concluded when the discriminator can no longer tell the real and synthetic data apart. That is, a generator is considered to be able to create highly realistic synthetic data when the discriminator is guessing randomly. When the training ends, we use the generator to create our Health Gym synthetic datasets.

In the descriptions below, we denote the generator as *G* and the discriminator as *D*. Furthermore, we use *X*_real_ and *X*_syn_ to represent the real and synthetic datasets; and likewise, we designate *x*_real_ and *x*_syn_ as real and synthetic data batches respectively.

#### The models

As shown in Fig. 1(a), the generator *G* creates the synthetic data based on pseudo-random inputs *z*. The elements of z are sampled from a multivariate Gaussian distribution, and they can be considered latent variables that describe intrinsic aspects of the clinical dataset. The task of network *G* is to transform a time series of latent descriptions into a set of synthetic but realistic time series of clinical variables $G:z\to {x}_{{\rm{syn}}}$.

The intermediate steps of the generator transformation are illustrated in Fig. 1(b). Since the input is a set of latent time series, we employ a bidirectional *Long Short-Term Memory*^31,32^ (biLSTM) *recurrent neural network* (RNN) module to interpolate the relations among the latent features along the time dimension. The RNN is then followed by three fully connected dense layers^33^–high dimensional non-linear transformations responsible for feature extraction and synthetic data construction.

In order to evaluate the realisticness of the synthetic data *x*_syn_, we forward the synthetic data along with a batch of real data *x*_real_ to the discriminator (or critic) *D*. As shown in Fig. 1(c), the discriminator network is also a mixture of recurrent and feedforward modules. To facilitate training, the generator and the discriminator were designed to have a similar number of parameters. Since the input to *D* (both the synthetic data and the real data) contains binary and categorical variables, we use *soft embeddings*^34,35^ to represent them as numeric vectors in a machine-readable format. The discriminator *D* employs two fully connected dense layers to interconnect all features of the data. Then, it employs a biLSTM RNN to interpolate the extrated features along the time dimension; before using a third fully connected dense layer to combine all features to output a realisticness score. Section 2.1 of the Supplementary Materials reports the technical details on network dimensionalities and variable embeddings.

#### Training the GAN model

We adopted the training objective of *Wasserstein GAN with Gradient Penalty*^36,37^ (WGAN-GP) to train our GAN model. The networks were updated using

1 $${\rm{the}}\;{\rm{critic}}\;{\rm{loss}}:{L}_{D}=\mathop{\underbrace{{\mathbb{E}}[D(G(z))-{\mathbb{E}}[D({x}_{{\rm{real}}})]]}}\limits_{{\rm{Wasserstein}}\;{\rm{value}}\;{\rm{function}}}+\mathop{\underbrace{{\lambda }_{{\rm{GP}}}{\mathbb{E}}\,[{({\nabla }_{{x}_{{\rm{syn}}}}D{({x}_{{\rm{syn}}})}_{2}-1)}^{2}]}}\limits_{{\rm{Gradient}}\;{\rm{penalty}}\;{\rm{loss}}}$$

and

2 $${\rm{the}}\;{\rm{generator}}\;{\rm{loss}}:\quad {L}_{G}=-{\mathbb{E}}\left[D(G(z))\right].$$

The critic network was trained by minimising *L*_*D*_; and likewise the generator network was trained by minimising *L*_*G*_. Note, a critic serves a very similar role to a discriminator and hence we used the two terms interchangeably. Specifically, a discriminator is trained to correctly identify *x*_syn_ from *x*_real_, while a critic estimates the distance between *x*_syn_ and *x*_real_.

The first two terms of Eq. (1) form the Wassertein value function^38,39^ which was constructed through the *Kantorovich-Rubinstein duality* theorem^40^. This required the theoretical guarantees on the smoothness of network *D*; in practical terms, this was enforced by the gradient penalty loss term to satisfy the Lipschitz continuity with the gradient normality of 1. Furthermore, the constant *λ*_GP_ served as a regularisation term that controlled the strength of the gradient penalty loss.

An intuitive interpretation of Eqs. (1) and (2) can be obtained by noting that for both losses, the component $D(G(z))$ is identical to $D({x}_{{\rm{syn}}})$. Component $D(G(z))$ can hence be conceptualised as a score of the realisticness of the synthetic data. Thus, the generated data is considered more realistic if Eq. (2) is minimised. In the critic loss of Eq. (1), a two-player-game takes place to make it possible to iteratively fine-tune both subnetworks. The Wasserstein value function leverages the critic network *D* to compare the realisticness of the synthetic data $D(G(z))$ against the ground truth $D({x}_{{\rm{real}}})$. While the generator *G* is trained to fool the critic *D* by maximising the realisticness ${\mathbb{E}}\left[D(G(z))\right]$ (equivalent to minimising $-{\mathbb{E}}\left[D(G(z))\right]$), the critic *D* is fine-tuned to maximise the difference in the realisticness between the real data and the synthetic data ${\mathbb{E}}\left[D({x}_{{\rm{real}}})\right]-{\mathbb{E}}\left[D(G(z))\right]$ (equivalent to minimising ${\mathbb{E}}\left[D(G(z))\right]-{\mathbb{E}}\left[D({x}_{{\rm{real}}})\right]$). This allowed the critic to become better at differentiating between real and synthetic data, and in turn yielded a higher loss in Eq. (2) to further fine-tune the generator *G*.

Prior studies in GANs are mostly focused on generating static images for computer vision tasks. However, our aim for the Health Gym is to generate contiguous time series data. That is, we are concerned with both the realistic distributions of individual variables and the correlation among variables over time. To ensure that correlations among variables are captured correctly by the GAN model, we found it useful to make a slight modification to the generator loss function of Eq. (2). We augmented the vanilla generator loss function as

3 $${L}_{G}=-{\mathbb{E}}\left[D\left(G(z)\right)\right]+\mathop{\underbrace{{\lambda }_{{\rm{corr}}}\mathop{\sum }\limits_{i=1}^{n}\mathop{\sum }\limits_{j=1}^{i-1}{\left\Vert {r}_{{\rm{syn}}}^{(i,j)}-{r}_{{\rm{real}}}^{(i,j)}\right\Vert }_{{L}_{1}}}}\limits_{{\rm{Alignment}}\;{\rm{loss}}}$$

where the additional term is denoted as the alignment loss. We first calculate the *Pearson’s r correlation*^41^ *r*^(*i,j*)^ for every unique pair of variables *X*^(*i*)^ and *X*^(*j*)^; then the alignment loss is calculated as the *L*_1_ loss between the differences in correlations between the synthetic data *r*_syn_ and their real counterparts *r*_real_. Furthermore, *λ*_corr_ is a positive constant which serves as a weight to control the strength of the alignment loss. Section 2.2 of the Supplementary Materials reports more details on the training procedure and on the selection of hyper-parameters.

## Data Records

All of our synthetic datasets are stored as *comma separated value* (CSV) files and are accessible through the Health Gym website (see https://healthgym.ai/). The synthetic hypotension and sepsis datasets are currently hosted on PhysioNet^42,43^–a research resource for complex physiologic signals which also hosts the official MIMIC-III^21,22^ database. The synthetic HIV dataset is on FigShare^44^.

All synthetic datasets follow the formats of their real counterparts which we described in The Real Datasets in Methods. This section describes specific properties of the synthetic datasets. Quality assurance tests are reported later in the Technical Validation section.

### The synthetic hypotension dataset

The synthetic hypotension dataset is 21.7 MB and follows the format of the real hypotension dataset of Gottesman *et al*.^19^ containing 3,910 synthetic patients. Like its real counterpart, there are 48 data points per patient representing time series of 48 hours. There are hence 187,680 (=3,910 × 48) records (rows) in total.

The synthetic hypotension data comprises 22 variables (columns). The first 20 variables are listed in Table 1–there are 9 numeric variables, 4 categorical, and 7 binary variables. The 21^st^ variable contains the synthetic patient IDs and the 22^nd^ variable indicates the hour in the time series. The units and descriptive statistics of the clinical variables are shown in Table 1. The descriptive statistics column shows the first, second, and third quartiles (*i.e*., the 25^th^ percentile, median, and 75^th^ percentile) for the numeric variables; and the share, in percentage, of each unique class for the binary and categorical variables.

The information presented in this table corresponds to the distributions of the synthetic variables in Fig. 3. Several numeric variables (*e.g*., urine, serum creatinine) are right-skewed, whereas binary and categorical variables are heavily class imbalanced. This will likely require variable transformation for downstream machine learning applications. Interested readers may consider our proposed pre-processing scheme in Section 2.1.2 of the Supplementary Materials.

### The synthetic sepsis dataset

The synthetic sepsis dataset is 16 MB and follows the format of the real sepsis dataset of Komorowski *et al*.^10^ containing 2,164 synthetic patients. The synthetic dataset is designed with 20 data points per patient representing times series of 80 hours of data reported in 4-hour windows (80 = 20 × 4). There are hence 43,280 ( = 2,164 × 20) records in total.

The synthetic sepsis dataset contains 46 variables–the first 44 variables are listed in Tables 2 and 3. Similar to the synthetic hypotension dataset, the 45^th^ variable contains the synthetic patient IDs and the 46^th^ variable indicates the time steps in the time series. Table 2 presents the 35 numeric variables along with their units and descriptive statistics (*i.e*., the first, second, and third quartiles). Table 3 lists the 3 binary variables and 6 categorical variables; together with the share, in percentage, of each unique class. Unlike the synthetic hypotension dataset, the sepsis dataset contains two *quasi-identifiers*^45^, age and gender, that may be used to disclose personal information. A disclosure risk assessment is reported in the Technical Validation section.

The distributions of the variables are shown in Figs. 6 and 7. We observe that several numeric variables in the sepsis dataset are right-skewed and will likely need to be transformed before being used for downstream machine learning applications. Interested readers may consider our proposed pre-processing scheme in Section 2.1.2 of the Supplementary Materials.

There are two types of categorical variables in the sepsis dataset. GCS, for example, is a categorical variable by design–it is a clinical point-based system to measure a person’s level of consciousness. The 5 variables of SpO_2_, Temp, PTT, PT, and INR were instead originally stored as numeric variables in the MIMIC-III database^21^. These 5 variables were converted into categorical variables because their original distributions were extremely skewed and it was difficult to apply appropriate power-transformations. We decided to categorise these 5 numeric variables into deciles as reported in Table 3. Note, the 10 classes of each variable are denoted in *C*s–*e.g*., category C1 corresponds to values that lie within the 0^th^ and the 10^th^ percentile; and category C5 corresponds to values that lie within the 40^th^ and 50^th^ percentile.

### The synthetic HIV dataset

The synthetic HIV dataset is 42.6 MB and is similar to the real HIV dataset employed by Parbhoo *et al*.^20^. It contains 8,916 synthetic patients associated with time series of 60 months. The HIV data are reported in 1-month intervals; and hence there are 60 data points per patient and 534,960 ( = 8,916 × 60) records in total.

The synthetic HIV dataset contains 15 variables–the first 13 variables are listed in Table 4 together with descriptive statistics, and the two remaining variables contain the synthetic patient IDs and the month in the time series. There are 3 numeric, 5 binary, and 5 categorical variables. The descriptive statistics include the first, second, and third quartiles for the numeric variables; and the share, in percentage, of each unique class for the binary and categorical variables. This dataset also contains two quasi-identifiers, gender and ethnicity, and a disclosure risk assessment is reported in the Technical Validation section.

The distributions of the variables in the dataset are shown in Fig. 10. The numeric variables are all right-skewed and require appropriate transformation before the dataset can be used for further analysis. Interested readers may consider our proposed pre-processing scheme in Section 2.1.2 of the Supplementary Materials. Furthermore, the variables of complementary INI, complementary NNRTI, and extra PI, all have the option of *Not Applied*. This was because the medications in these categories can be substituted by medications from the other classes. A general discussion on medications for ART can be found in Section 1.3.3 of the Supplementary Materials.

## Technical Validation

This section includes a Realisticness Validation Procedure, a Disclosure Risk Assessment, and a Utility Verification. The first part demonstrates the quality of the generated synthetic datasets; the second part discusses the potential risk of an adversary learning sensitive information about a real person from the synthetic records; and the third part compares the suggested actions of RL agents trained on our Health Gym datasets against RL agents trained on the real datasets.

Based on previous work on the validation of synthetic medical data^24^, the Realisticness Validation Procedure serves to confirm that our synthetic datasets fulfil the *fidelity of individual data points* and the *fidelity of the population*. That is, we first ensure that the distributions of individual variables are sufficiently similar between the real and the synthetic datasets. We then check that all correlations between variables and trends over time in the real datasets are mirrored in the synthetic datasets.

In the Disclosure Risk Assessment, we show that while our synthetic datasets are realistic, it remains very unlikely for an adversary to learn any sensitive information about a real person using our synthetic datasets. Based on risk metrics from the *disclosure control* literature^45^, we will show that our synthetic datasets have a low *membership disclosure* risk and a low *attribute disclosure* risk. Membership disclosure refers to the scenario where an adversary is able to match a synthetic record to a real record; and attribute disclosure occurs when an adversary with partial information about a real individual is able to learn new information about that individual from a synthetic record.

While realisticness and security are crucial, it is also important to perform a Utility Verification to inspect whether machine learning algorithms trained with our synthetic datasets result in similar outcomes as those trained with the real datasets^46,47^. To this end, we will train RL agents using the synthetic and real datasets and compare their suggested actions to manage patients’ clinical conditions.

### Realisticness validation procedure

**Fig. 2:**
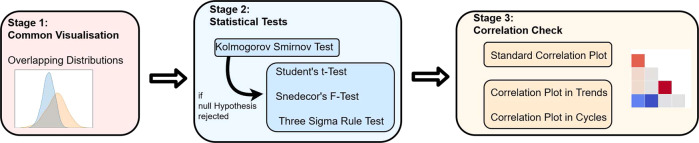
A Summary of the Realisticness Validation Procedure. The validation includes three stages. First, we perform a qualitative analysis which compares the distributions of real and synthetic variables. Next, we perform a series of statistical tests to assess whether the generated data captured the real data distribution. As a final step, we validate whether the synthetic data captured the correlations between variables over time.

Our validation procedure goes beyond prior work^48–51^ that leveraged GANs to create synthetic data and evaluated the generated data only qualitatively. We summarised the elements of our three-stage validation procedure in Fig. 2. The first two stages analyse the *static* properties of the synthetic data and assess whether the distributions and statistical moments (mean, variance) of the real and synthetic variables are sufficiently similar. Since our generated data are time series, the third stage conducts an additional set of visual comparisons to test the properties of the synthetic variables *over time*.

#### Stage one: qualitative analysis

In the first stage, we superimposed the probability density function of a synthetic numeric variable *X*_syn_ on top of the probability density function of its corresponding real variable *X*_real_. These plots were generated using *kernel density estimations* (KDE)^52^. Binary and categorical variables were compared by means of side-by-side histogram plots.

#### Stage two: statistical tests

The statistical tests in stage two include the *two-sample Kolmogorov-Smirnov test*^53–55^ (KS test), the *two independent Student’s t-test*^56,57^ (t-test), the *Snedecor’s F-test*^58,59^ (F-test), and the *three sigma rule test*^60^. The KS test compares the overall similarity between the distributions of real and synthetic variables. The t-test determines whether there are significant differences between the mean values of the real and synthetic variables; and the F-test compares their variances. Furthermore, the three sigma rule test uses the standard deviations of the real data to check whether the majority of the synthetic data was comprised within a probable range of the real variable values. Definitions and implementations of each test are reported in Sections 3.1–3.4 of the Supplementary Materials.

We organised the statistical tests in a hierarchical manner. Each synthetic variable (both numeric and categorical) was first assessed using the KS test. The KS test is the most difficult test; and when it was passed, we concluded that a synthetic variable faithfully represents its real counterpart. If a synthetic numeric variable failed the KS test, we applied the t-test, the F-test, and the three sigma rule test. If a synthetic categorical variable failed the KS test, we assessed it further using the *analysis of variance* (ANOVA) F-test and the three sigma rule test. The categorical ANOVA F-test checks the similarity in variances but over different classes. An overview of this procedure is presented in Algorithm 1 in Section 3.5 of the Supplementary Materials. No multiple testing corrections^61^ were applied, see Section 3.5 of the Supplementary Materials for more details.

#### Stage three: correlations

Our third stage of validation considers correlations between variables and between trends over time, computed using *Kendall’s rank correlation coefficients*^62^. A brief description is provided below, technical details and a discussion on alternative correlation measures^63–67^ can be found in Section 4 of the Supplementary Materials.

First, we calculated the *static correlation coefficients* for each pair of variables in the synthetic dataset *X*_syn_ and the real dataset *X*_real_ (see Section 4.2 of the Supplementary Materials). Next, the correlation coefficients for the two datasets were displayed side-by-side for visual comparison. Ideally, the synthetic dataset should mirror both the *directions* (positive or negative) and *magnitudes* of correlations between variables in the real dataset.

Though informative, the correlation between variables does not provide any information about whether temporal behaviours over time are captured by the synthetic dataset. Hence, we linearly decomposed each variable as a trend with cycle^68^. The trend indicates the general upward or downward slope of variable over time, and the cycle refers to local periodic patterns. Then, we computed and compared the *average correlation in trends* and *average correlation in cycles* (see Section 4.3 of the Supplementary Materials).

#### Validation outcomes

##### Acute hypotension

**Fig. 3:**
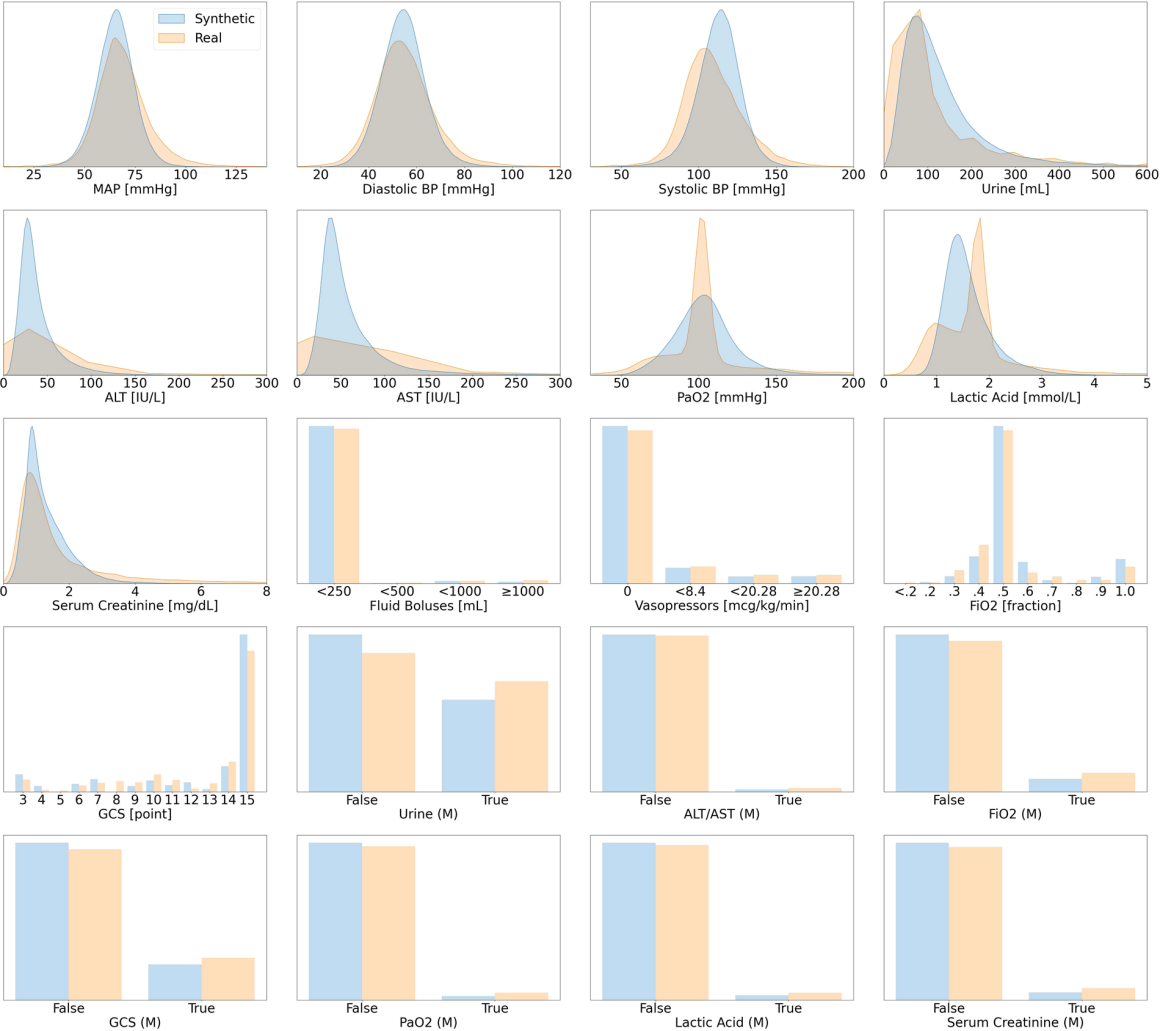
Distribution Plots for Acute Hypotension. This figure presents visual comparisons between the distributions of variables in the real and synthetic datasets for the management of acute hypotension. The distributions of real variables are plotted in orange and their synthetic counterparts are in blue.

The plots for the first stage of the validation procedure for the hypotension dataset are shown in Fig. 3. There were no major visual misalignments between the distributions of the real and synthetic datasets, and we proceeded to stage two for the statistical confirmations.

The results of stage two are shown in the hierarchically structured Table 5. The initial KS test was passed by 17 out of 20 synthetic variables. The 3 remaining variables ALT, AST, and PaO_2_ passed both the t-test and the F-test. This means that these synthetic variables do not perfectly capture the real variable distributions; however, their means and variances are still representative of their real counterparts. These observations are supported by the subplots in Fig. 3: despite some differences between the real and synthetic data, the overall behaviours are appropriately captured. Furthermore, all of these 3 variables pass the three sigma rule test. Hence we conclude that all synthetic variables capture the features of the real variable distributions. Section 3.6.1 of the Supplementary Materials contains the complete statistical results.

**Table 5:** The Stage Two Validation Results for Acute Hypotension.

**Passed the KS Test**	MAP, Diastolic BP, Systolic BP, Serum Creatinine, Fluid Boluses, Vasopressors,		
	FiO_2_, GCS, Urine, Lactic Acid, Urine (M), ALT/AST (M), FiO_2_ (M), GCS (M),		
	PaO_2_ (M), Lactic Acid (M), Serum Creatinine (M)		
**Failed the KS Test**	**Variable Name**	**t-Test Status**	**F-Test Status**
	ALT	✓	✓
	AST	✓	✓
	PaO_2_	✓	✓
**The Three Sigma Rule Test**	*passed*	ALT, AST, PaO_2_	
	*failed*	—	

This table summarises the results of the statistical tests. The tests were conducted in the order of the KS-test, then the t-test and F-test, and finally the three sigma rule test. Only those variables that failed the KS-test underwent the additional tests. 17 of the 20 variables in the synthetic hypotension dataset passed the KS-test and did not have different distributions from their real counterparts. The remaining 3 variables passed all additional tests. Therefore, all variables of the synthetic hypotension dataset were realistic. This table should be compared with Fig. 3 and Table 1.

**Fig. 4:**
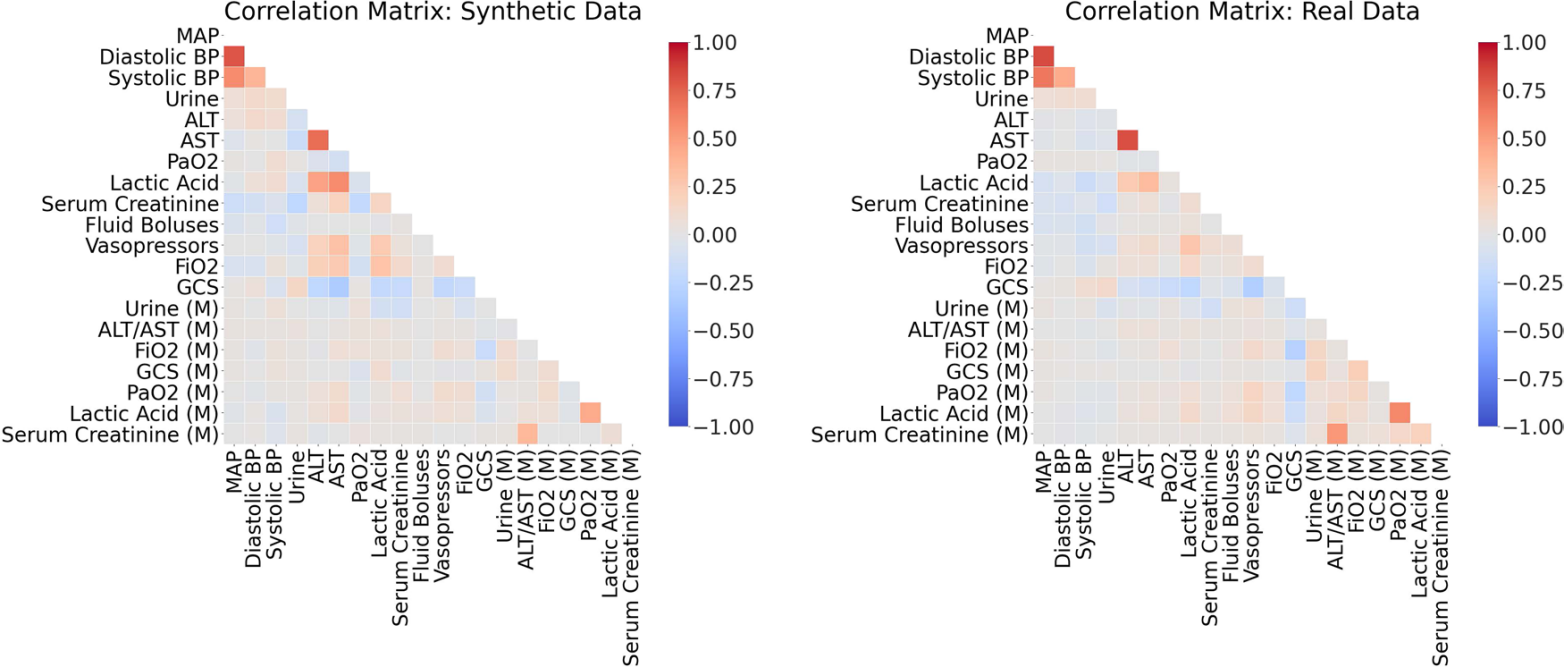
The Static Correlations for Acute Hypotension. This is a side-by-side comparison of the static correlations in the synthetic dataset and the real dataset. It illustrates the correlation between all pairs of variables, across all patients and timepoints. Positive correlations are coloured in red and negative correlations are in blue. The magnitudes of the correlations are indicated by their colour saturation.

**Fig. 5:**
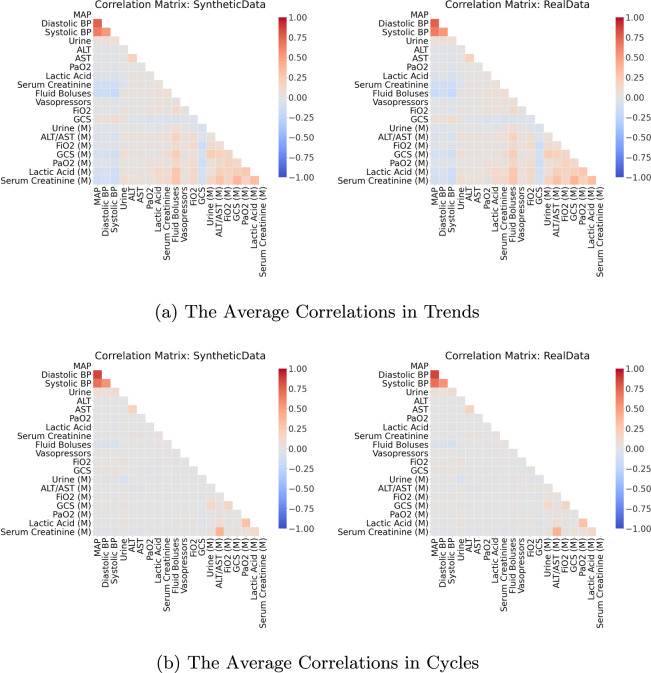
The Dynamic Correlations for Acute Hypotension. This is a side-by-side comparison of the dynamic correlations in the synthetic dataset and the real dataset. Unlike the static correlations of Fig. 4, all variables are treated as time series and are linearly decomposed into trends and cycles. They illustrate the average correlation between all pairs of variables for each individual patient. Refer to Fig. 4 for the details on the colour scheme.

After confirming the realisticness of the individual synthetic variables, we assessed the relations between variables and their longitudinal properties in the third validation stage. We illustrate the static correlations in Fig. 4 and the dynamic correlations in Fig. 5. There is no major misalignment between the static correlations of the real and synthetic datasets. However, the synthetic dataset slightly increases the magnitudes of some correlations. For instance, there is a stronger positive correlation between lactic acid and AST in the synthetic dataset than in the real counterpart. Likewise, there is a stronger negative correlation between the synthetic variables of serum creatinine and urine than in the real pair of variables. Nonetheless, the generated data is still highly reliable. In Fig. 5, the dynamic correlations (both in trends and in cycles) of the decomposed synthetic time series strongly resemble their real counterparts. This indicates that the characteristics of the generated time series variables are realistic. All three stages of our validation confirmed that the synthetic hypotension dataset adequately characterises the properties of the real dataset.

##### Sepsis

**Fig. 6:**
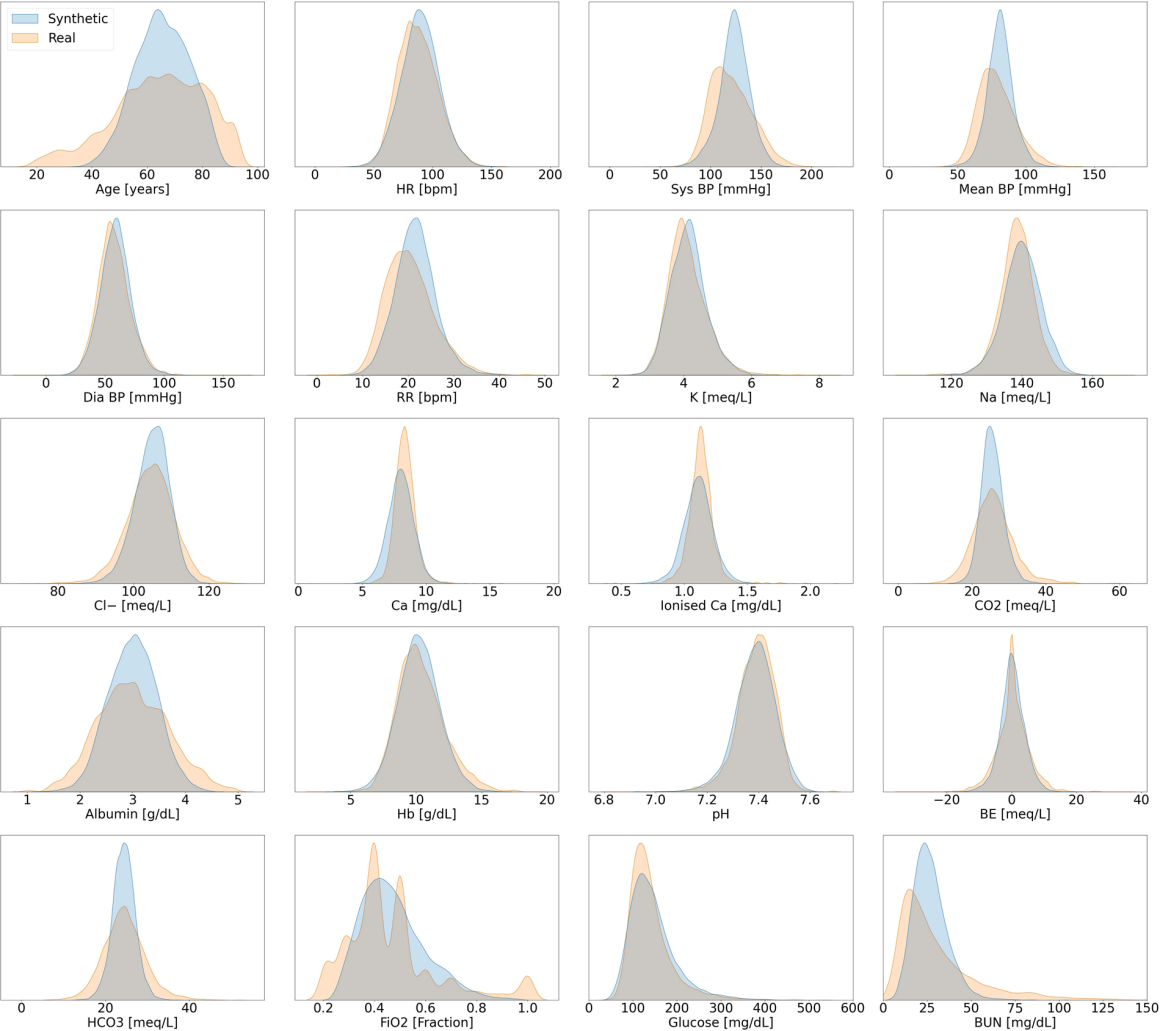
Distribution Plots for Sepsis. This figure presents the visual comparisons between the distributions of variables in the real and synthetic datasets for the management of sepsis. It follows the format of Fig. 3 and is continued in Fig. 7.

**Fig. 7:**
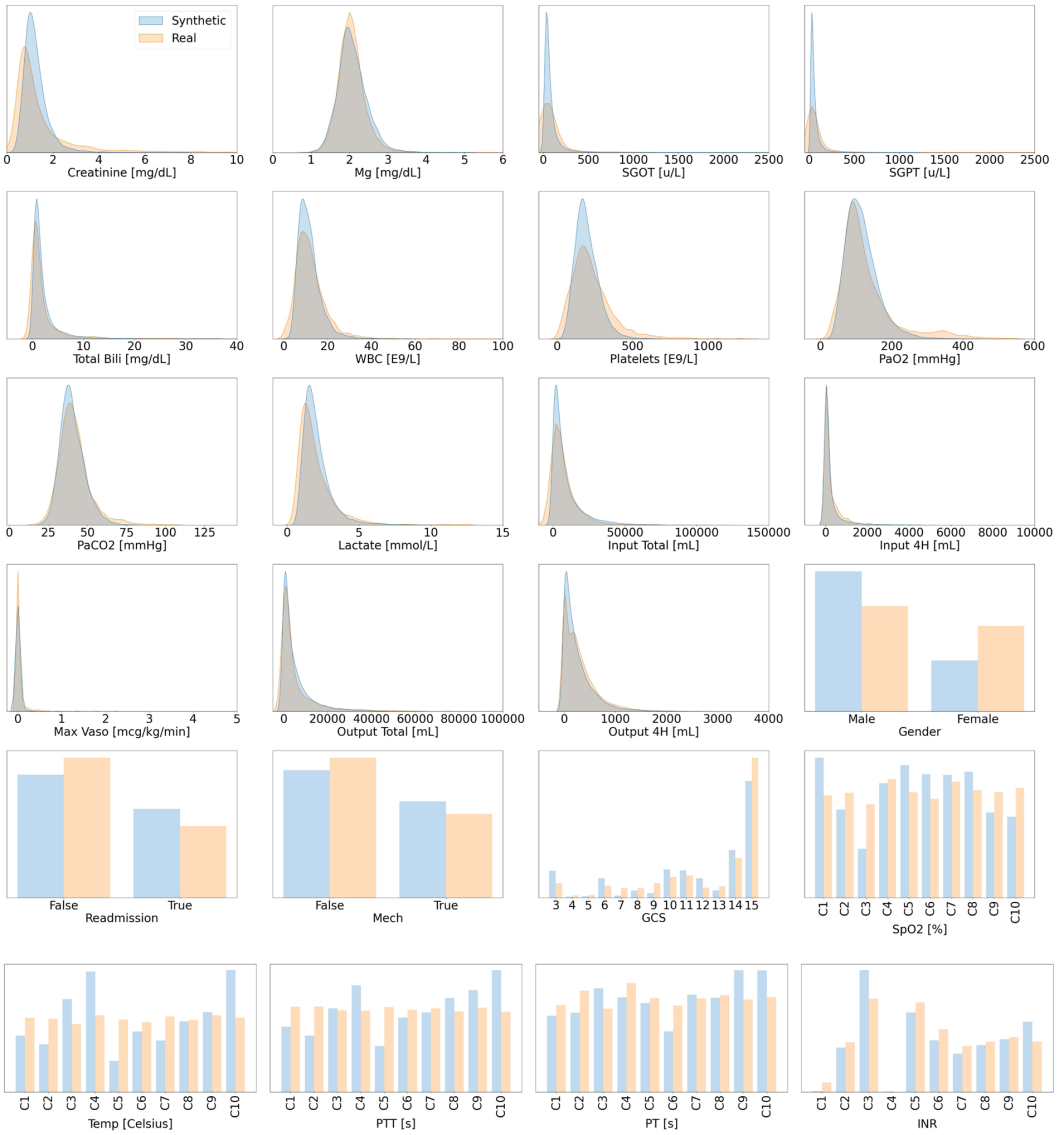
Distribution Plots for Sepsis. This figure serves as a continuation to Fig. 6. All variables are strictly positive but may appear to include negative values as an artefact of using kernel density estimation for plotting the distributions (see: https://stats.stackexchange.com/questions/109549/negative-density-for-non-negative-variables).

For the synthetic sepsis dataset, we observe in Figs. 6 and 7 that all synthetic variable distributions were very similar to their real counterparts. In stage two, we found that 43 out of 44 variables passed the KS test and therefore almost all synthetic variables mirrored the distributions of their real counterparts. The only variable that failed the KS test was Max Vaso. Since the variable also failed the following F-test, this was because of differences in the variance (see Table 6). As shown in Fig. 7, Max Vaso is highly skewed. As discussed in the Data Records section, we could have transformed Max Vaso into a categorical variable but decided to keep it as numeric because the closely related variables of Input Total, Input 4H,Output Total, and Output 4H are all numeric. These 5 variables collectively describe the input/output measurements of the patients and should therefore share one common data type. Nonetheless, Max Vaso did pass the three sigma rule test. This indicated that while there was a difference in variance for the synthetic Max Vaso variable, the generated data were within the plausible range of the real data. The complete results of all statistical tests are reported in Section 3.6.2 of the Supplementary Materials.

**Table 6:** The Stage Two Validation Results for Sepsis.

**Passed the KS Test**	Age, HR, Systolic BP, Mean BP, Diastolic BP, RR, K^+^, Na^+^, Cl^−^, Ca,		
	Ionised Ca^++^, CO_2_, Albumin, HB, pH, BE, HCO_3_, FiO_2_, Glucose, BUN, Creatinine,		
	Mg^++^, SGOT, SGPT, Total Bili, WBC, Platelets, PaO_2_, PaCO_2_, Lactate,		
	Input Total, Input 4H,Output Total, Output 4H,Gender, Readmission, Mech, GCS,		
	SpO_2_, Temp, PTT, PT, INR		
**Failed the KS Test**	**Variable Name**	**t-Test Status**	**F-Test Status**
	Max Vaso	✓	×
**The Three Sigma Rule Tes**t	*passed*	Max Vaso	
	*failed*	—	

This table presents the statistical results for the synthetic sepsis dataset. It follows the format of Table 5; and should be compared with Figs. 6 and 7 and Tables 2 and 3.

**Fig. 8:**
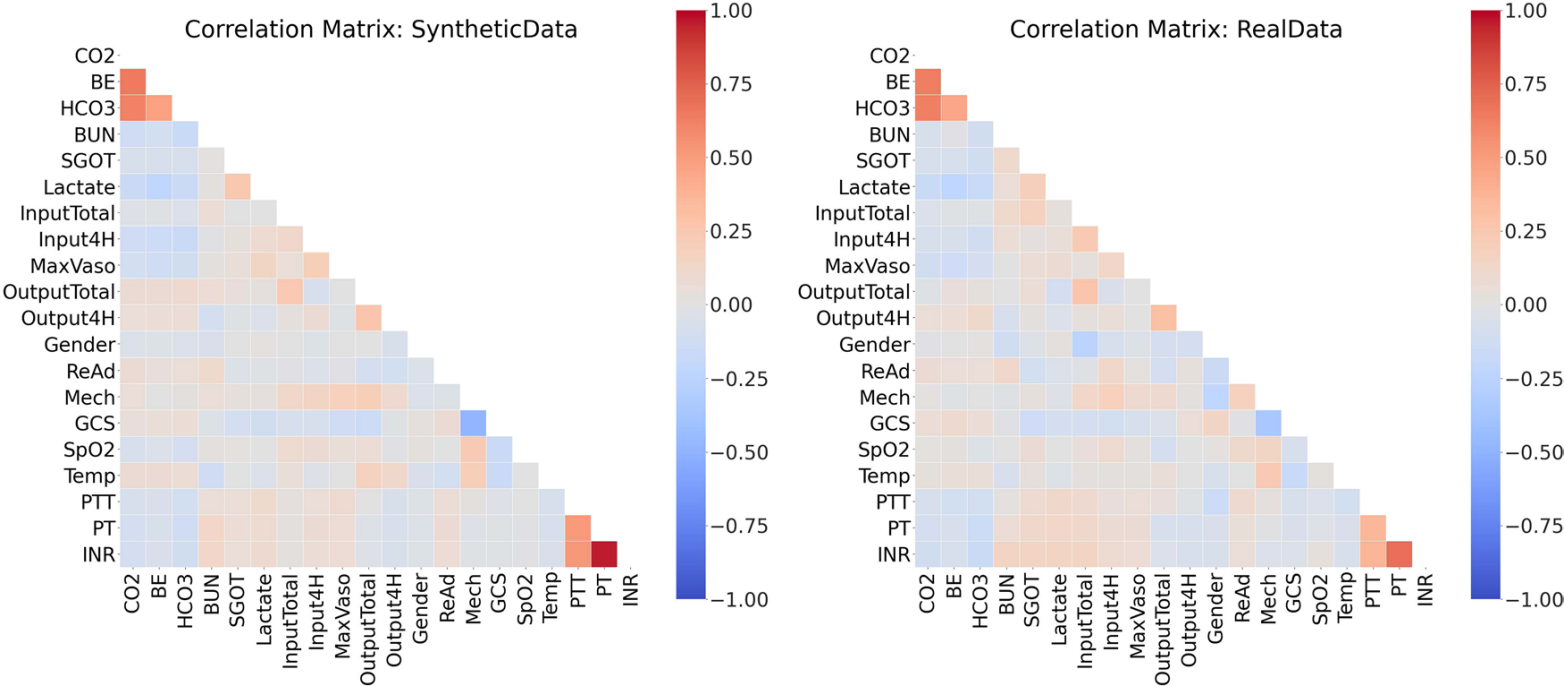
The Top 20 Static Correlations for Sepsis. This figure presents the static correlations between a subset of the variables in the sepsis dataset. It follows the format of Fig. 4; and the full correlation plots for all variables can be found in Section 5 of the Supplementary Materials.

**Fig. 9:**
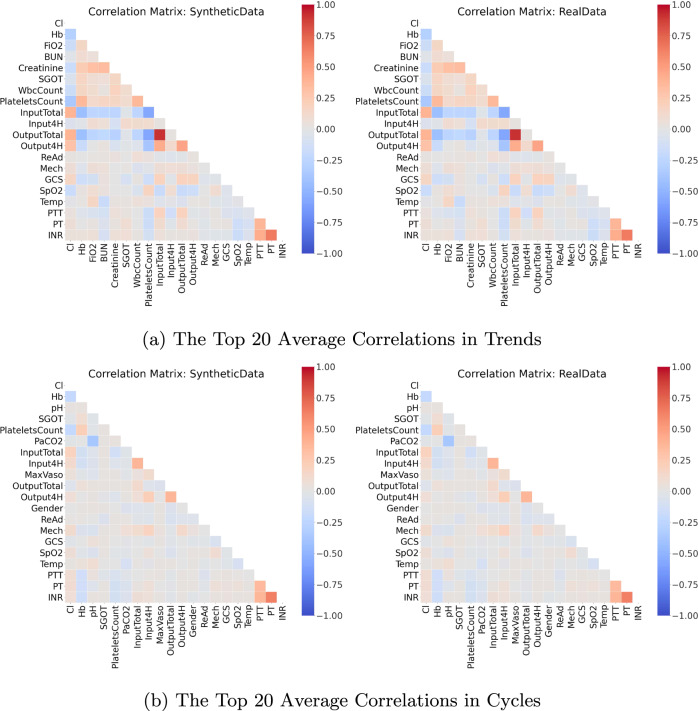
The Top 20 Dynamic Correlations for Sepsis. This figure presents the dynamic correlations between a subset of the variables in the sepsis dataset. It follows the format of Fig. 5; the full correlation plots for all variables can be found in Section 5 of the Supplementary Materials.

The correlations computed in stage three of the validation procedure are visualised in Figs. 8 and 9 for a subset of the 20 variables that were associated with the strongest correlations. Both the static and the dynamic correlations were very similar between the real and synthetic dataset. Interested readers may find illustrations of the full correlation matrices for all variable pairs in Section 5 of the Supplementary Materials.

##### HIV

**Fig. 10:**
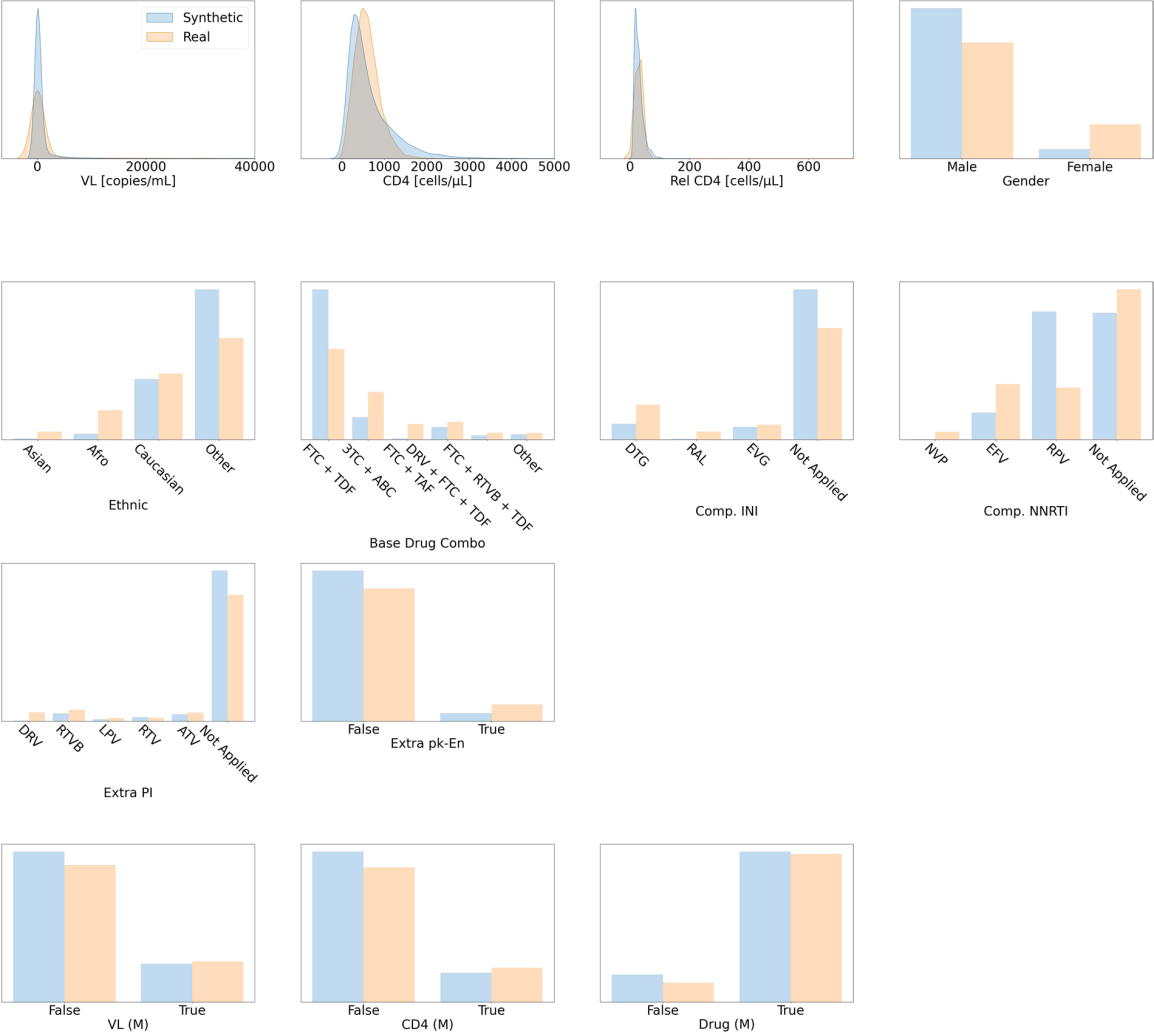
Distribution Plots for HIV. This figure follows the format of Fig. 3 and presents the visual comparisons between the distributions of variables in the real and synthetic datasets for the optimisation of antiretroviral therapy for HIV.

Qualitative comparisons between the distributions of the real and synthetic HIV datasets are shown in Fig. 10, indicating high similarity. As presented in Table 7, 12 out of 13 variables passed the KS test, suggesting that the distributions of most synthetic variables matched their real counterparts. The only variable that failed the KS test is VL. VL also failed the F-test, similarly to Max Vaso in the synthetic sepsis dataset. However, VL still passed the three sigma rule test and therefore we can conclude that all variables in the synthetic dataset are highly realistic. Section 3.6.3 of the Supplementary Materials contains the complete statistical results.

**Table 7:** The Stage Two Validation Results for HIV.

**Passed the KS Test**	CD4, Rel CD4, Gender, Ethnic, Base Drug Combo, Comp. INI, Comp. NNRTI, Extra PI		
	Extra pk-En, VL (M), CD4 (M), Drug (M)		
**Failed the KS Test**	**Variable Name**	**t-Test Status**	**F-Test Status**
	VL	✓	×
**The Three Sigma Rule Test**	*passed*	VL	
	*failed*	—	

This table presents the statistical results for the synthetic HIV dataset. It follows the format of Table 5; and should be compared with Fig. 10 and Table 4.

**Fig. 11:**
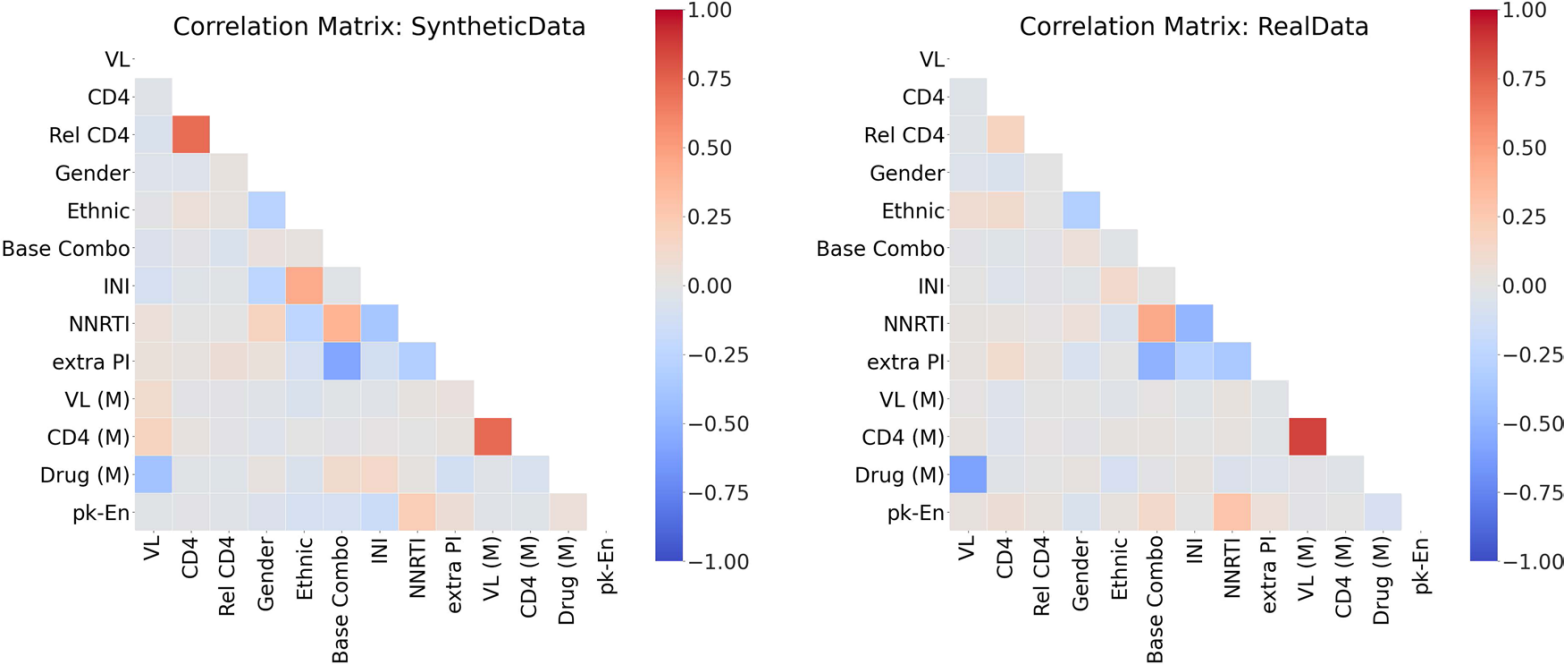
The Static Correlations for HIV. This figure presents the static correlations between the variables in the HIV dataset. It follows the format of Fig. 4.

**Fig. 12:**
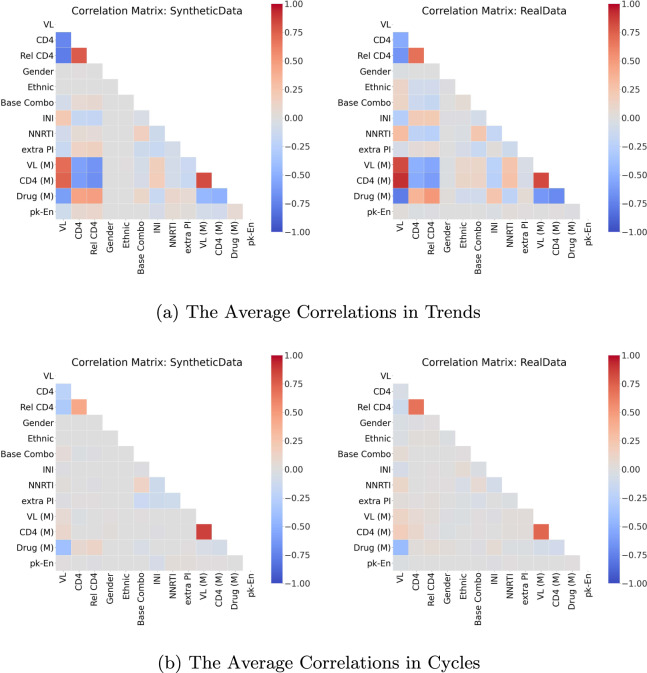
The Dynamic Correlations for HIV. This figure presents the dynamic correlations between the variables in the HIV dataset. It follows the format of Fig. 5.

For stage three, we present the correlations in Figs. 11 and 12. Both the static and dynamic correlations reflect that the synthetic dataset captures the relations among the variables in the real dataset.

### Disclosure risk assessment

We performed two tests to evaluate the likelihood of an attacker learning sensitive information about an individual from the generated synthetic datasets.

#### Euclidean distances

The first test was to ensure that no records in the real datasets were simply copied by the GAN to the synthetic datasets. We computed the Euclidean distances (*L*_2_ norms) between records in the real dataset *X*_real_ and records in the synthetic dataset *X*_syn_. We verified that all distances were greater than zero, *i.e*., that no records in the synthetic datasets perfectly matched any records in the real datasets.

#### Disclosure risks

The second test concerned the *disclosure risks* associated with the public distribution of the synthetic datasets. Despite being anonymised, the data may contain sets of variables (*e.g*., age and gender) which, in combination, may be used by an adversary to uniquely identify a person (*e.g*., via linking the data with voter registration lists^69^). Variables which in combination constitute personally identifying information are known as *quasi-identifiers*. Individuals with the same combination of quasi-identifiers (*e.g*., all 21-year-old males) form an *equivalent class*.

El Emam *et al*.^45,70^ introduced two types of disclosure risks based on the concepts of quasi-identifiers and equivalent classes. Depending on the direction of attack^71^, an adversary may attempt to learn new information about a person either by finding out whether an individual in the population (or database) is also included in the real or synthetic dataset (*population-to-sample attack*) or by linking an individual in the real or synthetic dataset back to the original database (*sample-to-population attack*).

Whereas El Emam *et al*. assumed that the real dataset was sampled randomly from the database, in this study the real datasets were constructed using publicly accessible inclusion and exclusion criteria (*i.e*., information documented in Section 1 of the Supplementary Materials). Therefore, we assumed that the adversary had access not only to the database (*e.g*., MIMIC-III or EuResist) but also to the real dataset. One of the likely reasons to conduct a population-to-sample attack is to determine whether an individual has a specific condition or illness that led to their inclusion in the dataset. However, when the inclusion criteria are known, population-to-sample attacks become less relevant than sample-to-population attacks, which may be used to learn additional sensitive information about an individual in the synthetic dataset.

The risk of a successful synthetic-to-real attack (*i.e*., the chance of matching a random individual in the synthetic dataset to an individual in the real dataset) can be computed as

4 $$\frac{1}{S}\mathop{\sum }\limits_{s=1}^{S}\left(\frac{1}{{F}_{s}}\times {I}_{s}\right)$$

where *S* is the number of records in the synthetic dataset, *F*_*s*_ is the size of the equivalent class in the real dataset that shares the same combination of quasi-identifiers as a specific record *s* in the synthetic sample, and *I*_*s*_ is a binary indicator variable equal to one if at least one real record matches the synthetic records *s*. Interested readers can find more details on El Emam *et al*. ‘s metric in Section 6.1 of the Supplementary Materials.

To assess the risk of information disclosure, we adopted the acceptable risk threshold value of 9% proposed by the European Medicines Agency^72^ and Health Canada^73^ for the public release of clinical trial data. In their work, El Emam *et al*.^45^ had instead used 5%. A discussion on alternative risk metrics^74–77^ can be found in Section 6.2 of the Supplementary Materials.

#### Risk assessment outcomes

##### Acute hypotension

As shown in Table 1, all variables in the synthetic hypotension dataset are associated with the patient’s bio-physiological states and do not contain any quasi-identifiers or sensitive information. For this reason, for this dataset we tested the Euclidean distances but not the disclosure risk.

No records in the synthetic dataset completely matched any records in the real hypotension dataset. The smallest distance between any synthetic record and any real record was 49.06 (>0). Therefore no record was leaked into the synthetic dataset.

##### Sepsis

Through the Euclidean distance test, we found that no record in the synthetic sepsis dataset was identical to any record in the real sepsis dataset. The smallest distance between real and synthetic records was 328.78 ( > 0), which was considerably larger than the smallest distance for the hypotension data (49.06). This is likely due to the larger number of variables in the sepsis dataset (44 vs 20 in the hypotension dataset, compare Table 1 with Tables 2 and 3). Furthermore, many sepsis variables are highly skewed (see Fig. 7) hence exaggerating any value differences. Importantly, for both the synthetic hypotension dataset and the synthetic sepsis dataset, the minimal Euclidean distance is greater than zero.

The sepsis variables include the quasi-identifiers age and gender. Therefore, age (rounded down to the closest year) and gender were combined to create different equivalence classes *e.g*., all 21-year-old males and all 22-year-old males were in separate equivalence classes. The risk of a successful synthetic-to-real attack was estimated to be 0.80%. This risk is much lower than the suggested threshold of 9%^72,73^, indicating that there is minimal risk of sensitive information disclosure associated with the release of the synthetic sepsis dataset.

##### HIV

The minimal Euclidean distance between any pair of real and synthetic HIV records was 0.11 (>0); and hence no data leaked from the real dataset into the synthetic dataset. This value is relatively low for two reasons: 1) there are very few variables in the HIV dataset; 2) most variables are either binary or categorical. The reasons that inflate the Euclidean distance for sepsis are thus the same reasons that deflate the Euclidean distance for HIV.

The HIV variables include the quasi-identifiers gender and ethnicity. These two variables were combined to create different equivalence classes (*e.g*., male Asian and female Caucasian). The risk of a successful synthetic-to-real attack was estimated to be 0.041%. This risk is again much lower than the typical 9% threshold, indicating that also the synthetic HIV dataset can be released with minimal risk of sensitive information disclosure.

### Utility verification

We employed synthetic and real datasets to train RL agents to verify the utility of the synthetic datasets. A high level of utility is achieved in the synthetic datasets when an RL agent trained by the real and synthetic datasets suggest similar actions when presented with patients’ clinical conditions.

This verification involves splitting each dataset into two subsets–a subset of observational variables *D*_*O*_ and a subset of action variables *D*_*A*_. Collectively, the variables in *D*_*O*_ describe the clinical condition of a patient, whereas *D*_*A*_ enables us to define the actions that could be taken by an RL agent. Following the work of Liu *et al*.^78^, we applied cross decomposition^79^ to reduce the dimensionality of *D*_*O*_ to 5 variables. We then performed K-Means clustering^80^ with 100 clusters, and labelled each data point of *D*_*O*_ using their associated clusters to define the state space ${\mathfrak{S}}$. In addition, the action space *A* was spanned by the combinations of unique values of the action variables.

Subsequently, we followed published reward functions^19,20,81^ to determine optimal clinical actions that an RL agent should select from *A* given patient state ${\mathfrak{S}}$. Our RL method of choice was batch-constrained Q-learning^82^, but many alternative methods for offline RL have recently been developed^83^. Optimal policies were derived after 100 iterations with step size 0.01. Interested readers may find more details of the process documented in Section 7 of the Supplementary Materials.

#### Acute hypotension

**Fig. 13:**
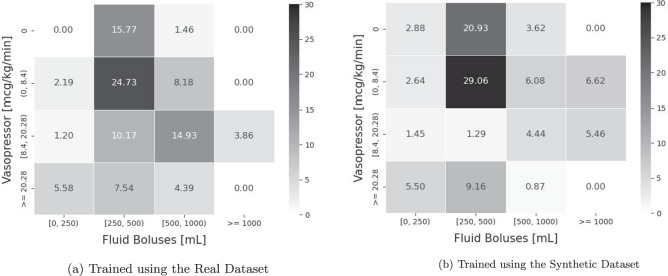
The Relative Frequencies of Actions Taken by Trained RL Agents for Managing Acute Hypotension. The action space for managing acute hypotension is described by the different levels of Fluid Boluses and Vasopressor. There are 16 ( = 4 × 4) actions in total and each unique action is represented by a coloured tile in the heatmap. The number in each tile represents the relative frequency of the RL agent taking a specific action, in proportion (in %) of all actions. The numbers in all tiles of a heatmap sum up to 100; and the deeper the colour of the tile, the more often/likely an action is taken by the RL agent. In subplot (**a**), we present the relative frequencies of actions taken by an RL agent trained using the real dataset; and in subplot (**b**), we show its counterpart trained using the synthetic dataset.

Fluid Boluses and Vasopressors were used to define the action space *A*, resulting in 16 ( = 4 × 4) unique actions; whereas *D*_*O*_ comprised the remaining 18 variables (see Table 1). After defining the state space and action space, we updated the RL policy using the reward function defined in Gottesman *et al*.^12^. Details of Gottesman *et al*.’s reward function can be found in Section 7.1 of the Supplementary Materials; and the action space of the RL agents are presented in Fig. 13.

The heatmaps in Fig. 13 illustrate the relative frequencies of actions taken by the trained RL agents. Each tile represents a unique action, and the number on the tile represents the frequency of that action, as a proportion of all actions. Furthermore, the darker the colour of a tile, the more likely an RL agent is to suggest the corresponding action. In subplot (a) we present the actions taken by an RL agent trained using the real dataset; whereas in subplot (b) we show the actions taken by its counterpart trained using the synthetic dataset. The heatmap in subplot (b) largely matches the one in subplot (a), indicating that an RL agent trained using the synthetic dataset suggested similar actions to an RL agent trained using the real dataset. The utility of the synthetic acute hypotension dataset is hence high.

#### Sepsis

**Fig. 14:**
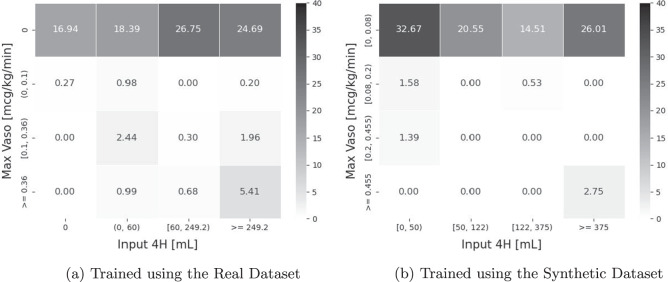
The Relative Frequencies of Actions Taken by Trained RL Agents for Managing Sepsis. The format of this figure follows that of Fig. 13. The action space for managing sepsis is described by the different levels of intravenous fluid in the 4-hourly window (Input 4H) and maximum vasopressor issued (Max Vaso); and there are 16 ( = 4 × 4) actions in total.

The total amount of intravenous fluids in the 4-hourly window (Input 4H) and the maximum dose of vasopressors in the same time frame (Max Vaso) were used to define the action space *A*, resulting in 16 ( = 4 × 4) unique actions; *D*_*O*_ comprised the remaining 42 variables (see Tables 2 and 3). We updated the RL policy using the reward function defined in Raghu *et al*.^81^. Details of Raghu *et al*.’s reward function can be found in Section 7.2 of the Supplementary Materials; and the action space of the RL agents are presented in Fig. 14.

The heatmap in Fig. 14(b) matches that in Fig. 14(a), indicating that similar actions were suggested by the RL agents trained on the real and synthetic datasets. The utility of the synthetic sepsis dataset is hence high.

#### HIV

**Fig. 15:**
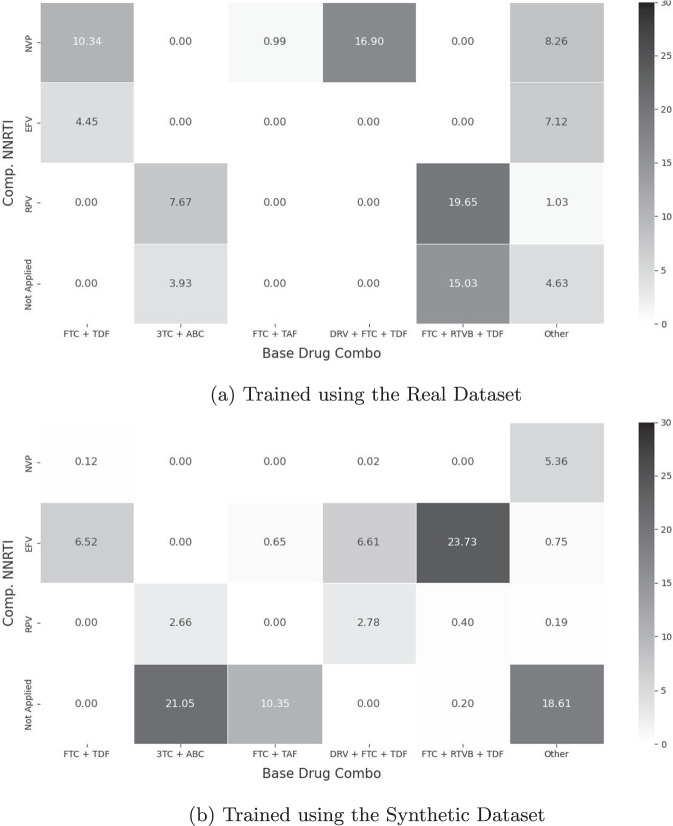
The Relative Frequencies of Actions Taken by Trained RL Agents for Managing HIV. The format of this figure follows that of Fig. 13. The action space for managing HIV is described by the different levels of base drug combinations (Base Drug Combo) and complementary NNRTI (Comp. NNRTI); and there are 24 ( = 6 × 4) actions in total.

The base drug combinations (Base Drug Combo) and complementary NNRTI (Comp. NNRTI) were used to define the action space *A*, resulting in 24 ( = 6 × 4) unique actions; *D*_*O*_ comprised the remaining 11 variables (see Table 4). We updated the RL policy using the reward function adapted from Parbhoo *et al*.^20^. Details of Parbhoo *et al*.’s reward function can be found in Section 7.3 of the Supplementary Materials; and the action space of the RL agents are presented in Fig. 15.

The heatmaps in Fig. 15 show that there are some differences in the actions suggested by an RL agent trained on the real HIV dataset and an RL agent trained on the synthetic HIV dataset. The two largest differences were

(Base Drug Combo, Comp. NNRTI) = (FTC + RTVB + TDF, EFV),

suggested 0.00% of times by an agent trained on the real data and 23.73% of times by an agent trained on the synthetic data; and

(Base Drug Combo, Comp. NNRTI) = (DRV + FTC + TDF, NVP),

suggested 16.90% of times by an agent trained on the real data and 0.02% of times by an agent trained on the synthetic data.

While the direct cause of the mis-alignments remains uncertain, these differences are likely caused by the highly sparse feature representation space of the HIV dataset. As mentioned in the Methods section, we selected the top 50 medication combinations spanning 21 medications from the EuResist database. However, the medication combinations were then deconstructed into five categorical variables–Base Drug Combo (with 6 classes), Comp. INI (with 4 classes), Comp. NNRTI (with 4 classes), Extra PI (with 6 classes), and Extra pk-En (with 2 classes)–hence resulting in a total of 1,152 ( = 6 × 4 × 4 × 6 × 2) potential synthetic medication combinations. It is thus difficult to capture the 50 medication combinations that were observed in the real dataset out of the 1,152 candidates and hence introducing extremely high sparsity.

A previous study also observed the difficulty of training RL agents for optimising ART in HIV patients^20^ and recent offline RL methods that encourage the selection of actions observed in the data may be useful for this task^83^. It is also possible that the dataset does not include important confounders (*e.g*., treatment adherence, HIV genome) which may be necessary to map a patient’s clinical history to an optimal combination of medications.

Although the utility of the synthetic HIV dataset appears to be lower than the synthetic acute hypotention and synthetic sepsis datasets, its variables are highly realistic (see the Validation Outcomes subsection) and we believe that it is still a useful dataset to develop and prototype RL algorithms. As shown in Fig. 15, many actions avoided by an RL agent trained with the real dataset were also avoided by the RL agent trained with the synthetic dataset.

## Usage Notes

### Discussion

This paper introduces the Health Gym project with three highly realistic synthetic datasets generated with GANs. We are aware of only one other GAN-based model which attempted to create both numeric and non-numeric variables at the same time. Li *et al*.^84^ proposed a twin-encoder approach to separately embed numeric and non-numeric data, which required adding a matching loss for training the generative model. In contrast, the GAN model proposed in this study does not require the extra architectural constraint; instead, our binary and categorical variables are mapped to continuous vectors through the use of soft-embeddings.

We based our work on published inclusion and exclusion criteria^10,19,20^ and thus our Health Gym datasets include the variables that can be used to define the observations, actions, and rewards for training RL agents for the management of clinical conditions. While our Health Gym datasets were primarily prepared for RL, our datasets also contain sufficient information for developing supervised or unsupervised machine learning models^85^.

### Broader impact & future work

The authors of this manuscript would like to emphasise that, while our synthetic datasets are realistic, the generated synthetic datasets should not be regarded as replacements for the real datasets. Furthermore, we will continue to incorporate synthetic data into a *Controlled Data Processing Workflow*^46,86^ for external researchers to train models, develop scripts, and then to compare and test them with real data.

In this paper, we used batch-constrained Q-learning to evaluate whether our synthetic datasets could be used to train RL agents that behave similarly to those trained using the real datasets. We intend to leverage recent advancements in offline RL algorithms as part of future work to further evaluate the utility of the Health Gym datasets.

Whereas optimal policies determined using the synthetic hypotension and sepsis datasets were very similar to optimal policies determined using the real datasets, considerable differences were observed for the HIV data. This may indicate that the current Health Gym GAN requires further fine-tuning to fully capture the complexity of a dataset consisting of multiple inter-connected categorical variables. For instance, the recurrent components of the Health Gym GAN could potentially benefit from existing work on network simplification^87^.

Diffusion models are an alternative approach for generating synthetic data, and they have recently achieved results comparable to state-of-the-art GANs^88^. In future work, we plan to explore the use of diffusion models for improving the sample diversity and robustness of the training process of our generative models. Furthermore, the generated data could be made more realistic, and the generative process more explainable, by incorporating causal layers^89^.

## Supplementary information

Supplementary Figure 1

Supplementary Figure 2

Supplementary Figure 3

Supplementary Materials for the Health Gym Paper

## Data Availability

The software code related to the Health Gym project is publicly available at https://github.com/Nic5472K/ScientificData2021_HealthGym. Our code is mainly written in Python^90^ using the PyTorch^91^ package for deep learning. In order to replicate our results, users will need access to the MIMIC-III^21,22^ and EuResist^23^ databases. MIMIC-III is a restricted-access resource; and users must complete the data use agreements on PhysioNet (see: https://physionet.org/content/mimiciii/1.4/). The EuResist Integrated DataBase (EIDB) can be accessed for scientific studies once a proposal for analysis has been approved by EuResist’s Scientific Board (see: http://engine.euresist.org/database/). Additional code for the data pre-processing for acute hypotension and sepsis can be found in the repository of Komorowski *et al*.^10^ at https://gitlab.doc.ic.ac.uk/AIClinician/AIClinician/-/tree/master/. This includes a combination of code in SQL^92^, Matlab^93^, Python, and their extension packages^94–97^. For more details, see explanations and usages in the Supplementary Materials.
