# Supplementary figures and images for: The Health Gym: synthetic health-related datasets for the development of reinforcement learning algorithms
Source: Sci Data. 2022 Nov 11;9:693. doi: 10.1038/s41597-022-01784-7 (PMC9652426; doi:10.1038/s41597-022-01784-7)

Correlation Matrix: SyntheticData

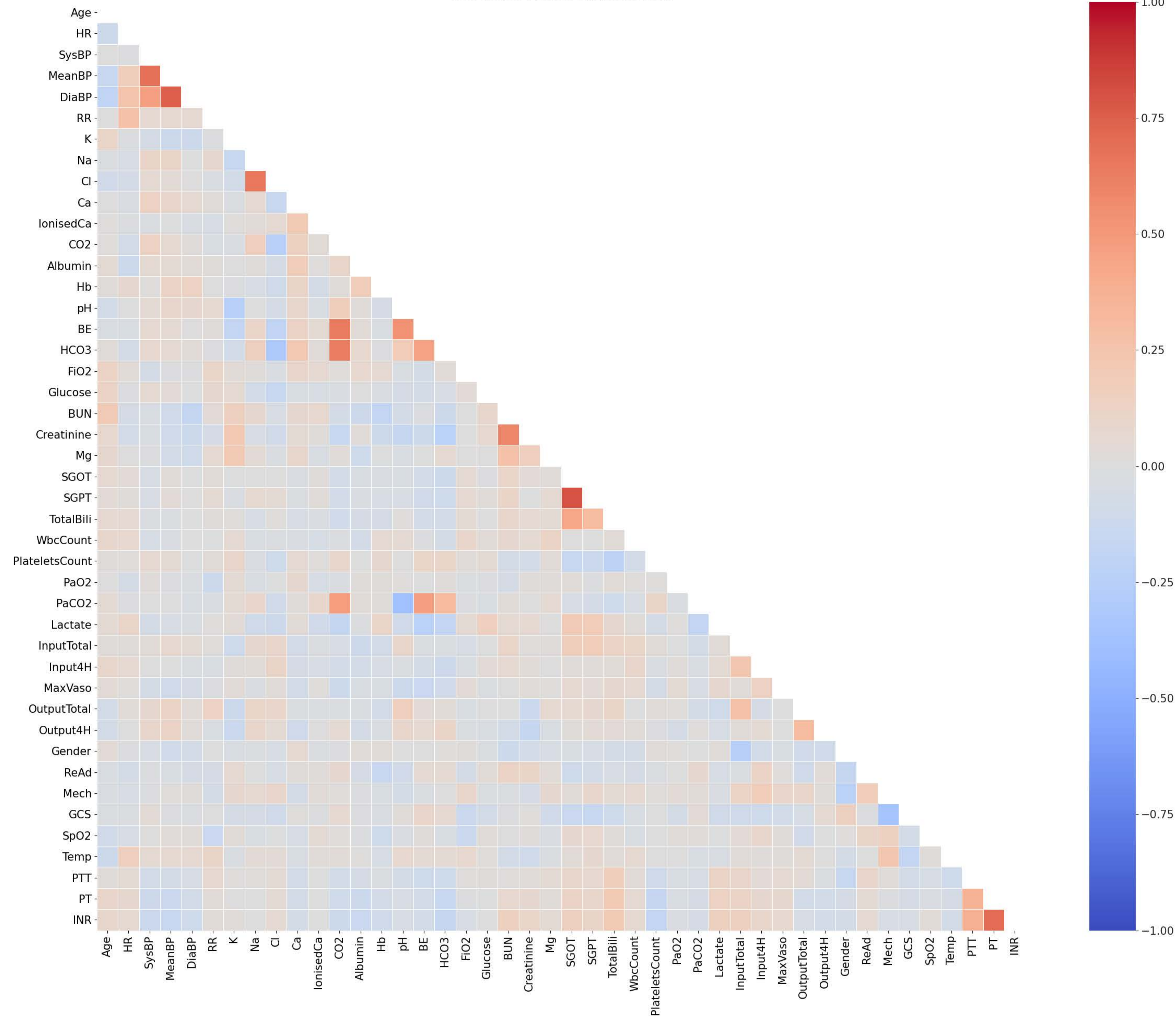

Correlation Matrix: RealData

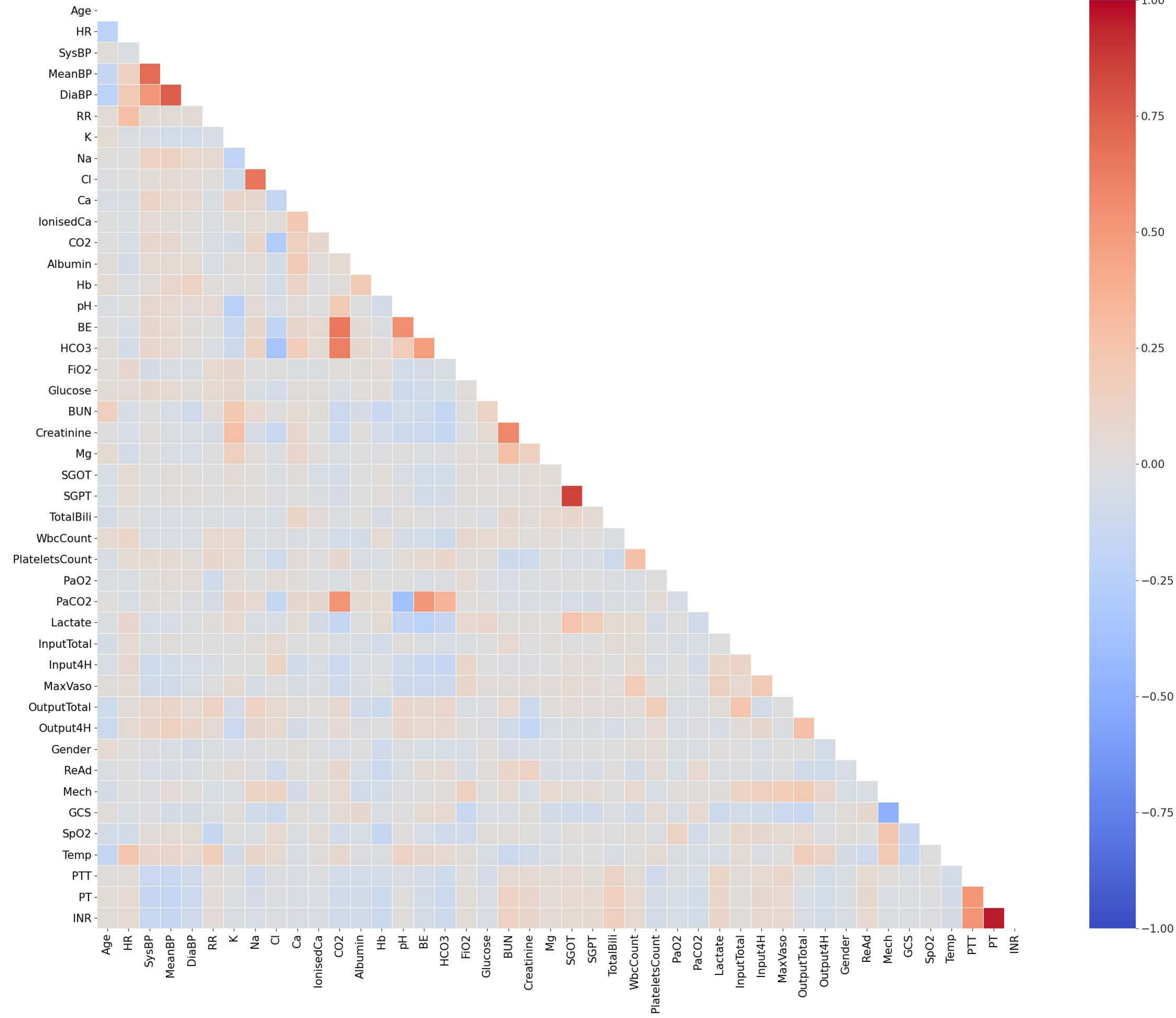

Supplement: Supplementary file 1 — Supplementary Figure 1 [file 41597_2022_1784_MOESM1_ESM.pdf]

Correlation Matrix: SyntheticData

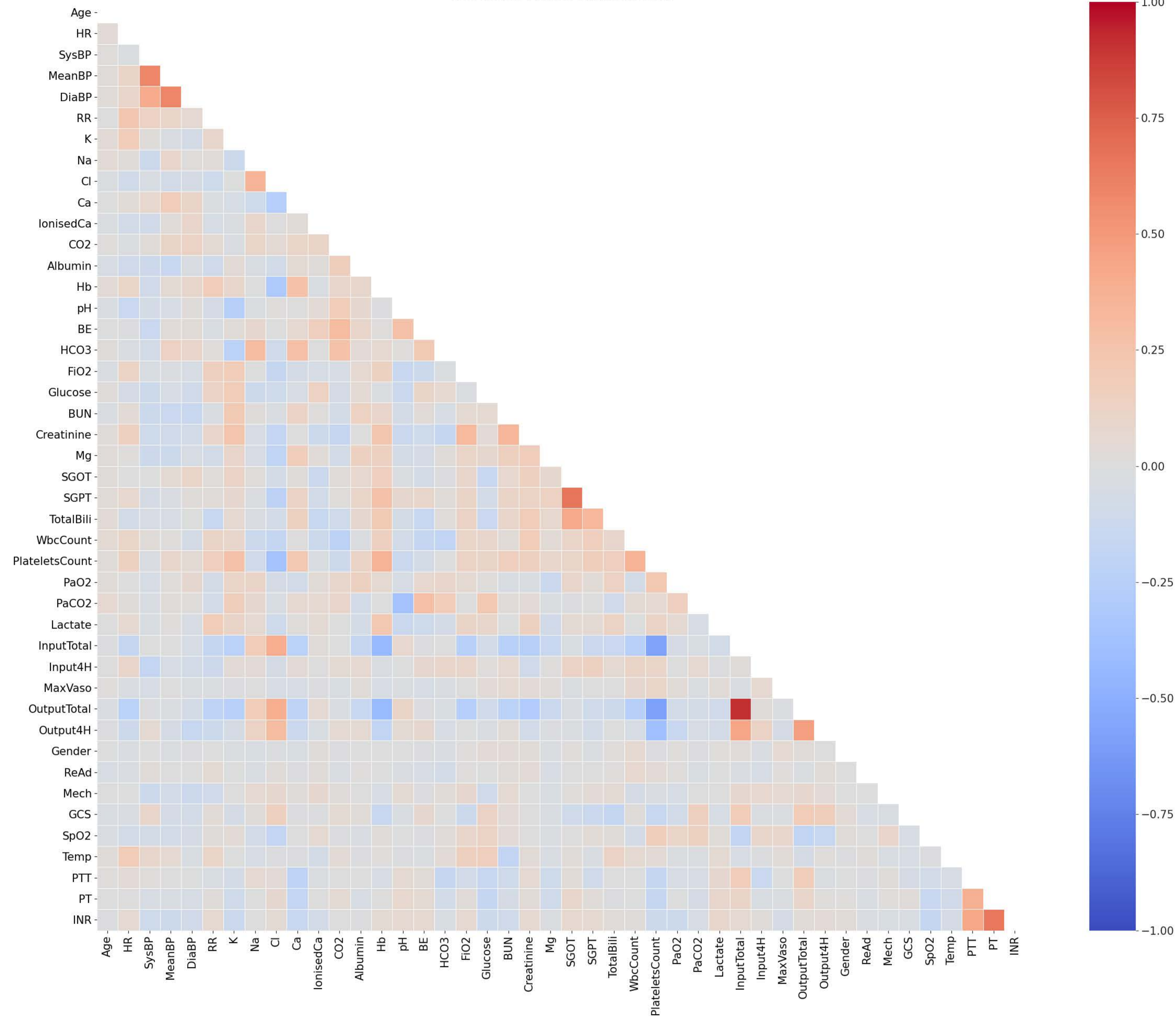

Correlation Matrix: RealData

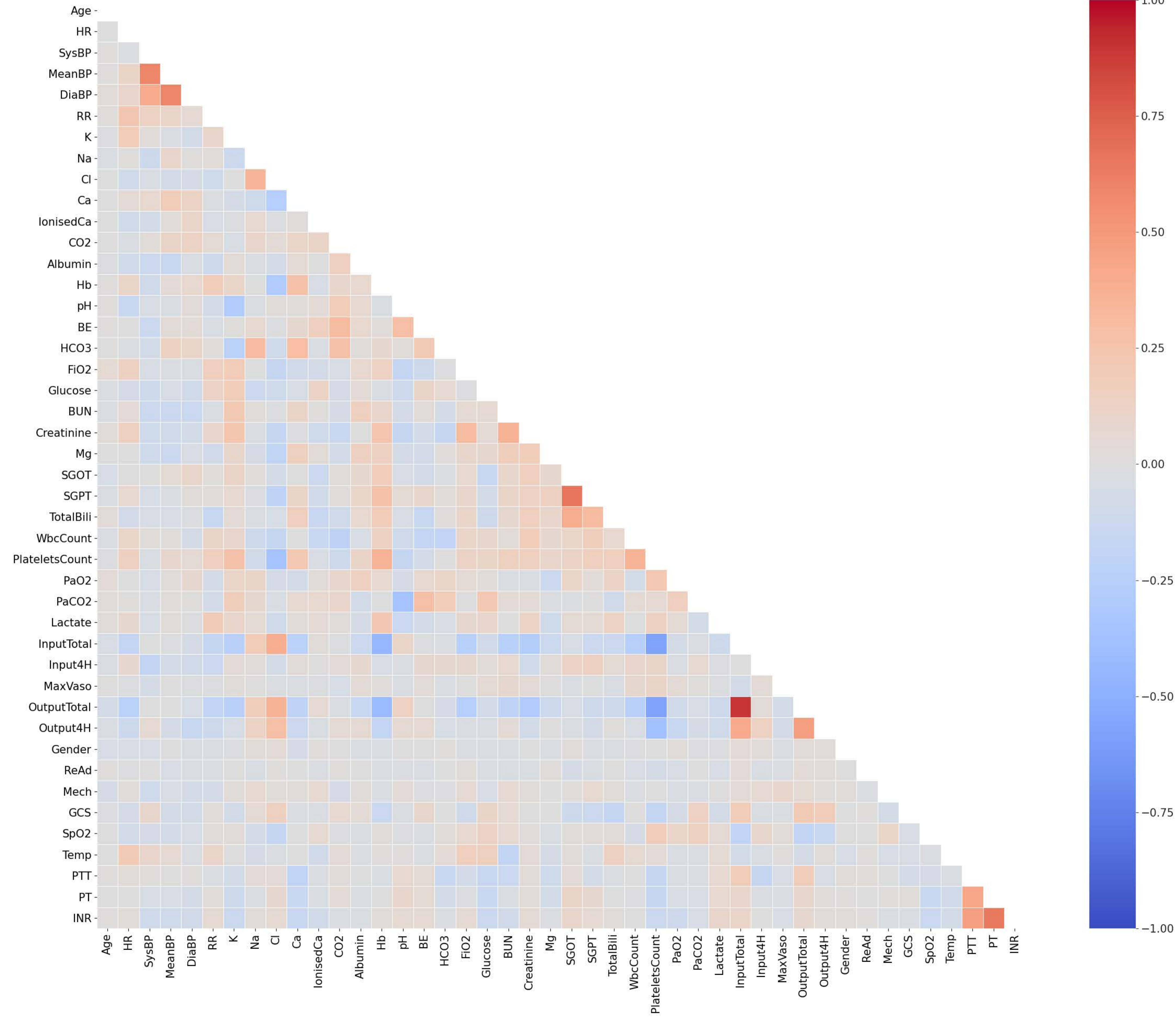

Supplement: Supplementary file 2 — Supplementary Figure 2 [file 41597_2022_1784_MOESM2_ESM.pdf]

Correlation Matrix: SyntheticData

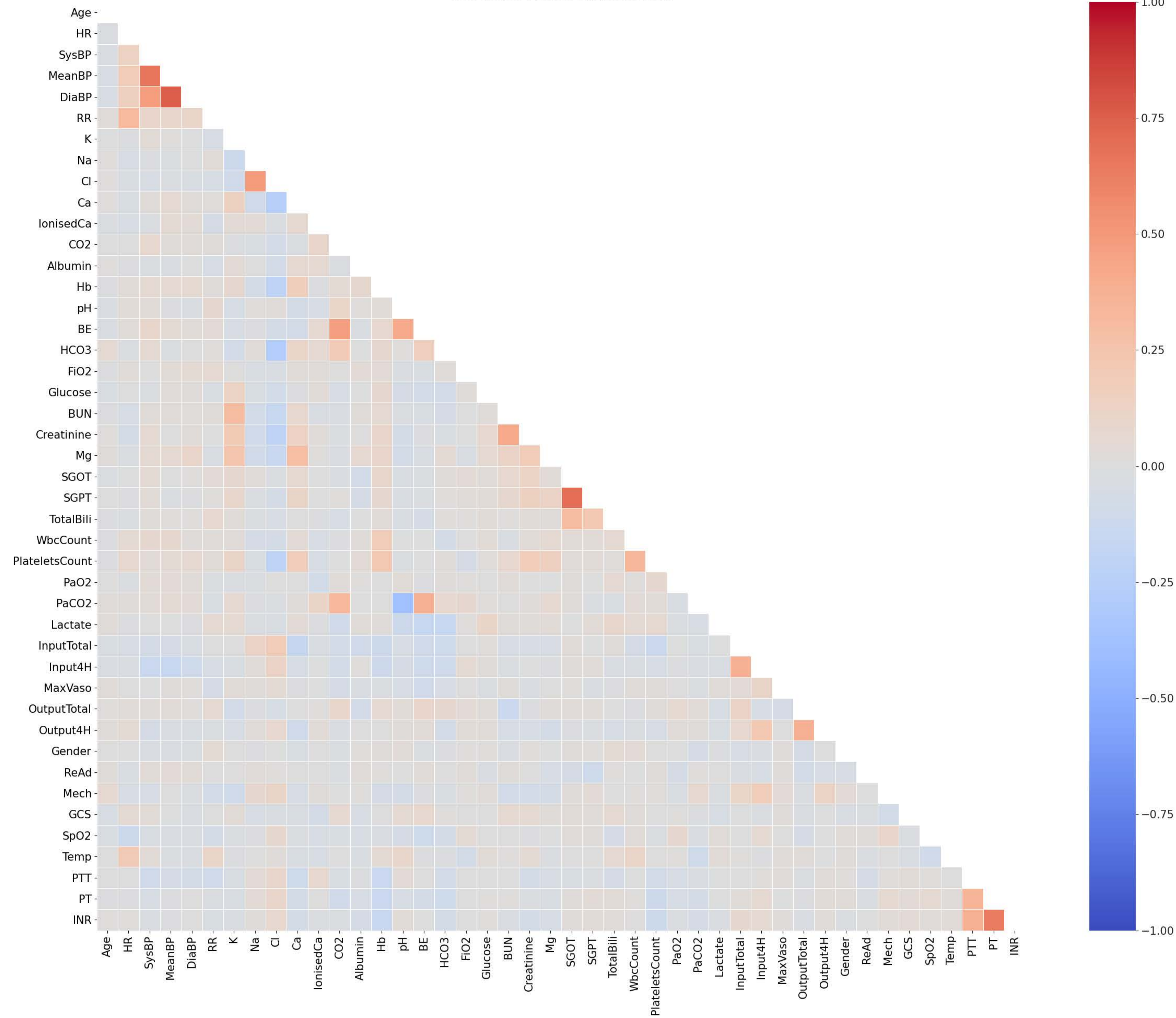

Correlation Matrix: RealData

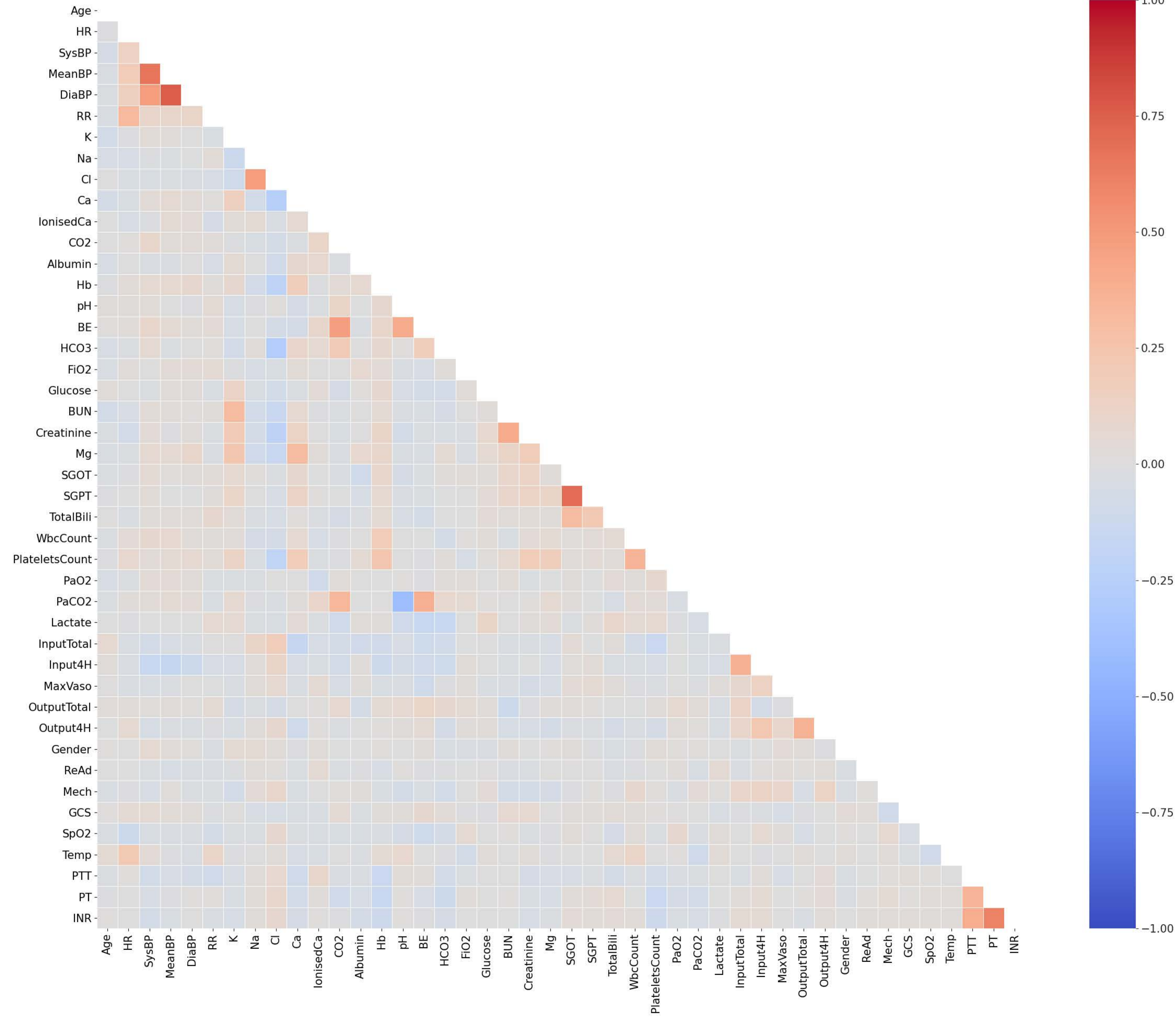

Supplement: Supplementary file 3 — Supplementary Figure 3 [file 41597_2022_1784_MOESM3_ESM.pdf]
