# Supplementary material for: The Health Gym: synthetic health-related datasets for the development of reinforcement learning algorithms
Source: Sci Data. 2022 Nov 11;9:693. doi: 10.1038/s41597-022-01784-7 (PMC9652426; doi:10.1038/s41597-022-01784-7)
Supplement: Supplementary file 4 — Supplementary Materials for the Health Gym Paper [file 41597_2022_1784_MOESM4_ESM.pdf]

# Supplementary Materials for the Health Gym Paper

**Nicholas I-Hsien Kuo[1,*], Mark N. Polizzotto[2], Simon Finfer[3, 4, 5], Federico Garcia[6], Anders Sönnerborg[7], Maurizio Zazzi[8], Michael Böhm[9], Rolf Kaiser[9], Louisa Jorm[1], and Sebastiano Barbieri[1]**

[1]Centre for Big Data Research in Health, University of New South Wales, Sydney, Australia
[2]Australian National University, Canberra, Australia
[3]The George Institute for Global Health, Sydney, Australia
[4]University of New South Wales, Sydney, Australia
[5]Imperial College London, London, United Kingdom
[6]Hospital Universitario San Cecilio, Granada, Spain
[7]Karolinska Institutet, Stockholm, Sweden
[8]Università degli Studi di Siena, Siena, Italy
[9]Uniklinik Köln, Universität zu Köln, Cologne, Germany
[*]Corresponding author: Nicholas I-Hsien Kuo (n.kuo@unsw.edu.au)

## ABSTRACT

In this study, we presented a novel approach for creating synthetic healthcare datasets (related to acute hypotension[1], sepsis[2], and HIV[3,4]) using the machine learning algorithm of GAN[5]. We carried out several statistical tests to verify the realisticness of the synthetic datasets and employed disclosure control techniques[6] to ensure that the public release of these datasets is associated with very low risk of sensitive information disclosure. This document of supplementary materials provides further technical details and discusses some of the concepts which were presented only briefly in the main body of the paper.

**Table of Contents**

1: Instructions on Deriving the Real Datasets
1.1: *Acute Hypotension Dataset*
1.2: *Sepsis Dataset*
1.3: *HIV Dataset*
2: The Training Details of GANs
2.1: *The Dimensionality in the GAN Setup*
2.2: *Remarks on Training the GAN Model*
3: The Statistical Tests of Stage 2
3.1: *The Two Sample KS Test*
3.2: *The Two Independent Sample Student's t-Test*
3.3: *The Snedecor's F-Test*
3.4: *The Three Sigma Rule Test*
3.5: *Iteratively Executing the Statistical Tests*
3.6: *The Statistical Outcomes*
4: The Correlations of Stage 3
4.1: *Kendall's Rank Correlation*
4.2: *Correlation Between Variables*
4.3: *Average Correlation in Trends and in Cycles*
4.4: *Remarks on Kendall's Rank Correlation and Alternative Validation Metrics*
5: Full Correlation Plots for Sepsis
6: Assessment of Disclosure Risk
6.1: *El Emam et al.'s Disclosure Risk Metrics*
6.2: *Alternative Disclosure Risk Metrics*
7: Verifying Utility through Reinforcement Learning
7.1: *The Acute Hypotension Reward Function*
7.2: *The Sepsis Reward Function*
7.3: *The HIV Reward Function*

# 1 Instructions on Deriving the Real Datasets

This section aims to clarify the details for preparing **The Real Datasets** described in the **Methods** section. Interested readers should also refer to the official website of MIMIC-III[7,8] at `https://physionet.org/content/mimiciii/1.4/`; and likewise consult the official website of EuResist[9] at `https://www.euresist.org/`.

## 1.1 Acute Hypotension Dataset

We mainly followed the instructions provided in Appendix D on page 15 of Gottesman *et al.*[10]. Although the authors published their codes in an open repository (see `https://github.com/dtak/interpretable_ope_public`), they did not release the SQL[11] code used to query the raw data from the MIMIC-III database. We derived a larger real hypotension dataset which consisted of a cohort of $3,910$ patients; there were $1,733$ distinct ICU admissions in Gottesman *et al.*'s original cohort of patients.

### 1.1.1 Inclusion/Exclusion Criteria

Gottesman *et al.* provided the selection criteria for their patient cohort in Appendix D.1 on page 15 of their paper. In brief, the authors included adult patients ($\geq$ 18 years old) in the MICU MIMIC-III sub-dataset with those with at least 24 hours of data collected from the MetaVision (MV) clinical information system. Then, they aggregated 48 hours of clinical variables from those patients with seven or more mean arterial pressure (MAP) values of 65 mmHg or less, which indicated probable acute hypotension.

### 1.1.2 Data Preparation

Almost all of the variables, except the Glasgow coma scale (GCS) score, are originally recorded as continuous numeric variables in the MIMIC-III database. GCS is a point-based system to reflect a patient's state of consciousness[12]. Fluid boluses, vasopressors, and fraction of inspired oxygen (FiO2) were treated as categorical variables, for reasons described below.

As documented in Appendix D.2 on page 15 of Gottesman *et al.*, most variables were queried *as-is* from the MIMIC-III database. However, total vasopressor measurements were derived from the administered fluids following the code provided by Komorowski *et al.*[2] (refer to *Vasopressors from MetaVision* in the script `AIClinician_Data_extract_MIMIC3_140219.ipynb` in `https://gitlab.doc.ic.ac.uk/AIClinician/AIClinician`).

After following the previous steps, we converted some variables from numeric to categorical as according to Appendix D.4 on page 16 of Gottesman *et al.*. In this section, Gottesman *et al.* explained that they categorised fluid boluses and vasopressors for training their RL algorithm in a discretised action space. Fluid boluses were binned into one of the four categories of *none, low, medium,* and *high* reflecting the numeric ranges of $0, [250, 500), [500, 1000),$ and $\geq 1000$, in mL units. Whereas for vasopressor, they binned the variable according to its first and third quantiles (*i.e.,* Q1 and Q3) and categorised vasopressor into the four classes of $0, (0, 8.1), [8.1, 21.58),$ and $\geq 21.58$ in the unit of total mcg of drug given each hour per kg body weight. However, our cohort of patients was slightly different to theirs and hence we binned vasopressors according to our re-calculated Q1 and Q3 values. Our vasopressor variable was thus categorised according to the ranges of $0, (0, 8.4), [8.4, 20.28),$ and $\geq 20.28$, respectively.

Another variable which we made categorical was FiO2. Since FiO2 was the *fraction* of inspired oxygen, we binned the variable into 10 classes according to the ranges of $[0, 0.1), [0.1, 0.2), \ldots, [0.8, 0.9),$ and $[0.9, 1.0]$.

### 1.1.3 Missing Values

As commented in Appendix D.5 on page 16, Gottesman *et al.* noted that the MIMIC-III database contains missing values. However, these missing values do not occur at random and can be highly informative. For instance, updated laboratory test are often ordered when a patient is transferred from one ICU to another. To deal with the missing values, we followed Gottesman *et al.* and constructed indicator variables (those binary variables with suffix (M)) to denote whether or not a value was measured. Missing values were then replaced with the last available recorded data.

## 1.2 Sepsis Dataset

We mainly followed the steps in Komorowski *et al.*[2]'s repository `https://gitlab.doc.ic.ac.uk/AIClinician/AIClinician` for preparing the real sepsis dataset. They queried the MIMIC-III database with their SQL file `AIClinician_Data_extract_MIMIC3_140219.ipynb` and processed the queired data with their codes in `AIClinician_sepsis3_def_160219.m`.

### 1.2.1 Inclusion/Exclusion Criteria

Komorowski *et al.* did not explicitly state the inclusion/exclusion criteria for the sepsis cohort in their paper. However, the processing code indicates that they only included adult patients. Then for all adults, they sought for any suspicious infections

based on the history of antibiotic administrations; and they included all clinical variables from *at least* 44 hours before the suspected infection and *up to* 28 hours after the infection. The use of such time period for defining a sepsis event is supported by a 2016 sepsis definition paper[13] which states that one should "*consider[s] a period as great as 48 hours before and up to 24 hours after onset of infection...*"; Komorowski *et al.* likely relaxed this condition with a -4/+4 window.

#### 1.2.2 Data Preparation

The data preparation procedure for the real sepsis dataset was simple, thanks to the code provided by Komorowski *et al.* in their repository. The file containing the SQL queries should be executed first, followed by the processing code. Further details are provided below.

We executed the entire file containing the SQL queries. The sepsis dataset contained a much larger subset of data from MIMIC-III than the hypotension dataset. Whereas the hypotension dataset only included patients from the MICU branch, the sepsis dataset included patients from the NICU, SICU, CSRU, CCU, MICU, and TSICU branches. Furthermore, while the hypotension dataset only considered those patients who had their information recorded by the MetaVision (MV) system, the sepsis dataset also included those patients whose data were collected by the CareVue (CV) system.

After executing the file containing the SQL queries, we executed lines 1 to 791 of the processing code. We did not execute the remaining lines of that code. This was because those lines were for deriving the secondary auxiliary items of P/F ratio, shock index, SOFA score, and SIRS criteria. For instance, the P/F ratio is computed by dividing $PaO_2$ by $FiO_2$, whereas the shock index is based on HR and systolic BP. All variables required to compute these secondary items are included in our dataset.

#### 1.2.3 Missing Values

Whereas Gottesman *et al.* chose to fill the missing values in the hypotension dataset by last observation carried forward, Komorowski *et al.* chose to impute the missing values. In line 739 of the procesing code, they mainly used Matlab's[14] built-in `knnimpute` function and their own code in `fixgaps.m`. They do not appear to have used the code contained in `fastknnsearch.m`.

### 1.3 HIV Dataset

We aimed to create a real HIV dataset from the EuResist[9] database with a similar purpose to the one that was presented in the work of Parbhoo *et al.*[3]. However, unlike the work of Gottesman *et al.* (for hypotension) and Komorowski *et al.* (for sepsis), Parbhoo *et al.* did not make their codes publicly available and thus we mainly consulted with HIV medical experts to create our own version of the real HIV dataset.

#### 1.3.1 WHO Guidelines

As mentioned in the **Methods** section, we based our real HIV dataset on the work of Parbhoo *et al.* but we also incorporated a published WHO guideline[4] which called for the standardisation of antiretroviral therapy for HIV. Chapter 4 "Clinical Guidelines: Antiretroviral Therapy", starting from page 71 in the referenced work, also accessible at `https://www.who.int/hiv/pub/arv/chapter4.pdf`.

The referenced WHO guideline contains an extensive discussion on the *strategic timing of antiretroviral treatment*. Prior to the WHO guideline, there was no standardised approach for initiating antiretroviral therapy in HIV. Antiretroviral therapy (ART) medications could be initialised when a person's CD4 count dropped either below 500 cells/$\mu$L (*i.e.,* moderate) or below 350 cells/$\mu$L (*i.e.,* critical). However, some studies have observed that early ART initiation increases the overall survival rate of people with HIV. Nonetheless, close monitoring in the first 3 months following ART initiation is required because people can develop hypersensitivity towards these medications, with potentially severe consequences.

More evidence on the benefits of the early adoption of ART medications can be found in a randomised controlled trial by the INSIGHT START Study Group[15]. In this longitudinal study over 4 years, the intervention group received immediate-initiation of ART, whereas the control group deferred-initiation. The median time until initiation in the control group was 3 years. The immediate-initiation group had either equivalent or better survival rates across a variety of AIDS-related and non-AIDS-related events (see Table 2 on page 7 in the referenced paper).

#### 1.3.2 Inclusion/Exclusion Criteria

Parbhoo *et al.* extracted their cohort of patients from the entire EuResist database. They included people who were treated with the 312 most common medication combinations (including 20 medications). They also included patient demographics; however, their code is not publicly available, and it is unclear which individual medications and demographic information were included in their dataset.

In order to create a similar dataset while incorporating information from the 2015 WHO guidelines[4], we restricted our cohort of patients to people initiated ART after 2015. Among the included population, we further selected those who were treated with the 50 most common medication combinations (including 21 medications). This choice is discussed further in the **Data Preparation** subsection below.

### 1.3.3 Data Preparation

Class Imbalance

There were a total of 11,252 people in the EuResist database who initiated ART after 2015. Among these people, there were 20 who identified as neither female nor male (*i.e.,* less than 0.02% of the total population). During our experiments, we found that our GAN model, consistently over more than 50 random initialisations, was unable to create datasets containing patient information related to this minority group. We therefore decided to exclude this group of people from our cohort; however, we recognise this as a limitation of our model that will need to be addressed by future algorithmic improvements.

Ethnicities in the EuResist database were also considerably imbalanced. They were originally categorised as Asian, African, Caucasian, Hispanic, Other, and Unknown. Since there were a very low number of 179 Hispanic people, we decided to combine Hispanic, Other, and Unknown into *Other*.

Persistent Low Standardisation in HIV ART

In Sections 1.3.1 and 1.3.2, we mentioned how our inclusion criteria were based on the publication of the WHO guidelines in 2015. Indeed, we observed several changes in medication regimens over time. Before 2015 and especially in the early 2000s, it was not uncommon to see a regimen composed of more than 10 medications. However, after 2015, almost all regimens contained less than 6 medications; with most regimens consisting of only 3 or 4 medications.

Despite the continuous changes, there is still a lack of standardisation in the practice of HIV ART therapy. For instance, there were a total of 1,179 different medication combinations prescribed post-2015; and this number greatly exceeded the 14 suggested first line treatment combinations for adults in the WHO guideline (see the first three rows of Table 4.1 on page 97 of the referenced work[4]). Upon closer analysis, we found that most medication combinations were only prescribed once, likely to suit just one patient's need. Moreover, we also found that the 10 most common medication combinations, which included a small subset of only 14 different medications, made up 34.1% of all prescription records. Thus, our aim was to extract a real HIV dataset which preserved the diversity in medication combinations while limiting an over-representation of single-use medication combinations.

Ultimately, we decided to include only the 50 most common medication combinations. For the 11,252 people in EuResist who initiated ART post-2015, there were 22,622 records (*i.e.,* rows in the database) in total. Including only the 50 most common medication combinations resulted in 17,660 records (78.1% of the total). Furthermore, the 50 most common medication combinations included 21 individual medications and made up 51.2% of all medication combinations prescribed post-2015.

Handling Long Gaps

Within our selected cohort, the shortest records were ten months long while the longest lasted for more than three years. However, a large proportion of people had long gaps in their records. This was most apparent in the EuResist regimen information table – someone who had just finished one therapy treatment would usually not be immediately prescribed with another treatment. We found that over a third of the people had one gap of over six months in their regimen treatment regimen; and that more than half of the remaining people had multiple (*i.e.,* two or three) treatment gaps in their records.

The use of data imputation techniques (such as splines) to fill these long gaps was unlikely to yield meaningful results. Instead, we decided to split the original records into several shorter sub-records describing continuous period of ART. Furthermore, to facilitate training of our GAN model, we truncated the sub-records' lengths to the closest multiple of ten (*e.g.,* 32 months were truncated at 30 months, and 51 months were truncated at 50 months).

Missing Values

Any other missing data values were filled in using last observation carried forwards, as discussed for the hypotension dataset in Section 1.1.3.

Decomposing Patient Regimens

After managing the gaps and missing values in the data, we investigated different ways to represent the ART treatment in a meaningful manner. As mentioned in the Persistent Low Standardisation in HIV ART subsection, our cohort were treated with 50 medication combinations including 21 individual medications. The medications belonged to five different classes: *nucleoside reverse transcriptase inhibitors* (NRTIs), *nucleotide reverse transcriptase inhibitors* (NtRTIs), *non-nucleotide reverse transcriptase inhibitors* (NNRTIs), *integrase inhibitor* (INI), and *protease inhibitors* (PIs). In addition to medications of the aforementioned classes, it was common among people in the EuResist database to be prescribed *pharmacokinetic enhancers* (pk-En) to boost the effectiveness of their medication combination.

ART medications tend to be associated with strong positive inter-class and strong negative intra-class correlations. For instance, if the INI medication of dolutegravir (DTG) was selected for a person's therapy, it was highly unlikely that another INI such as raltegravir (RAL) was required for the same therapy regimen. Contrarily, it is unnecessary to select an INI whenever

an NNRTI was already present in the regimen (see Table 4.1 on page 97 in the WHO guideline[4]). During preprocessing, we found that if we tried to represent each medication as a binary (or categorical) variable, we would yield datasets consisting of extremely strong positive and extremely negative correlations among its variables. We found that such datasets would lead our GAN model into creating under-diversified synthetic datasets. For instance, our generator sub-network could have heavily favoured the coexistence of emtricitabine (FTC), an NRTI, with DTG, an INI; and thus became unable to learn any association between FTC and the medication classes of NRTIs and INIs.

To address this problem, we decide to represent each medication regimens as a base drug combination supplemented with other auxiliary and secondary medications. For instance, a person can be treated with FTC + TDF + DTG + pk-En. We organise the available choice of base drug combination as one categorical variable. In addition, there is a categorical variable for NNRTIs, INIs, PIs, and pk-En representing every secondary medications. Since all NRTI and NtRTI medications are included in the base medication combination variable, it is unnecessary to separately create categorical variables representing these classes.

# 2 The Training Details of GANs

This section aims to provide more technical details on the training of **The Health Gym GAN** introduced in the **Methods** section. The codes for this paper are also made publicly available; see the **Data Records** section for more details.

Note that in our paper, we adopted the terminology given in the WGAN paper[16]. In the original GAN paper[5], the network that classified the real and fake data was a *discriminator*; but in the WGAN paper, there were theoretical modifications, and the discriminator turned into a *critic* function to measure the difference between the real and fake data using the Wasserstein distance. However for convenience, we will refer to both WGAN and GAN simply as GAN for the rest of this study.

**Summary of the GAN dimensionality:** Input dimension 128, hidden dimension 128, output dimension is dependent on the dataset. The embedded dimension of numeric data is 1; and it is 2 for binary data and 4 for categorical data.

## 2.1 The Dimensionality in the GAN Setup

As mentioned previously, the GAN setting included a generator $G$ and a critic $D$. The generator was trained to map vectors of multivariate Gaussian inputs to the synthetic data $G: z \to x_{\text{syn}}$; whereas the critic took a batch of either real data $x_{\text{real}}$ or synthetic data $x_{\text{syn}}$, and scored the realisticness of the data $D: x \to \mathbb{R}$. The important dimensionalities in this setup were thus associated with the variables of $z$, $x_{\text{syn}}$ and $x_{\text{real}}$, within the networks of $G$ and $D$, and the outputs of the two networks.

### *2.1.1 The Input and Hidden Dimensions of the Generator*

The dimensionality of the multivariate latent vectors $z$ was $\mathbb{R}^{\mathscr{I}}$ per synthetic patient per instance. That is, in order to generate a synthetic patient record over $T$ units of time, we would need a matrix of $z^{(1:T)} \in \mathbb{R}^{\mathscr{I} \times T}$; and we referred to $\mathscr{I}$ as the input dimension. Both $\mathscr{I}$ and $T$ were hyper-parameters and hence were selected prior to the training phase of the GAN model. For this work, we chose $\mathscr{I} = 128$ for all datasets. However, the values for $T$ varied across different datasets. This was because whereas the real hypotension data had 48 time points for all patients; the real sepsis data had non-uniform length for each patient – the shortest record was 2 time points while the longest was 20 time points. Likewise, the shortest real HIV data had 10 time points while the longest was of length 100. To generate synthetic datasets that closely represented their real counterparts, we selected $T = 48$ for hypotension, $T = 20$ for sepsis, and $T = 60$ for HIV.

Each instance of $z^{(1:T)}$ was forwarded to the generator network $G$ one at a time (recall that the first module in the generator is biLSTM). All modules in network $G$ had a dimensionality of $\mathscr{H}$. The mapping learnt by network $G$ was hence $\mathbb{R}^{\mathscr{I}} \to \mathbb{R}^{\mathscr{H}}$. We referred to $\mathscr{H}$ as the hidden dimension; it was a hyper-parameter and we selected it to be $\mathscr{H} = 128$ across all datasets.

### *2.1.2 The Output Dimension of the Generator and the Contents of $X_{\text{syn}}$ and $X_{\text{real}}$*

The output of the generator was also the input of the critic. Hence the output dimension of the generator $\mathscr{O}$ not only described the amount of features in the synthetic data $X_{\text{syn}}$, but it was also identical to the input dimension of the critic network. Since the synthetic data mimicked the real data $X_{\text{real}}$, the dimensionality of $\mathscr{O}$ varied across different datasets because each real dataset comprised a different number of clinical variables. As reported in the **Methods** section, there were 20 variables for acute hypotension, 44 for sepsis, and 13 for HIV. However, the output dimensions were not 20, 44, and 13.

The reason for this was the presence of various data types in our datasets. There were continuous numeric variables (*e.g.,* systolic BP), binary variables (*e.g.,* gender), and also categorical variables (*e.g.,* ethnicity). Whereas the numeric variables could be forwarded directly as float values to the critic, binary and categorical variables required additional transformations to present them in a machine-readable format to the critic.

**Pre-processing Variables in $X_{\text{real}}$**

For hypotension, we first transformed each real numeric variable according to their optimal *Box-Cox*[17] transformation using the `stats.boxcox` function in the `Python`[18] package of `Scipy`[19]. Afterwards, we re-centred and re-scaled the variables within the range of $[0,1]$ with the `preprocessing.MinMaxScaler` scaler of the `Python`[18] package of `Scikit-learn`[20].

For sepsis, we first analysed if the real numeric variables had extremely long tails. As discussed in the **Data Records** section, we decided to subdivide numeric variables with extremely long tails into deciles and process them as categorical variables. The remaining real numeric variables were log-transformed if necessary, before re-centring and re-scaling within the range of $[0,1]$ with `preprocessing.MinMaxScaler`.

Numeric variable processing was slightly easier for the HIV dataset. This was because the only numeric variables were VL and CD4. The former required an optimal Box-Cox transformation followed with re-centring and re-scaling to $[0,1]$; whereas the latter required log-transformation followed by re-centring and re-scaling to $[0,1]$.

While processing the numeric variables of the real datasets $X_{\text{real}}$, it was important to record the transformation procedure for every individual variable. Only by doing so, we would be able to back-transform (*i.e.,* revert the re-centring and re-scaling

procedure) the synthetic data $X_{\text{syn}}$ generated by network $G$. The dimensionality of real continuous numeric variables (without an extremely long tail) was preserved after pre-processing. Binary and categorical variables were converted into one-hot-encoded vectors with total length equal to two (for binary variables) and equal to the number of unique classes (for categorical variables).

**Example: Pre-processing the Real Acute Hypotension Dataset**

There were 20 variables in the real hypotension dataset. The dataset comprised 9 numeric variables, 4 categorical variables, and 7 binary variables. After transforming the 9 numeric variables, there were still 9 features. The transformation of the 7 binary variables resulted in 14 features; and there were $4+4+10+13 = 31$ unique classes for the categorical variables of fluid boluses, vasopressors, FiO2, and GCS. Hence, the output dimension of the generator network $G$ was $\mathscr{O} = 9+14+31 = 54$. Note that this was the feature dimension for both $X_{\text{real}}$ and $X_{\text{syn}}$.

**Case Study: An Interpretation of the Output of the Generator for Hypotension**

As mentioned earlier in this appendix, the generator network for hypotension served as a mapping for $G : z \to x_{\text{syn}}$ from dimension $\mathbb{R}^{128} \to \mathbb{R}^{54}$. Dimensions $1-9$ corresponded to the clinical variables in the strict order of MAP, diastolic BP, systolic BP, urine, ALT, AST, PaO2, lactate, and serum creatinine, respectively. Furthermore, since all of the real numeric variables were readjusted to lie within the range of $[0,1]$ (see **Pre-processing Variables in $X_{\text{real}}$**), we passed the first 9 dimensions of the generator output to the `sigmoid` activation function to match the extreme values allowed for the numeric variables.

Dimensions $10-40$ corresponded to the categorical variables. It was $10-13$ for fluid boluses, $14-17$ for vasopressors, $18-27$ for FiO2, and $28-40$ for GCS. To represent these dimensions of the synthetic data as categorical variables, we passed the corresponding dimensions to a `softmax` activation function. For instance, after passing dimension $10-13$ through `softmax`, dimension 10 represented class I of fluid boluses; and likewise, 11 for class II, 12 for class III, and 13 for class IV for the fluid boluses categories, respectively. A similar procedure was repeated for all other categorical variables, and likewise for all the binary variables (dimensions 41-54).

### 2.1.3 On Soft Embedding

Before discussing the dimensionalities of the critic network $D$, we need to address the soft-embedding function. The input to network $D$ was either $x_{\text{syn}}$ or $x_{\text{real}}$. As depicted in the figure, all continuous numeric variables were treated as-is from the data, but the binary and categorical variables were subject to a soft embedding transformation. We created a trainable embedding matrix $W_{\text{emb}}$ for every binary or categorical variable. The weights were of size $\mathbb{R}^{(\alpha,\beta)}$ where $\alpha$ denotse the size of the unique classes of the binary or categorical variables and $\beta$ denotes the pre-specified size of the projection (2 for binary variables and 4 for categorical variables). The soft embedding helped us to represent the binary and categorical variable features as vectors of high dimensional floating values $x^{\text{T}} W_{\text{emb}}$ for the critic.

**Case Study: Soft Embedding for the Hypotension Data**

Let us denote $X$ as the hypotension data prior to soft embedding and refer to $U$ as the data after soft embedding. The first 9 dimensions of the hypotension data $X^{(1:9)}$ represented continuous numeric variables and were therefore not subject to soft embedding. Then for each binary or categorical variable, we defined a unique matrix $W_{emb}$. If the variable was binary, we would set $\beta = 2$; and categorical variables had $\beta = 4$ instead. These were hyper-parameters that were specified before training the GANs model; and these settings were applied to all of the synthetic datasets.

Of the 20 variables in the hypotension data $X \in \mathbb{R}^{54}$, the one-hot-encoded binary variables for urine (M) was stored in $X^{(41:42)}$. Thus, the dimension of the soft embedding matrix for urine (M) was $2 \times 2$ and the soft embedding procedure was hence $U^{\text{urine (M)}} = X^{(41:42)^{\text{T}}} W_{\text{emb}}^{\text{urine (M)}}$ resulted in a continuous feature vector of size 2.

As an additional example, the information related to GCS was stored in $X^{(28:40)}$. The dimension of the soft embedding matrix for GCS was 13 (the number of unique classes of GCS) by 4 (the projection size for categorical variables). The soft embedding of GCS was hence $U^{\text{GCS}} = X^{(28:40)^{\text{T}}} W_{\text{emb}}^{\text{GCS}}$ resulting in a continuous feature vector of size 4.

After the binary and categorical variables were transformed through soft embedding, they were concatenated with the numeric variables such that $U = [X^{(1:9)} \bigoplus U^{\text{Fluid Boluses}} \bigoplus U^{\text{Vasopressors}} \bigoplus \ldots \bigoplus U^{\text{Serum Creatinine (M)}}]$. Hence, the total dimension of the post-embedding vector $U$ was $9+4\times 4+7\times 2 = 39$; that is, 9 dimensions for numeric with feature of size 4 for the 4 categorical variables and feature vectors of size 2 for the 7 binary variables in the hypotension data. In conclusion, the input dimension of the critic network $D$ was 39 for the hypotension dataset.

### 2.1.4 On the Dimensionality of the Critic

The critic $D$ had an input dimension that varied across task (see Section 2.1.3), but it had a fixed hidden dimension of size 128, and a fixed output dimension of 1. The output dimension of the critic was not a hyper-parameter; instead, it was fixed as 1 due to the design of the GAN architecture[5]. This was because that the output of the critic corresponded to a realisticness score of the data $X$ where $D(X) \in \mathbb{R}$.

## 2.2 Remarks on Training the GAN Model

The scheme for training the WGAN-GP was mostly identical to the setting in the original paper[21]. The Health Gym GAN received the training data in batches of size 32 and was updated using the *Adam* optimiser[22] with learning rate $1 \times 10^{-3}$. The model was regularised with $\lambda_{\text{GP}} = 10$ for the gradient penalty loss and $\lambda_{\text{corr}} = 10$ for the alignment loss. To stabilise the learning curve of the model, we adopted a *curriculum learning*[23,24] strategy where we first trained the GAN to create short sequences and then progressively generated longer synthetic time series. Furthermore, the generator network was updated once for every 5 updates of the critic network (see Algorithm 1 on page 4 of the referenced work[21]).

The Health Gym GAN was capable of generating relatively good data after the first 100 epochs of training. However, we found that the first 100 epochs of training could be highly unstable, causing the quality of the synthetic data to vary greatly (mostly from seed to seed). The quality of the generated data stabilised when we prolonged the training to 500 epochs. This was when we terminated the training of the GAN model and used the generator network to synthesise our datasets.

**The Final Transformation After Synthetic Data Generation**

As mentioned in Appendix 2.1.2, all of the synthetic data $X_{\text{syn}}$ were passed through either a `sigmoid` activation function (for the numeric variables) or a `softmax` (for the binary or categorical variables). To ensure that each synthetic variable could be meaningfully interpreted, it was necessary to execute a back-transformation.

For continuous numeric variables this involved reverting the centring and scaling steps; and also applying the inverse of any prior power transformations. For the binary and categorical variables we only had to seek the largest probability in the `softmax` activated values. For instance, since the hypotension GCS features were stored in $X_{\text{syn}}^{(28:40)}$, we would have to find which value among those dimensions was the largest. Say that it was dimension 29, then it meant that the second class of the GCS features was the most probable – and hence it was a GCS of score 4 (remember that GCS scores count from 3, so that $3+1=4$).

## 3 The Statistical Tests of Stage 2

This section contains the definitions of the statistical test used in stage 2 of our realisticness validation procedure and a summary of the statistical outcomes.

### 3.1 The Two Sample KS Test

The null hypothesis of the two sample KS test assumes that there are

*no significant differences between the distributions*

of the synthetic data and the real data for a specific variable $X$. The KS test accepts both numeric and categorical data types. Given a sample of data $B_S(\mathbf{x})$ from the synthetic variable and a sample of data $B_R(\mathbf{x})$ from the real variable, the test computes the statistic

$$K_{B_S,B_R} = \sup_{\mathbf{x}} \left| C_{B_S}(\mathbf{x}) - C_{B_R}(\mathbf{x}) \right| \tag{1}$$

based on the supremum of two *empirical cumulative distribution functions* (ECDFs) $C$; with $C_{B_S}$ and $C_{B_R}$ denoting the ECDFs of $B_S(\mathbf{x})$ and $B_R(\mathbf{x})$ respectively. The ECDFs are computed as

$$C_B = \frac{1}{n_B} \sum_{j=1}^{n_B} \mathbf{1}_{(-\infty,\mathbf{x}]}(x_j) \tag{2}$$

for $x_j \in B(\mathbf{x})$ of size $n_B$. We denote $\mathbf{1}_{(-\infty,\mathbf{x}]}(x_j)$ as the indicator function: 1 if $x_j \leq \mathbf{x}$ and 0 otherwise. The null hypothesis is rejected if the statistic $K_{B_S,B_R}$ exceeds a critical value with a statistical significance level $\alpha_{KS}$ following the Kolmogorov distribution[25,26]; we use $\alpha_{KS} = 0.05$. In our work, we implemented the two sample KS test with the `stats.ks_2samp` function of the `Python`[18] package of `Scipy`[19].

### 3.2 The Two Independent Sample Student's t-Test

The null hypothesis of the two independent sample Student's t-test assumes that there are

*no significant differences between the means*

of the synthetic data and the real data for a specific variable $X$. The t-test only accepts numeric data types. Following the notations given in Appendix 3.1, the test statistic is

$$t_{B_S,B_R} = \frac{\overline{B_S(\mathbf{x})} - \overline{B_R(\mathbf{x})}}{S} \sqrt{\frac{n_{B_S} n_{B_R}}{n_{B_S} + n_{B_R}}} \tag{3}$$

where $\overline{B_S(\mathbf{x})}$ and $\overline{B_R(\mathbf{x})}$ refers to the respective sample means of $B_S(\mathbf{x})$ and $B_R(\mathbf{x})$; and the pooled standard deviation $S$ is

$$S = \sqrt{\frac{S^2_{B_S} + S^2_{B_R}}{2}} \tag{4}$$

where $S^2_{B_S}$ and $S^2_{B_R}$ denote the respective unbiased estimators for the variances of $B_S(\mathbf{x})$ and $B_R(\mathbf{x})$. The null hypothesis is rejected if the statistic $t_{B_S,B_R}$ exceeds a critical value of the Student's t-distribution[27] with a pre-determined threshold level $\alpha_t$; we use $\alpha_t = 0.05$. In our work, we implemented the two independent sample t-test with the `stats.ttest_ind` function of the `Python`[18] package of `Scipy`[19].

### 3.3 The Snedecor's F-Test

The null hypothesis of the Snedecor's F-test assumes that there are

*no significant differences between the variances*

of the synthetic data and the real data for a specific variable X. The F-test accepts both numeric and categorical data types. Following the notations given in Appendices 3.1 and 3.2, the test statistic for the numeric case is

$$F_{B_S,B_R} = \frac{S^2_{B_S}}{S^2_{B_R}} \tag{5}$$

and it measures the deviation of the two population variances. The more this value deviates from 1, the stronger the evidence for unequal variances. The null hypothesis is rejected if the statistics $F_{B_S,B_R}$ is greater than a pre-determined critical value from the F distribution[28] with significance level $\alpha_F$; we use $\alpha_F = 0.05$. We implemented the F-test with the `stats.f.cdf` function of the `Python`[18] package of `Scipy`[19]. For the categorical case, the F-statistic measured the between-group variance against the within-group variance instead; and it was implemented with the `stats.f_oneway` function of `Scipy`.

### 3.4 The Three Sigma Rule Test

The three sigma rule test, also known as the 68-95-99.7 rule test, is an empirical rule to determine whether a value (or a set of values) lies within an interval estimate of a normal distribution. A variable $X$ sampled from a normal distribution with mean $\mu$ and standard deviation $S$ has the following approximated probabilities of lying within one, two, or three standard deviations from the mean:

$$\mathbb{P}(\mu - S \leq X \leq \mu + S) \approx 68\%, \tag{6}$$
$$\mathbb{P}(\mu - 2S \leq X \leq \mu + 2S) \approx 95\%, \text{ and} \tag{7}$$
$$\mathbb{P}(\mu - 3S \leq X \leq \mu + 3S) \approx 99.7\%. \tag{8}$$

In this study, we used the three sigma rule test to assess whether a synthetic variable covered a similar range as the real counterpart if it failed the KS test. This test was added because both the t-test and the F-test could only reveal potential shortcomings in the synthetic data distribution, but they could not be used to determine whether the synthetic data was still acceptable for downstream machine learning applications if any of the synthetic variables were to fail the statistical tests. For all synthetic variables, we chose to use the two standard deviation confidence interval (CI with $\pm 2S$).

### 3.5 Iteratively Executing the Statistical Tests

**Algorithm 1** The Psuedo-Code for Executing Stage 2

$\eta_{KS} = \eta_t = \eta_F = \eta_{3S} = 0$ ▷ Initiate the counters as 0.

**for** $\xi = 1, \Xi$ **do** ▷ Run the statistical tests for $\Xi$ iterations.
  $\{\}_{3S} = \emptyset$ ▷ Instantiate an empty set for the three sigma rule test.

  $B^*_S(\mathbf{x}) \sim X_{\text{syn}}$ and $B^*_R(\mathbf{x}) \sim X_{\text{real}}$ ▷ Sample raw data with size $n_B$.
  $B_S(\mathbf{x}) = \texttt{transform}\left(B^*_S(\mathbf{x})\right)$ and $B_R(\mathbf{x}) = \texttt{transform}(B^*_R(\mathbf{x}))$ ▷ Optional `minmax` transformation.

  **if** $X_{\text{real}}$ is numeric **then**
    $P_{KS} = \texttt{KS test}(B_S(\mathbf{x}), B_R(\mathbf{x}))$ ▷ Acquire $p$-values for the statistical hypothesis test.
    $P_t = \texttt{t-test}(B_S(\mathbf{x}), B_R(\mathbf{x}))$
    $P_F = \texttt{F-test}(B_S(\mathbf{x}), B_R(\mathbf{x}))$

    $\varepsilon_{\text{lower}}, \varepsilon_{\text{upper}} = \mu(B_R(\mathbf{x})) \pm 2S(B_R(\mathbf{x}))$ ▷ Compute real value CI with 2 standard deviations.
    $\{\}_{3S} = \{\}_{3S} \cup_{i=1}^{n_B} B_S(\mathbf{x}_i)$ if $B_S(\mathbf{x}_i) \in [\varepsilon_{\text{lower}}, \varepsilon_{\text{upper}}]$ ▷ Append the list with synthetic values within the CI.

    $\eta_{KS}$ += 1 if $P_{KS} > \alpha_{KS}$ ▷ Increase the counter if the null hypothesis is kept.
    $\eta_t$ += 1 if $P_t > \alpha_t$ ▷ (Note the ">" sign.)
    $\eta_F$ += 1 if $P_F > \alpha_F$

    $\eta_{3S}$ += 1 if $|\{\}_{3S}| > \tau_{3S}$ ▷ Increase the counter if the majority of synthetic data is in the CI.
  **end if**

  **if** $X_{\text{real}}$ is categorical **then**
    $P_{KS} = \texttt{KS test}(B_S(\mathbf{x}), B_R(\mathbf{x}))$
    $P_F = \texttt{F-test}(B_S(\mathbf{x}), B_R(\mathbf{x}))$

    $\eta_{KS}$ += 1 if $P_{KS} > \alpha_{KS}$
    $\eta_F$ += 1 if $P_F > \alpha_F$
  **end if**
**end for**

We summarised the execution procedure for the statistical tests in the pseudo-code above. The purpose was to compare a synthetic variable $X_{\text{syn}}$ against its real counterpart $X_{\text{real}}$. Since the synthetic datasets of this paper were primarily designed for machine learning algorithms including RL, we tested the generated data using repeated mini-batch sampling, a scenario that

resembles the iterative training phase[29] of a neural network. The iterative process also helped us estimate the Type II error of the statistical tests. A detailed description of the pseudo-code is provided below.

In order to mimic the iterative training phase of a nueral network, we ran the statistical tests for a pre-specified maximum number of iterations $\Xi = 100$ (Line 3) with a sampling size of $n_B = 32$ (Line 6). Prior to the tests, we defined $\eta_{KS}$, $\eta_t$, $\eta_F$, $\eta_{3S}$ as the counters for non-rejected KS tests, t-tests, F-tests, and three sigma rule tests, respectively (Line 1). Numeric variables were rescaled as in the pre-processing step for machine learning model building (Line 7). We increased the counters by 1 whenever we *kept* a null hypotheses (Lines 17 - 19), *i.e.,* when the $p$-values were greater than the statistical significance level of $\alpha = 0.05$. When the iterations ended, we used the counters as indicators of the realisticness of the synthetic variables. If the final counter values were 70% or higher than the amount of total iterations ($\eta > 0.7\ \Xi$), we concluded that the synthetic variable had accurately captured the distributional features of the real variable. Of note, only the KS test and F-test were suitable for categorical variables. The three sigma rule test is not a statistical hypothesis test, and is discussed separately below.

The 70% threshold was chosen arbitrarily. However, it was because that it was a relatively strict threshold for preventing Type I error, since we only regarded synthetic variables to be realistic when 70% of the trials were associated with less than 5% risk (the 5% significance level) of concluding that a difference existed when there was no true difference. For completeness, we reported the *rates* of passing each test for all variables in the tables of Section 3.6.

To conduct the three sigma rule test, we instantiated an empty set $\{\}_{3S}$ at the beginning of each iteration (Line 4) and we set the $2S$ interval as the range for reliable values (Line 14). After defining the lower and upper bounds, we appended $\{\}_{3S}$ with synthetic variables that lied within the defined range (Line 15). We set a threshold $\tau_{3S} = 0.7\ n_B$; that is, we considered a mini-batch of synthetic variable passing the three sigma rule test if more than 70% of the synthetic data of that batch fell within the two standard deviation range (Line 21). Finally, if more than 70% of all random mini-batches passed the aforementioned threshold ($\eta_{3S} > 0.7\ \Xi$), we considered the synthetic variable to be sufficiently accurate even if it had failed the KS test.

No multiple testing corrections were applied to adjust the significant thresholds $\alpha$s. Multiple testing corrections (*e.g.,* the Bonferroni correction[30]) are commonly used to lower the chance for Type I errors when multiple hypothesis tests are conducted in parallel. However, since we *desire the null hypothesis to be kept* (see Lines 17, 18, 19, 28, and 29 of Algorithm 1), multiple testing corrections would increase the chances of showing our synthetic datasets being realistic thus giving us an advantage.

## 3.6 The Statistical Outcomes

The content below supplements the **Validation Outcomes** section of the **Technical Validation**.

### *3.6.1 Acute Hypotension*

| Variable Name | KS-Test | t-Test | F-Test | Three Sigma Rule Test |
|---|---|---|---|---|
| MAP | 96/100 | 95/100 | 86/100 | 100/100 |
| Diastolic BP | 92/100 | 94/100 | 93/100 | 100/100 |
| Systolic BP | 98/100 | 98/100 | 91/100 | 97/100 |
| Urine | 95/100 | 92/100 | 96/100 | 100/100 |
| ALT | 62/100 | 94/100 | 90/100 | 94/100 |
| AST | 59/100 | 95/100 | 91/100 | 97/100 |
| PaO2 | 40/100 | 94/100 | 88/100 | 97/100 |
| Lactic Acid | 74/100 | 96/100 | 83/100 | 98/100 |
| Serum Creatinine | 90/100 | 94/100 | 94/100 | 100/100 |
| Fluid Boluses | 100/100 | - - | 84/100 | - - |
| Vasopressors | 100/100 | - - | 95/100 | - - |
| $FiO_2$ | 100/100 | - - | 93/100 | - - |
| GCS | 97/100 | - - | 95/100 | - - |
| Urine (M) | 97/100 | - - | 91/100 | - - |
| ALT/AST (M) | 100/100 | - - | 75/100 | - - |
| $FiO_2$ (M) | 100/100 | - - | 85/100 | - - |
| GCS (M) | 100/100 | - - | 95/100 | - - |
| $PaO_2$ (M) | 100/100 | - - | 86/100 | - - |
| Lactic Acid (M) | 100/100 | - - | 88/100 | - - |
| Serum Creatinine (M) | 100/100 | - - | 92/100 | - - |

**Table 1.** The Stage Two Statistics for Acute Hypotension

An overview of the statistic tests for the synthetic hypotension dataset is presented in Table 1. As described in Section 3.5, the statistical tests were repeated for $\Xi = 100$ iterations and were considered “passed” if the null hypothesis was not rejected 70% or more of the times ($\eta > 0.7\ \Xi$); the numerators correspond to the number of times that the null hypothesis was not rejected. For instance, MAP with a score of 96/100 meant that 96 out of 100 times we kept the null hypothesis of the KS-test at a significance level of 0.05. This passes the 70% mark and is highlighted in blue. Failed tests are highlighted in red.

All variables in the synthetic hypotension datasets are realistic. Although 3 variables (ALT, AST, and PaO2) fail the KS test, their means and variances are captured appropriately; and all variables are within plausible ranges of the real variables.

### 3.6.2 Sepsis

The results of sepsis are summarised in Tables 2 and 3. Of all the 44 variables, only Max Vaso behaves undesirably. However, as we previously mentioned in the **Technical Validation** section, this was because Max Vaso was highly skewed. As described in the **Data Records** section, Max Vaso was not transformed into a categorical variable because its most related variables (*i.e.,* Input Total, Input 4H, Output Total, and Output 4H) were numeric.

Nonetheless, the synthetic Max Vaso variable performed well regarding the three sigma rule test, indicating that the synthetic variable was within a plausible range of the real variable values. To conclude, the synthetic sepsis dataset is highly realistic.

| Variable Name | KS Test | t-Test | F-Test | Three Sigma Rule Test |
|---|---|---|---|---|
| Age | 96/100 | 96/100 | 98/100 | 100/100 |
| HR | 98/100 | 95/100 | 95/100 | 100/100 |
| Systolic BP | 96/100 | 93/100 | 96/100 | 100/100 |
| Mean BP | 94/100 | 95/100 | 92/100 | 100/100 |
| Diastolic BP | 95/100 | 94/100 | 92/100 | 100/100 |
| RR | 97/100 | 95/100 | 95/100 | 98/100 |
| K | 94/100 | 97/100 | 91/100 | 100/100 |
| Na | 97/100 | 95/100 | 91/100 | 100/100 |
| $Cl^-$ | 96/100 | 95/100 | 97/100 | 99/100 |
| Ca | 95/100 | 97/100 | 87/100 | 100/100 |
| Ionised Ca | 93/100 | 92/100 | 85/100 | 99/100 |
| $CO_2$ | 92/100 | 93/100 | 93/100 | 99/100 |
| Albumin | 96/100 | 95/100 | 96/100 | 99/100 |
| Hb | 96/100 | 95/100 | 96/100 | 100/100 |
| pH | 98/100 | 98/100 | 88/100 | 100/100 |
| BE | 90/100 | 94/100 | 90/100 | 98/100 |
| $HCO_3$ | 95/100 | 95/100 | 94/100 | 100/100 |
| $FiO_2$ | 91/100 | 99/100 | 94/100 | 99/100 |
| Glucose | 96/100 | 96/100 | 89/100 | 99/100 |
| BUN | 94/100 | 94/100 | 93/100 | 100/100 |
| Creatinine | 94/100 | 94/100 | 90/100 | 100/100 |
| Mg | 90/100 | 93/100 | 90/100 | 98/100 |
| SGOT | 92/100 | 96/100 | 84/100 | 98/100 |
| SGPT | 90/100 | 89/100 | 93/100 | 100/100 |
| Total Bili | 91/100 | 91/100 | 87/100 | 100/100 |
| WBC | 97/100 | 99/100 | 93/100 | 100/100 |
| Platelets | 94/100 | 96/100 | 95/100 | 99/100 |
| $PaO_2$ | 97/100 | 98/100 | 95/100 | 100/100 |
| $PaCO_2$ | 95/100 | 95/100 | 95/100 | 99/100 |
| Lactate | 97/100 | 97/100 | 84/100 | 100/100 |
| Input Total | 95/100 | 95/100 | 84/100 | 99/100 |
| Input 4H | 83/100 | 94/100 | 94/100 | 100/100 |
| Max Vaso | 0/100 | 96/100 | 30/100 | 97/100 |
| Output Total | 92/100 | 95/100 | 89/100 | 99/100 |
| Output 4H | 90/100 | 96/100 | 90/100 | 100/100 |

**Table 2.** A Subset of Stage Two Statistics for Sepsis

| Variable Name | KS-Test | t-Test | F-Test | Three Sigma Rule Test |
|---|---|---|---|---|
| Gender | 88/100 | - - | 64/100 | - - |
| Readmission | 97/100 | - - | 88/100 | - - |
| Mech | 100/100 | - - | 96/100 | - - |
| GCS | 93/100 | - - | 86/100 | - - |
| $SpO_2$ | 97/100 | - - | 96/100 | - - |
| Temp | 95/100 | - - | 91/100 | - - |
| PTT | 94/100 | - - | 87/100 | - - |
| PT | 97/100 | - - | 96/100 | - - |
| INR | 99/100 | - - | 97/100 | - - |

**Table 3.** The Remaining Stage Two Statistics for Sepsis

### 3.6.3 HIV

| Variable Name | KS-Test | t-Test | F-Test | Three Sigma Rule Test |
|---|---|---|---|---|
| VL | 48/100 | 90/100 | 15/100 | 100/100 |
| CD4 | 83/100 | 97/100 | 91/100 | 91/100 |
| Rel CD4 | 85/100 | 92/100 | 100/100 | 99/100 |
| Gender | 97/100 | - - | 49/100 | - - |
| Ethnic | 81/100 | - - | 38/100 | - - |
| Base Drug Combo | 73/100 | - - | 71/100 | - - |
| Comp. INI | 96/100 | - - | 73/100 | - - |
| Comp. NNRTI | 92/100 | - - | 95/100 | - - |
| Extra PI | 100/100 | - - | 73/100 | - - |
| Extra pk-En | 100/100 | - - | 95/100 | - - |
| VL (M) | 100/100 | - - | 72/100 | - - |
| CD4 (M) | 99/100 | - - | 94/100 | - - |
| Drug (M) | 100/100 | - - | 88/100 | - - |

**Table 4.** The Stage Two Statistics for HIV

The results for HIV are summarised in Table 4. Of all the 13 variables, only VL failed the KS-test. Similar to Max Vaso in sepsis, VL was highly skewed and also failed the F-test. This shows the difficulty in appropriately capturing the variability of highly skewed distributions. Nonetheless, we found that VL was still realistic as it passed the three sigma rule test with very good scores.

Both gender and ethnicity required extra attention. Even though both variables passed the KS-test, they did not perform well on the ANOVA F-test. In fact, several binary and categorical variables in the synthetic HIV dataset only achieved acceptable (70%+), instead of ideal (preferrably around 85%+), scores for their F-tests. While we were unable to explain the exact reason for this, we speculated that this was due to the combination of variables in the dataset. Unlike the hypotension and sepsis datasets, the HIV dataset contained very few numeric variables. Therefore, it is possible that categorical variables were more difficult to synthesise with GANs.

Overall, we believe that the synthetic HIV dataset is still realistic. All but one variable passed the KS-test, and all numeric variables performed very well on the three sigma rule test.

# 4 The Correlations of Stage 3

This section contains details on the correlation assessments that we used in stage 3 of the **Realisticness Validation Procedure** in the **Technical Validation** section. The definitions of each test and some potential alternatives are discussed below.

## 4.1 Kendall's Rank Correlation

The non-parametric Kendall rank correlation, otherwise known as Kendall's $\tau$ correlation, is a statistic that measures the strength of association between two variables. In this study, we used the $\tau$-b variant and we refer to the original paper[31] for its precise definition.

The magnitude of the correlation between a pair of variables, $X^{(i)}$ and $X^{(j)}$ for $i \neq j$, is measured by the score $\tau^{(i,j)} \in [-1,1]$. A score of 1 or -1 indicates perfect positive or negative alignment between $X^{(i)}$ and $X^{(j)}$ respectively; whereas 0 indicates no correlation. We calculated the Kendall's $\tau$ with the `.corr()` function of the `Python`[18] package of `Pandas`[32].

## 4.2 Correlation Between Variables

Given the synthetic dataset $D_{\text{syn}}$ and the real dataset $D_{\text{real}}$, the Kendall rank correlation scores were used to determine whether correlations between any pair of variables (based on data from all patients and timepoints) were captured in the generated data.

## 4.3 Average Correlation in Trends and in Cycles

The correlations in this section are used to investigate the behaviour of the generated data over time. Our datasets comprise many patients, each of whom is associated with a specific trajectory over time. Thus, correlations over time were computed individually for every patient and pair of variables. Afterwards, these correlations were averaged across patients.

To elaborate, for a specific patient $p_k$ we selected a pair of variables ${}_{p_k}X^{(i)}$ and ${}_{p_k}X^{(j)}$. We assumed that they could be linearly decomposed into trends and cycles:

$$ {}_{p_k}X^{(i)} = \text{Trend}({}_{p_k}X^{(i)}) + \text{Cycle}({}_{p_k}X^{(i)}) \text{ and} \tag{9} $$

$$ {}_{p_k}X^{(j)} = \text{Trend}({}_{p_k}X^{(j)}) + \text{Cycle}({}_{p_k}X^{(j)}). \tag{10} $$

We implemented Cycle() with the `signal.detrend()` function of the `Python`[18] package of `Scipy`[19]; whereas Trend() is simply the remainder of $X - \text{Cycle}(X)$. By using the `signal.detrend()` function, we assumed that each time series has a linear trend. Alternatively, non-linear trends over time could be determined using moving average algorithms.

We repeat this process for every patient $p_k$ where $k = 1, \ldots, n$, and average the correlation scores for trends and cycles (the number of patients in the synthetic dataset might be different from the one in the real dataset, since the GAN can be used to generate any amount of data). A pseudo-code for computing the average correlations in trends and cycles is provided in Algorithm 2 for additional clarity.

## 4.4 Remarks on Kendall's Rank Correlation and Alternative Validation Metrics

In this study, we conducted the technical validation using Kendall's rank correlation instead of the more common *Pearson's r correlation*[33]. This was because of two reasons. First, Pearson's correlation requires all test variables to be normally distributed[34]. However, our datasets (both the synthetic and the real) included several variables with long tail distributions which could not be power-transformed easily. Second and unlike Pearson's correlation, Kendall's rank correlation could be used for both binary and categorical variables. Furthermore, Kendall's rank correlation was even applicable in scenarios where we needed to test the relations between a multi-class variable and a binary-class variable.

Besides correlations, we also considered other metrics for validating the quality of the generated synthetic data. For example, *auto-correlations* and *cross-correlations*[35] would be useful to better understand the properties of the generated time series. However, they do not appear suitable to assess *the similarity between patterns in two time series from two datasets*. Similarly, the *Wilcoxon-Mann-Whitney*[36] test would be helpful to determine if two variables are independent; however, our focus was on *the similarity of relations between variables from two datasets*.

Our validation setup also differs from the work of Goncalves *et al.*[37]. In their study, Goncalves *et al.* employed log-cluster[38] and the Kullback–Leibler (KL) divergence[39] to compare the relative similarity of synthetic datasets generated by different models against their real "ground truth" dataset. In our study, we compared synthetic datasets generated using one GAN model to the real datasets.

**Algorithm 2** The Psuedo-Code for the Average Correlations

$\{\}_{\text{trend:syn}}, \{\}_{\text{cycle:syn}}, \{\}_{\text{trend:real}}, \{\}_{\text{cycle:real}} = \emptyset$ ▷ Create storage to record correlation scores.

**for** $k = 1, n$ **do** ▷ Loop over each patient.

  **for** $i = 1, N$ **do** ▷ Select a variable $i$.

    $\{\}^{\text{k, i}}_{\text{trend:syn}}, \{\}^{\text{k, i}}_{\text{cycle:syn}}, \{\}^{\text{k, i}}_{\text{trend:real}}, \{\}^{\text{k, i}}_{\text{cycle:real}} = \emptyset$ ▷ Create a separate storage for patient-variables pair.

    **for** $j = 1, (i-1)$ **do** ▷ And select another variable $j$.

      $_{p_k}X^{(i)}_{\text{syn}} = \text{Trend}(_{p_k}X^{(i)}_{\text{syn}}) + \text{Cycle}(_{p_k}X^{(i)}_{\text{syn}})$ ▷ Decompose the selected time series

      $_{p_k}X^{(j)}_{\text{syn}} = \text{Trend}(_{p_k}X^{(j)}_{\text{syn}}) + \text{Cycle}(_{p_k}X^{(j)}_{\text{syn}})$

      $_{p_k}X^{(i)}_{\text{real}} = \text{Trend}(_{p_k}X^{(i)}_{\text{real}}) + \text{Cycle}(_{p_k}X^{(i)}_{\text{real}})$

      $_{p_k}X^{(j)}_{\text{real}} = \text{Trend}(_{p_k}X^{(j)}_{\text{real}}) + \text{Cycle}(_{p_k}X^{(j)}_{\text{real}})$

      $\tau^{(i,j)}_{\text{trend:syn}} = \tau\left(\text{Trend}(X^{(i)}_{\text{syn}}), \text{Trend}(X^{(j)}_{\text{syn}})\right)$ ▷ Compute the respective correlation scores in trend.

      $\tau^{(i,j)}_{\text{trend:real}} = \tau\left(\text{Trend}(X^{(i)}_{\text{real}}), \text{Trend}(X^{(j)}_{\text{real}})\right)$

      $\tau^{(i,j)}_{\text{cycle:syn}} = \tau\left(\text{Cycle}(X^{(i)}_{\text{syn}}), \text{Cycle}(X^{(j)}_{\text{syn}})\right)$ ▷ And compute the respective correlation scores in cycle.

      $\tau^{(i,j)}_{\text{cycle:real}} = \tau\left(\text{Cycle}(X^{(i)}_{\text{real}}), \text{Cycle}(X^{(j)}_{\text{real}})\right)$

      $\{\}^{\text{k, i}}_{\text{trend:syn}} = \{\}^{\text{k, i}}_{\text{trend:syn}} \cup \tau^{(i,j)}_{\text{trend:syn}}$ ▷ Record the scores for each patient.

      $\{\}^{\text{k, i}}_{\text{cycle:syn}} = \{\}^{\text{k, i}}_{\text{cycle:syn}} \cup \tau^{(i,j)}_{\text{cycle:syn}}$

      $\{\}^{\text{k, i}}_{\text{trend:real}} = \{\}^{\text{k, i}}_{\text{trend:real}} \cup \tau^{(i,j)}_{\text{trend:real}}$

      $\{\}^{\text{k, i}}_{\text{cycle:real}} = \{\}^{\text{k, i}}_{\text{cycle:real}} \cup \tau^{(i,j)}_{\text{cycle:real}}$

    **end for**

  **end for**

**end for**

**for** $i = 1, N$ **do** ▷ For averaging across each patient.

  $\{\}_{\text{trend:syn}} = \{\}_{\text{trend:syn}} \cup \mathbb{E}_k(\{\}^{\text{k, i}}_{\text{trend:syn}})$ ▷ Acquire the average correlation in trends.

  $\{\}_{\text{trend:real}} = \{\}_{\text{trend:real}} \cup \mathbb{E}_k(\{\}^{\text{k, i}}_{\text{trend:real}})$

  $\{\}_{\text{cycle:syn}} = \{\}_{\text{cycle:syn}} \cup \mathbb{E}_k(\{\}^{\text{k, i}}_{\text{cycle:syn}})$ ▷ Acquire the average correlation in cycles.

  $\{\}_{\text{cycle:real}} = \{\}_{\text{cycle:real}} \cup \mathbb{E}_k(\{\}^{\text{k, i}}_{\text{cycle:real}})$

**end for**

# 5 Full Correlation Plots for Sepsis

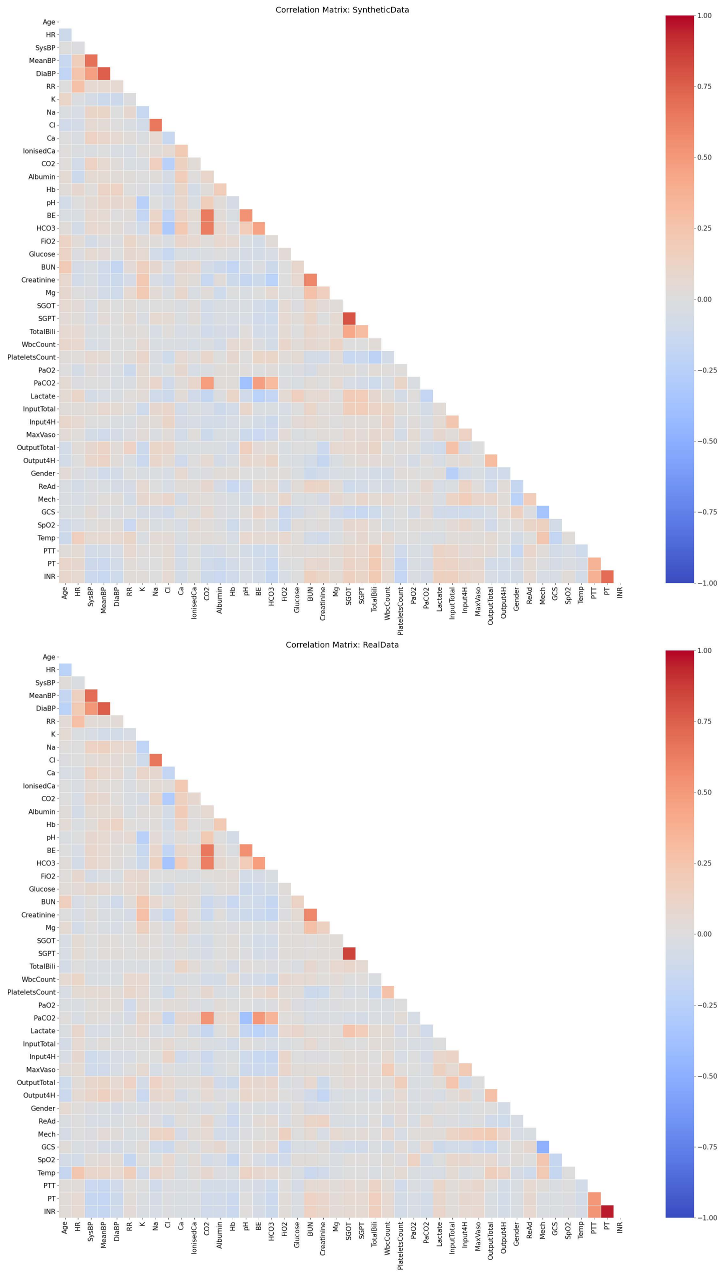


**Figure 1.** The Complete Static Correlation Plots for Sepsis

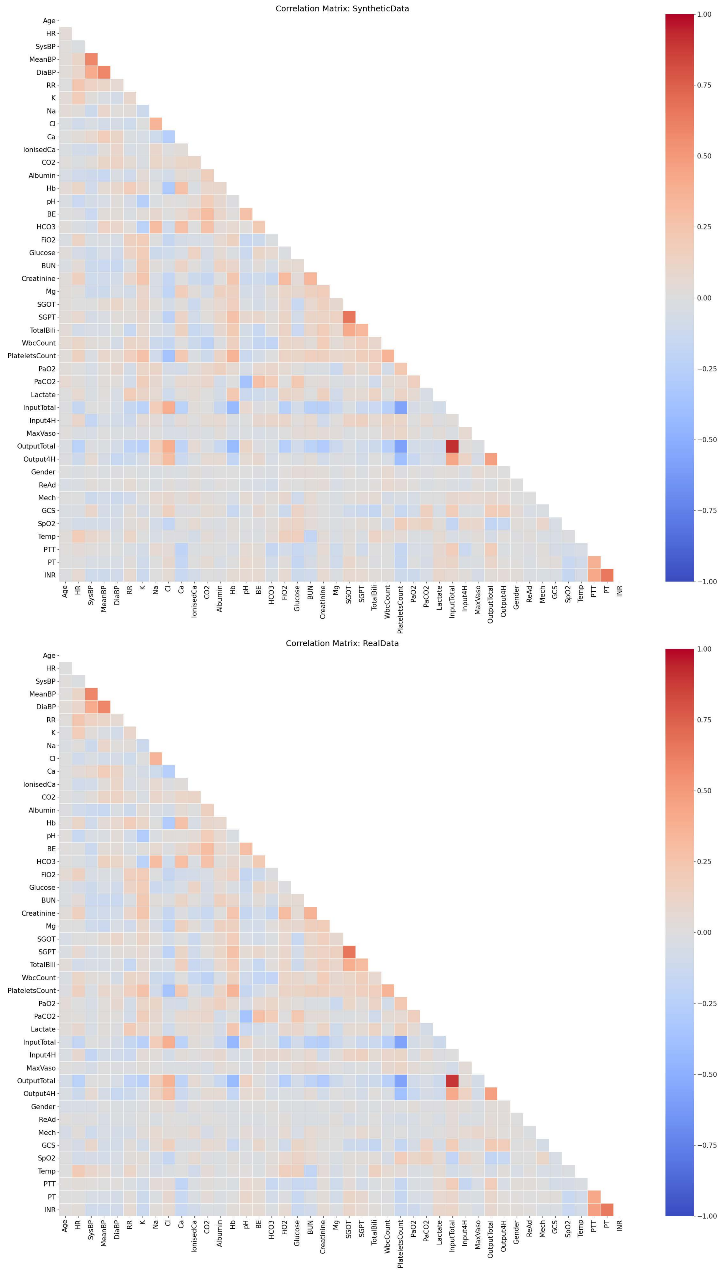


**Figure 2.** The Complete Dynamic Correlations in Trends for Sepsis

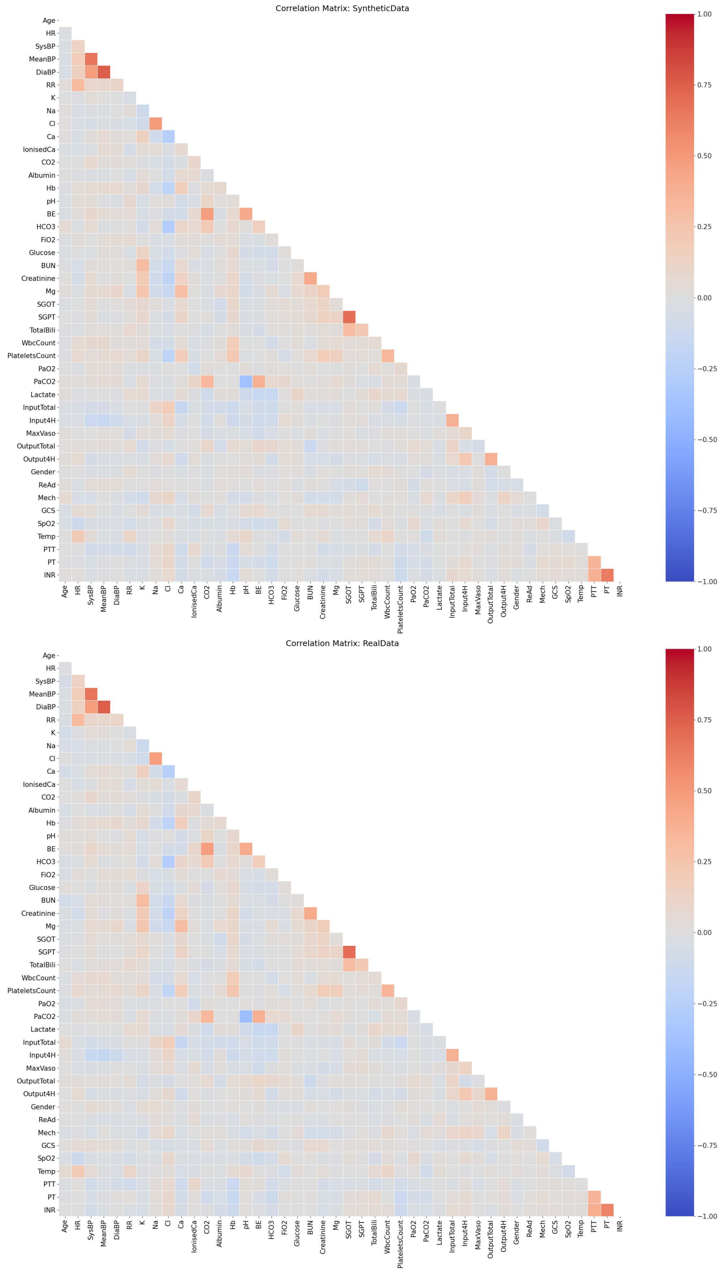


**Figure 3.** The Complete Dynamic Correlations in Cycles for Sepsis

# 6 Assessment of Disclosure Risk

This section aims to provide more details for the assessment of disclosure risk and covers some potential alternative metrics.

## 6.1 El Emam *et al.*'s Disclosure Risk Metrics

Below, we provide more intuitions to El Emam *et al.*[6]'s risk evaluation metrics. Assuming that an adversary was able to recover partial information (*i.e.,* the quasi-identifiers) of an individual (*i.e.,* the acquaintance), El Emam *et al.*[40] formulated the probability of the successful re-identification (re-id) of the information of an acquaintance as

$$\mathbb{P}(\text{re-id}, \text{acquaintance}) = \mathbb{P}(\text{acquaintance}) \times \mathbb{P}(\text{re-id}|\text{acquaintance}) \tag{11}$$

using the Bayes rule.

Based on Equation (11), their population-to-sample re-identification risk for the real dataset of

$$\underbrace{\frac{1}{P}}_{\textcircled{1}} \underbrace{\sum_{s=1}^{S}}_{\textcircled{2}} \underbrace{\left(\frac{1}{f_s}\right)}_{\textcircled{3}}$$

considers that ② for all individuals in the real sample dataset, calculate the chance of ① *any* randomly selected record from the population ③ being matched to a specific record in the real dataset with its equivalent class of quasi-identifiers known (*e.g.,* male and 21 years old). Note that component ① corresponds to $\mathbb{P}(\text{acquaintance})$ – the selection of a random record from a population of size $P$. In addition, components ② and ③ are affiliated to $\mathbb{P}(\text{re-id}|\text{acquaintance})$ because all patients with the same equivalent class of size $f_s$ (*e.g.,* 3 if there are 3 males that are 21 years old) have an equal chance of being pinpointed with accuracy $\frac{1}{f_s}$ (*i.e.,* $\frac{1}{3}$ chance for each 21 years old male to be identified).

Following the same line of thoughts, their sample-to-population re-identification risk for the real dataset of

$$\underbrace{\frac{1}{S}}_{\textcircled{4}} \underbrace{\sum_{s=1}^{S}}_{\textcircled{2}} \underbrace{\left(\frac{1}{F_s}\right)}_{\textcircled{5}}$$

describes the scenario that ② for all individuals in the real sample dataset, calculate the chance of ④ *any* randomly selected record from the real dataset ⑤ that can be traced back to a specific record in the population sharing the same equivalent class of quasi-identifiers. This time, $\mathbb{P}(\text{acquaintance})$ corresponds to component ④ with $\frac{1}{S}$ because the real dataset has a total of $S$ records; and that $\mathbb{P}(\text{re-id}|\text{acquaintance})$ is now affiliated with ⑤ with $\frac{1}{F_s}$ because the total amount of records and their equivalent classes are now sourced from the population.

In their work, El Eman *et al.*'s expanded the risk definitions to

$$\text{the population-to-sample risk} : \quad \frac{1}{P}\sum_{s=1}^{S}\left(\frac{1}{f_s} \times \lambda_s \times R_s\right) \quad \text{and} \tag{12}$$

$$\text{the sample-to-population risk} : \quad \frac{1}{S}\sum_{s=1}^{S}\left(\frac{1}{F_s} \times \lambda_s \times R_s\right). \tag{13}$$

The full expressions included two extra terms. Under the section of **Adjusting for Incorect Matches** in their paper, the authors noted that $\lambda$ was a constant term $\in (0,1]$ that scaled down the original re-identifiability for the records in the dataset. This term existed because that health data, and generally any type of data from a data broker, were known to have some error rates[41]; and $\lambda$ was introduced to correct the overestimation of meaningful adversarial re-identifications. Furthermore and under the section of **Learning Something New**, the authors noted that $R$ was a binary term and that $R = 1$ only when any matched records contributed towards learning non-duplicated re-identified information. These two extra terms thus scaled down the original population-to-sample risk and sample-to-population risk. In our own work, we left these two terms out because that the unscaled risks were already very low.

## 6.2 Alternative Disclosure Risk Metrics

In this study, we employed the Euclidean distance and the synthetic-to-real disclosure risk to investigate the risks associated with the public release of synthetic datasets. However, there are many alternative options.

Instead of the Euclidean distance, the Mahalanobis distance[42] may be well suited to the high dimensionality of electronic health records. Nonetheless, the Euclidean distance was perfectly suitable to show that there were no perfect matches between real and synthetic records.

Furthermore, El Emam *et al.*'s metrics are just two of the many available metrics in the risk disclosure literature. Both the population-to-sample risk and the sample-to-population risk can be seen as extended applications of *k-anonymity*[43] – a desirable property for a dataset implying that multiple records share the same equivalence class of quasi-identifiers, thus lowering the chance of identity exposure (*e.g.,* it is better if a released dataset contains multiple 21-year-old makes instead of just one). However, it is well-known that attributes of an anonymised dataset could still be inferred even if k-anonymity is satisfied. Attribute disclosure is especially concerning if homogeneity in patient data is high. For instance, if all 21-year-old males suffer from stomach problems and a specific 21-year-old male is the target of an adversary, then the adversary can acquire knowledge automatically without the need to match any records. Attribute disclosure can be mitigated by achieving *l-diversity*[44] and/or *t-closeness*[45] in addition to k-anonymity. The former aims to increase the number of sensitive attributes within each equivalence class; whereas the latter uses information theoretic measures to verify if the records satisfy a minimal level of diversification.

Aside from Euclidean distances and disclosure risks, a practitioner could also test the security of their synthetic datasets by building predictive models. For instance, Choi *et al.*[46] used k nearest neighbours to predict a varying number of known attributes in the datasets. This could be helpful to determine whether any known sensitive variables could be easily reconstructed through the quasi-identifiers.

# 7 Verifying Utility through Reinforcement Learning

As mentioned under **Utility Verification** in the **Technical Validation** section, we partitioned each dataset (both real and synthetic) into the action subset $\mathscr{D}_A$ and the observational subset $\mathscr{D}_O$. For each health condition, we reserved two categorical variables for the action subset, and the remaining variables formed the observational subset.

The variables reserved for the action subsets $\mathscr{D}_A$ depended on the health condition. They were

- Fluid Boluses (with 4 classes) and Vasopressors (with 4 classes) for acute hypotension;
- Input 4H (with 4 classes) and Max Vaso (with 4 classes) for sepsis; and
- Base Drug Combo (with 6 classes) and Comp. NNRTI (with 4 classes) for HIV.

The action spaces hence included 16, 16, and 24 actions for acute hypotension, sepsis, and HIV, respectively.

We derived the states in the RL environments using cross decomposition[47] followed by K-Means clustering. Cross decomposition was applied to reduce the dimensionality of $\mathscr{D}_O$ to 5; then, we used the associated clusters of K-Means to label the datapoints in $\mathscr{D}_O$. The amounts of clusters were set to 100 for all conditions.

After defining the states and actions for the management of patient conditions, we trained our RL agents using batch-constrained Q-learning[48]. The policy was updated for 100 iterations with step size 0.01. We updated the agent policy according to published reward functions, and deep learning was not employed. For future work, we aim to employ more advanced algorithms to further evaluate the utility of the synthetic datasets. Interested readers can refer to our HealthGym website for a tutorial on the RL setup (see https://healthgym.ai/gettingstarted/).

## 7.1 The Acute Hypotension Reward Function

We adopted the reward function from Appendix D.6 on page 17 in the work of Gottesman *et al.*[10] for training an RL agent for the management of acute hypotension. The reward at time step $t$ $\texttt{reward}_t$ is dependent on the Mean Arterial Pressure $\texttt{MAP}_t$ and is given as

$$\texttt{reward}_t = \begin{cases} 0, & \texttt{MAP}_t > 65 \\ -0.05 \ \ (65 - \texttt{MAP}_t)/5, & 60 < \texttt{MAP}_t \leq 65 \\ -0.10 \ \ (60 - \texttt{MAP}_t)/5 - 0.05, & 55 < \texttt{MAP} \leq 60 \\ -0.8 \ \ 5(55 - \texttt{MAP}_t)/15 - 0.15, & \texttt{MAP}_t \leq 55 \end{cases} \tag{14}$$

but the reward value is also dictated, and overwrote, by the Urine output $\texttt{urine}_t$ of a patient when

$$\texttt{reward}_t = 0 \quad \text{if} \quad \texttt{urine}_t > 30 \text{ and } \texttt{MAP}_t > 55. \tag{15}$$

## 7.2 The Sepsis Reward Function

We adopted the reward function from Appendix 7.2 on page 8 in the work of Raghu *et al.*[49] for training an RL agent for the management of sepsis. The reward function is computed in three parts such that

$$\texttt{reward}_t = \texttt{reward}_t^{(1)} + \texttt{reward}_t^{(2)} + \texttt{reward}_t^{(3)} \quad \text{where} \tag{16}$$

$$\texttt{reward}_t^{(1)} = -0.025 \quad \text{if } \texttt{SOFA}_t = \texttt{SOFA}_{t-1} \text{ and } \texttt{SOFA}_t > 0, \tag{17}$$

$$\texttt{reward}_t^{(2)} = -0.125 \ \ \texttt{SOFA}_t - \texttt{SOFA}_{t-1}, \quad \text{and} \tag{18}$$

$$\texttt{reward}_t^{(3)} = -2 \ \ \tanh(\texttt{Lactate}_t - \texttt{Lactate}_{t-1}). \tag{19}$$

Raghu *et al.*'s reward function penalises high *Sequential Organ Failure Assessment scores* (SOFA) $\texttt{SOFA}_t$ and high Lactate $\texttt{Lactate}_t$. On one hand, the sofa score predicts ICU mortality based on lab results and clinical data; and on the other hand, a patient's lactate level is a measurement of cell-hypoxia and it is higher in septic patients because sepsis-induced low blood pressure reduces oxygen perfusion into tissue.

Note, Raghu *et al.*'s original sepsis reward function also takes patient survival into account (with $+15$ if a patient survived their ICU stay and $-15$ if they did not). This was not included in Equation (16) because our synthetic datasets do not include information on patient mortality. Further note that the SOFA score is not explicitly included in our synthetic sepsis dataset; however, it can be easily derived from PaO2, FiO2, Platelets, Total Bili, Mean BP, Max Vaso, GCS, Creatinine, and Output 4H (see Tables 2 and 3 in the main text). For code implementation, interest readers may refer to lines 820 – 840 of `AIClinician_sepsis3_def_160219.m` in Komorowski *et al.*[2]'s repository at https://gitlab.doc.ic.ac.uk/AIClinician/AIClinician/-/tree/master/.

## 7.3 The HIV Reward Function

We adapted the reward function from page 5 in the work of Parbhoo *et al.*[3] for training an RL agent for the management of HIV. The reward function is given as

$$\texttt{reward}_t = \begin{cases} -0.7\ \log \texttt{VL}_t + 0.6\ \log \texttt{CD4}_t, & \text{if } \texttt{VL}_t \text{ is above detection limits, and} \\ 5 + 0.6\ \log \texttt{VL}_t & \text{if } \texttt{VL}_t \text{ is below detection limits.} \end{cases} \quad (20)$$

There is a small detail worth mentioning: while our synthetic data use cells/$\mu L$ as the unit for $\texttt{CD4}_t$ count (see Table 4 in the main text), Parbhoo *et al.* use cells/mL as their unit for the reward computation in Equation (20).

Furthermore, Parbhoo *et al.*'s original reward function also factors in the number of mutations in the HIV viral genome associated with drug resistance. This information is omitted in Equation (20) because it is currently unavailable in our synthetic HIV dataset.

## References


1. Gottesman, O. *et al.* Guidelines for reinforcement learning in healthcare. *Nat. Med.* **25**, 16–18 (2019).
2. Komorowski, M., Celi, L. A., Badawi, O., Gordon, A. C. & Faisal, A. A. The artificial intelligence clinician learns optimal treatment strategies for sepsis in intensive care. *Nat. Med.* **24**, 1716–1720 (2018).
3. Parbhoo, S., Bogojeska, J., Zazzi, M., Roth, V. & Doshi-Velez, F. Combining kernel and model based learning for hiv therapy selection. *AMIA Jt. Summits Transl. Sci. Proc.* **2017**, 239 (2017).
4. World Health Organisation. Consolidated guidelines on the use of antiretroviral drugs for treating and preventing hiv infection: Recommendations for a public health approach (2016).
5. Goodfellow, I. *et al.* Generative adversarial nets. In *the Advances in Neural Information Processing Systems* (2014).
6. El Emam, K., Mosquera, L. & Bass, J. Evaluating identity disclosure risk in fully synthetic health data: Model development and validation. *J. Med. Internet Res.* **22**, 23139 (2020).
7. Johnson, A. E. *et al.* Mimic-iii, a freely accessible critical care database. *Sci. Data* **3**, 1–9 (2016).
8. Johnson, A., Pollard, T. & Mark III, R. Mimic-iii clinical database (version 1.4). *PhysioNet* (2016), https://doi.org/10.13026/C2XW26.
9. Zazzi, M. *et al.* Predicting response to antiretroviral treatment by machine learning: The euresist project. *Intervirology* **55**, 123 - 127 (2012).
10. Gottesman, O. *et al.* Interpretable off-policy evaluation in reinforcement learning by highlighting influential transitions. In *the International Conference on Machine Learning*, 3658–3667 (2020).
11. Kawasaki, Z., Shibata, K. & Tajima, M. A guide to the sql standard: A user's guide to the standard database language sql a guide to the sql standard: A user's guide to the standard database language sql, 1997. *IEICE Trans. Inf. & Syst.* **86**, 1139–1143 (2003).
12. Teasdale, G. & Jennett, B. Assessment of coma and impaired consciousness: A practical scale. *The Lancet* **304**, 81–84 (1974).
13. Singer, M. *et al.* The third international consensus definitions for sepsis and septic shock (sepsis-3). *JAMA* **315**, 801–810 (2016).
14. MATLAB. *version 7.10.0 (R2010a)* (The MathWorks Inc., 2010).
15. Group, I. S. S. Initiation of antiretroviral therapy in early asymptomatic hiv infection. *NEJM* **373**, 795–807 (2015).
16. Arjovsky, M., Chintala, S. & Bottou, L. Wasserstein generative adversarial networks. In *the International Conference on Machine Learning*, 214–223 (2017).
17. Box, G. E. & Cox, D. R. An analysis of transformations. *J. R. Stat. Soc. Ser. B Stat. Methodol.* **26**, 211–243 (1964).
18. Van Rossum, G. & Drake Jr, F. L. *Python reference manual* (Centrum voor Wiskunde en Informatica Amsterdam, 1995).
19. Virtanen, P. *et al.* Scipy 1.0: Fundamental algorithms for scientific computing in python. *Nat. Methods* **17**, 261–272 (2020).
20. Pedregosa, F. *et al.* Scikit-learn: Machine learning in python. *J. Mach. Learn. Res.* **12**, 2825–2830 (2011).
21. Gulrajani, I., Ahmed, F., Arjovsky, M., Dumoulin, V. & Courville, A. C. Improved training of wasserstein gans. In Guyon, I. *et al.* (eds.) *the Advances in Neural Information Processing Systems* (2017).
22. Kingma, D. P. & Ba, J. Adam: A method for stochastic optimisation. In *the International Conference on Learning Representations* (2015).
23. Bengio, Y., Louradour, J., Collobert, R. & Weston, J. **Curriculum learning**. In *the International Conference on Machine Learning* (2009).
24. Press, O., Bar, A., Bogin, B., Berant, J. & Wolf, L. Language generation with recurrent generative adversarial networks without pre-training (2017). Preprint at https://arxiv.org/abs/1706.01399.
25. Kolmogorov, A. Sulla determinazione empirica di una lgge di distribuzione. *Inst. Ital. Attuari, Giorn.* **4**, 83–91 (1933).
26. Smirnov, N. Table for estimating the goodness of fit of empirical distributions. *The Ann. Math. Stat.* **19**, 279–281 (1948).
27. "Student" Gosset, W. S. The probable error of a mean. *Biometrika* 1–25 (1908).
28. Johnson, N. L., Kotz, S. & Balakrishnan, N. *Continuous Univariate Distributions*, vol. 289 (John Wiley & Sons, 1995).

**29.** Rumelhart, D. E., Hinton, G. E. & Williams, R. J. Learning representations by back-propagating errors. *Nature* **323**, 533–536 (1986).

**30.** Bonferroni, C. Teoria statistica delle classi e calcolo delle probabilita. *Pubblicazioni del R Istituto Super. di Scienze Econ. e Commericiali di Firenze* **8**, 3–62 (1936).

**31.** Kendall, M. G. The treatment of ties in ranking problems. *Biometrika* **33**, 239–251 (1945).

**32.** McKinney, W. Data structures for statistical computing in python. In *the Proceedings of Python in Science Conference*, 51–56 (2010).

**33.** Mukaka, M. M. A guide to appropriate use of correlation coefficient in medical research. *Malawi Med. J.* **24**, 69–71 (2012).

**34.** Kowalski, C. J. On the effects of non-normality on the distribution of the sample product-moment correlation coefficient. *J R Stat Soc Ser C Appl Stat* **21**, 1–12 (1972).

**35.** Bracewell, R. N. & Bracewell, R. N. *The Fourier Transform and Its Applications*, vol. 31999 (McGraw-Hill New York, 1986).

**36.** Mann, H. B. & Whitney, D. R. On a test of whether one of two random variables is stochastically larger than the other. *The Ann. Math. Stat.* 50–60 (1947).

**37.** Goncalves, A. *et al.* Generation and evaluation of synthetic patient data. *BMC Med. Res. Methodol.* **20**, 1–40 (2020).

**38.** Woo, M.-J., Reiter, J. P., Oganian, A. & Karr, A. F. Global measures of data utility for microdata masked for disclosure limitation. *J. Priv. Confidentiality* **1** (2009).

**39.** Kullback, S. & Leibler, R. A. On information and sufficiency. *The Ann. Math. Stat.* **22**, 79–86 (1951).

**40.** El Emam, K. & Malin, B. Concepts and methods for de-identifying clinical trial data. *The Comm. on Strateg. for Responsible Shar. Clin. Trial Data* (2014).

**41.** Elliot, M. & Dale, A. Scenarios of attack: The data intruder's perspective on statistical disclosure risk. *Neth. Off. Stat.* **14**, 6–10 (1999).

**42.** De Maesschalck, R., Jouan-Rimbaud, D. & Massart, D. L. The mahalanobis distance. *Chemom. Intell. Lab. Syst.* **50**, 1–18 (2000).

**43.** Samarati, P. Protecting respondents identities in microdata release. *IEEE Trans. Knowl. Data Eng.* **13**, 1010–1027 (2001).

**44.** Machanavajjhala, A., Kifer, D., Gehrke, J. & Venkitasubramaniam, M. l-diversity: Privacy beyond k-anonymity. *ACM Trans. Knowl. Discov. Data* **1**, 3 (2007).

**45.** Li, N., Li, T. & Venkatasubramanian, S. t-closeness: Privacy beyond k-anonymity and l-diversity. In *the International Conference on Data Engineering*, 106–115 (2007).

**46.** Choi, E. *et al.* Generating multi-label discrete patient records using generative adversarial networks. In *the Machine Learning for Healthcare Conference*, 286–305 (2017).

**47.** Wegelin, J. A. A survey of partial least squares (pls) methods, with emphasis on the two-block case. Tech. Rep., University of Washington (2000).

**48.** Fujimoto, S., Meger, D. & Precup, D. Off-policy deep reinforcement learning without exploration. In *the International Conference on Machine Learning*, 2052–2062 (2019).

**49.** Raghu, A. *et al.* Deep reinforcement learning for sepsis treatment (2017). Preprint at https://arxiv.org/abs/1711.09602.
